# Redesign of ultrasensitive and robust RecA gene circuit to sense DNA damage

**DOI:** 10.1111/1751-7915.13767

**Published:** 2021-03-04

**Authors:** Jack X. Chen, Boon Lim, Harrison Steel, Yizhi Song, Mengmeng Ji, Wei E. Huang

**Affiliations:** ^1^ Department of Engineering Science University of Oxford Parks Road Oxford OX1 3PJ UK; ^2^ Oxford Suzhou Centre for Advanced Research Suzhou 215123 China

## Abstract

SOS box of the *recA* promoter, P_VRecA_ from *Vibrio natriegens* was characterized, cloned and expressed in a probiotic strain *E. coli* Nissle 1917. This promoter was then rationally engineered according to predicted interactions between LexA repressor and P_VRecA_. The redesigned P_VRecA‐AT_ promoter showed a sensitive and robust response to DNA damage induced by UV and genotoxic compounds. Rational design of P_VRecA_ coupled to an amplification gene circuit increased circuit output amplitude 4.3‐fold in response to a DNA damaging compound mitomycin C. A TetR‐based negative feedback loop was added to the P_VRecA‐AT_ amplifier to achieve a robust SOS system, resistant to environmental fluctuations in parameters including pH, temperature, oxygen and nutrient conditions. We found that *E. coli* Nissle 1917 with optimized P_VRecA‐AT_ adapted to UV exposure and increased SOS response 128‐fold over 40 h cultivation in turbidostat mini‐reactor. We also showed the potential of this P_VRecA‐AT_ system as an optogenetic actuator, which can be controlled spatially through UV radiation. We demonstrated that the optimized SOS responding gene circuits were able to detect carcinogenic biomarker molecules with clinically relevant concentrations. The ultrasensitive SOS gene circuits in probiotic *E. coli* Nissle 1917 would be potentially useful for bacterial diagnosis.

## Graphical abstract

Rational design of SOS promoter to create ultra‐sensitive and robust recA gene circuit to sense DNA damage. The ultrasensitive SOS gene circuits in probiotic *E. coli* Nissle 1917 would be potentially useful for bacterial diagnosis.

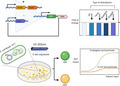

## Introduction

Naturally occurring microorganisms living on and in the human body present an opportunity for *in situ* bacterial diagnostics and therapy (Kurtz *et al*., 2019; Forbes, 2010). Despite advancements in treating gut inflammation (Forbes, 2010; Archer *et al*., 2012; Kurtz *et al*., 2019), hyperammonemia (Kurtz *et al*., 2019), obesity (Chen *et al*., 2014) and various cancer treatments (Ganai *et al*., 2009; He *et al*., 2017; Yu *et al*., 2019), bacterial therapy still lacks accurate control systems that can act as ‘input modules’. Synthetic biology could contribute to the development of inexpensive, rapidly deployable bacterial diagnostics and therapy (Slomovic *et al*., 2015). An ideal module to bacterial diagnostics and therapy should be highly sensitive to low dosage of inducer; low baseline with minimal background expression; strongly responsive to produce readily detectable outputs; stable to resist nutrient and environmental fluctuations, spatially controllable to enable a localized activation. Finally, the engineered host bacteria containing the module should be clinically safe.

Some carcinogenic or DNA damaging compounds generated in the human body are responsible for cancers (Seitz and Becker, 2007; Ridlon *et al*., 2016; Nguyen *et al*., 2018). The SOS response system is a global response to such DNA damage and represents a universal regulation mechanism in bacteria (Cohen *et al*., 2008; Simons *et al*., 2010), yeast (Fu *et al*., 2008) and humans (Michel, 2005). The response mechanism of the SOS system showed significant overlap in different prokaryotic organisms (Erill *et al*., 2007; Kreuzer, 2013). However, the wild‐type *E. coli* SOS system is not sufficiently sensitive or rapid for diagnosis of cancer biomarker molecules. Therefore, a sensitive, low background, strong responsive, stable and spatially controllable SOS system is required for the application of bacterial diagnostics and therapy.

The Gram‐negative and non‐pathogenic *Vibrio natriegens* are the fastest growing bacterium that has been isolated (Lee et al*.,* 2016). First reported in the early 1960s, *V. natriegens* was found to have a doubling time of fewer than 10 min under optimal conditions, which is less than half of the shortest reported doubling time of *E. coli* recorded in the literature (Erill *et al*., 2007). Considering its fast growth rate (Lee et al*.,* 2016), it is expected that *V. natriegens* may be equipped with a robust and sensitive SOS system for DNA repairing in short time due to natural errors of DNA replication. The expression of the universal SOS response regulator, RecA, in response to UV radiation by *V. natriegens* was previously reported (Booth *et al*., 2001). However, the response time and sensitivity of the *recA* promoter of *V. natriegens* to DNA damage remain unknown.

In this study, we first identified LexA binding sites of *V. natriegens recA* gene promoter (P_VRecA_) according to the predicted promoter‐LexA interactions. The transcriptional sigma factor, LexA binding affinity and interactions with the P_VRecA_ promoter were then characterized by mutagenesis. We then rationally designed a spacer between LexA binding sites in P_VRecA_ and tested its performance to sense DNA damage, such as carcinogenic chemicals and UV radiation. To demonstrate the promoter modularity and to further strengthen the tightness of the control, we created a series of chimeric promoters and examined the performance of the redesigned recA promoter.

Natural heterogeneity, noise and variability of gene expression at a constantly changing environment are challenges for bacteria therapy. The physiology condition in human tissue (pH, oxygen and nutrient availability) (Courbet *et al*., 2015) can alter the dynamic range of synthetic circuits, thus undermining an engineered system’s performance. Inspired by recent work on disturbance‐resistant control systems (Aoki *et al*., 2019), we further improved the stability and sensitivity of the SOS system through a designed negative feedback loop, which is an integrated HrpRS‐based transcriptional amplifier and TetR‐based repression system (Wang *et al*., 2014). This operational feedback design was demonstrated to improve the robustness of the sensing circuit when subjected to different environmental changes.

We further demonstrated that the optimized system could be used as an optogenetic tool, which uses light to control gene expression and have broad applications in synthetic biology. Optogenetic control is particularly attractive because of its ability to enable remote (i.e. non‐contact), precise (predictable input‐output response) and rapid actuation of gene expression (Olson *et al*., 2014; Zhao *et al*., 2018; Aoki *et al*., 2019). This optimized SOS system can be can be precisely controlled by UV light.

All circuits were tested and validated in *E.coil* Nissle 1917 due to its established safety and efficacy in clinical applications (Sarate *et al*., 2019; Crook *et al*., 2019) and genomic similarities with other *E*. *coli* variants (Grozdanov *et al*., 2004). Overall, we developed a toolbox for SOS gene circuit for the detection of DNA damage, which can detect a range of disease biomarker molecules and carcinogenic antibiotics in a sensitive and robust manner.

## Result

### *Cloning recA promoter from V. natriegens into E. coli* Nissle *1917 for SOS response*

**Fig. 1:**
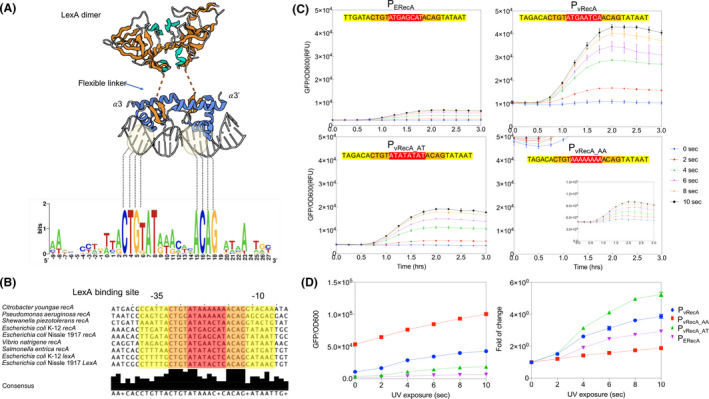
Binding of LexA and *recA/lexA* promoter. A. WebLogo diagram of consensus LexA binding site compiled between six bacteria species, the consensus sequence for SOS box is (CTGTN_8_ACAG). In the 3D structure interaction, DNA base pair interaction with LexA dimer was highlighted in orange box where each of the motif base pair interact with the α helix domain directly. 3D crystal structure was acquired from (UniProt ID P0A7C2). B. Sequence alignment of the highly conserved region of LexA binding site (Orange), Sigma factor binding site (Yellow) and 8‐bp spacer (Red) among nine promoters from different species. C. Circuit performance with different SOS binding box sequence. Sequence alignment of SOS box of *E*. *coli rec*A promoter and three variants of *V*. *natriegens rec*A promoter. GFP induction of all three v. RecA variants of 3 h under induction of UV. Sigma factor binding site −35 (TAGACA) and −10 (TATAAT) was highlighted in yellow and LexA binding site highlighted in orange. D. Dosage response and induction fold of change profile for each construct under the exposure of UV. Each experiment has three biological and three measurement replicates.

A schematic diagram of the SOS DNA repair mechanism is shown in Fig. S1A. A LexA dimer binds the SOS box and represses the promoter of genes related to DNA repair (Fig. 1A), including the *recA* gene. When DNA damage occurs, the single‐stranded (ssDNA) from mismatched DNA and RecA protein form a complex (ssDNA‐RecA), which induces the self‐cleavage of LexA (Butala *et al*., 2009), resulting in de‐repressing of gene expression in the SOS machinery (Fig. S1B). Alignment of *recA* promoters in various bacteria showed that the binding sites of LexA dimer are highly conserved CTGT‐N8‐ACAG which is flanked by −35 and −10 sigma binding site (Fig. 1A and B). Sequence alignment of *V. natriegens* and *E. coli* indicated a high similarity in both conserved LexA and sigma factor binding sites (Table S1). Sequence alignment of other *Vibrio fischeri* (˜280 min) exhibited similar conserved region but different upper/lower flanking sequences (Table S2). It implicates that the flanking region of LexA binding motifs could affect the response time of SOS. *V. natriegens* and *E. coli* Nissle 1917 share 72% homology in LexA protein sequence and the peptide sequence of putative binding site is similar (Fig. S1C) (Little *et al*., 1981; Brent and Ptashne, 1981). It suggests that LexA in *E. coli* Nissle 1917 could recognize a similar SOS box in *Vibrio natriegens rec*A (*vrecA*) gene promoter.

All strains and plasmids in this study are listed in Table 1. We constructed a plasmid carrying P_VRecA_ (*V. natriegens recA* promoter, NCBI ref: NZ_CP016347.1) promoter to control the expression of a superfolder GFP (*sfGFP*) gene (Pédelacq *et al*., 2006) in *E. coli* Nissle 1917 (Fig. S2B). To make the gene circuit comparable, we constructed a basic P_ERecA_ promoter with native *E. coli* Nissle 1917 promoter of *recA* gene using the same ribosome binding site (RBS, AAAGAGGAGAAA, BBa_B0030, parts.igem.org) and the gap sequence GGTACC between RBS and ATG of *sfGFP* gene (Fig. S2). Fig. 1C shows that the P_VRecA_ promoter was more sensitive and had a higher expression amplitude than the *E. coli* native promoter P_ERecA_. The P_VRecA_ promoter can be triggered by UV with exposure time as short as 2 s, while the *E. coli* native recA promoter P_ERecA_ required at least 4 s (Fig. 1C). The fold change of P_VRecA_ promoter was significantly higher than P_ERecA_ (Fig. 1D). UV exposure durations up to 10 s did not affect cell growth compared to a control without UV exposure in the testing conditions (Fig. S3). However, P_VRecA_ also demonstrated a higher leakiness than the P_ERecA_ (Fig. 1C).

**Fig. 2:**
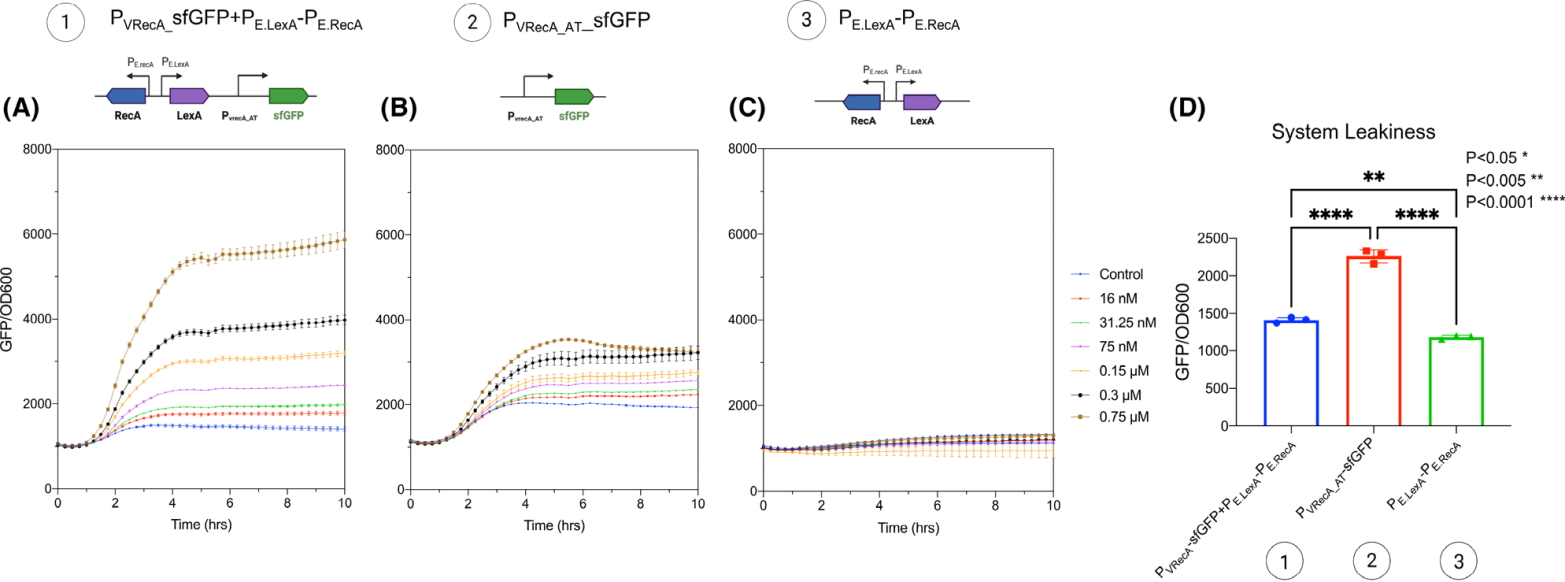
Gene circuit performance with addition of LexA and RecA module cloned from *E. coli* Nissle 1917. A. Genes of *lex*A and *rec*A with original promoters were cloned into P_vrecA_AT_ system and tested in *E. coli* Nissle 1917 B) P_vrecA_AT_ system and C) P_E‐recA_PE‐lexA_ with no sfGFP. D. The baseline leakiness for each system (A, B and C) was compared using pair t tests. Each experiment has three biological and three measurement replicates.

Genes of *rec*A and *lex*A with their native promoters of *E. coli* Nissle 1917 were cloned into a plasmid to make P_VRecA‐AT_ ‐sfGFP. The results demonstrated that the sensitivity to mitomycin C has been increased (Fig. 2A and B), and the baseline has been significantly repressed due to the expression of extra *lex*A on P_VRecA‐AT_ ‐sfGFP compared to the cases without (Fig. 2D). This confirms that LexA from *E.coli* Nissle 1917 can indeed bind with P_VRecA‐AT_ promoter from *V. natriegens* and repress baseline expression.

### Characterizing the SOS box of V. natriegens recA promoter

To further analyse the P_VRecA_ promoter, we used the LexA crystal structure and LexA‐promoter interaction model (Zhang *et al*., 2010) to annotate P_VRecA_ promoter. We identified the SOS box in P_VRecA_ promoter, which is a 16‐bp sequence (CTGTATGAGCATACAG) flanked by sigma factor binding sites, −35 (TAGACA) and −10 (TATAAT) (Supplementary information). Table 1 shows that the structure of the SOS box in *V. natriegens* (P_VRecA_) is similar to that in *E. coli* Nissle 1917 (P_ERecA_), and their LexA binding sites are identical, recognizing the reverse complementary CTGT/ACAG with 8 bp spacer in between (CTGT‐N_8_‐ACAG).

**Table 1:** The strains and plasmids used in this study.

Strains	Genotype
*E.coli* Nissle 1917 (EcN)	Ardeypharm GmbH (Herdecke, Germany)
*E.coli* DH5a	fhuA2 Δ(argF‐lacZ)U169 phoA glnV44 Φ80 Δ(lacZ)M15 gyrA96 recA1 relA1 endA1 thi‐1 hsdR17
*E.coli* BL21(DE3)	*fhuA2 [lon] ompT gal (λ DE3) [dcm] ∆hsdS λ DE3 = λ sBamHIo ∆EcoRI‐B int::(lacI::PlacUV5::T7 gene1) i21 ∆nin5*

*E.coli* JM109	*endA1, recA1, gyrA96, thi, hsdR17 (r_k_^–^, m_k_^+^), relA1, supE44, Δ( lac‐proAB), [F´ traD36, proAB, laqI^q^ZΔM15]*.


Plasmids Name	Description	Resistance	ori
P_VRecA___AT_‐GFP	P_VRecA_ modified (Four AT repeats) promoter fused with orf GFP (pMK backbone)	Kan^R^	p15A
P_VRecA__sfGFP	P_VRecA_ wild‐type promoter fused with orf sfGFP(pMK backbone)	Kan^R^	ColE1
P_VRecA___AT__sfGFP	P_VRecA_ modified (Four AT repeats) promoter fused with orf super fold GFP(pMK backbone)	Kan^R^	ColE1
P_VRecA___AA__sfGFP	P_VRecA_ modified (Eight A tandem) promoter fused with orf super fold GFP(pMK backbone)	Kan^R^	ColE1
P_ELexA_ ‐P_ERecA_	*recA* and *LexA orf* cloned under its original promoter from *E. coli* Nissle 1917	Carb^R^	ColE1
P_VRecA__sfGFP + P_E. LexA_‐P_E. RecA_	P_ELexA_ ‐P_ERecA_ constructs integrated with P_VRecA_AT__sfGFP	Carb^R^	ColE1
P_VRecA___Jungle_Down__sfGFP	P_VRecA_ modified (Double SOS) promoter fused with orf super fold GFP, additional SOS placed 7bp downstream(pMK backbone)	Kan^R^	ColE1
P_VRecA___Jungle_Up__sfGFP	P_VRecA_ modified (Double SOS) promoter fused with orf super fold GFP, additional SOS placed 7bp Upstream(pMK backbone)	Kan^R^	ColE1
P_VRecA___Jungle_Both__sfGFP	P_VRecA_ modified (Triple SOS) promoter fused with orf super fold GFP. Additional SOS placed both up‐ and downstream(pMK backbone)	Kan^R^	ColE1
P_ERecA__sfGFP	*E. coli* native recA promoter fused with orf super fold GFP(pMK backbone)	Kan^R^	ColE1
Mutant_α_P_VRecA__sfGFP	P_VRecA_ modified (fully truncated SOS & sigma binding region) promoter fused with orf super fold GFP(pMK backbone)	Kan^R^	ColE1
Mutant_β_P_VRecA__sfGFP	P_VRecA_ modified (fully truncated SOS region) promoter fused with orf super fold GFP(pMK backbone)	Kan^R^	ColE1
Mutant_ɛ_P_VRecA__sfGFP	P_VRecA_ modified (partial truncated (−35) SOS region) promoter fused with orf super fold GFP(pMK backbone)	Kan^R^	ColE1
Mutant_γ_P_VRecA__sfGFP	P_VRecA_ modified (partial truncated (−10) SOS region) promoter fused with orf super fold GFP(pMK backbone)	Kan^R^	ColE1
P_VRecA__sfGFP_ssrA	P_VRecA_ modified (Four AT repeats) promoter fused with orf super fold GFP attached with LVA fast degradation tag(pMK backbone)	Kan^R^	ColE1
P_VRecA_AT___HrpRS_P_hrpL__GFP	P_VRecA_AT_ promoter fused with HrpRS transcriptional amplifiers (pMK backbone)	Kan^R^	p15A
P_VRecA_AT__HrpRS_P_hrpL__sfGFP_TetR	P_VRecA_AT_ promoter fused with HrpRS transcriptional amplifier controlling both *sfGFP* orf and *TetR* orf (pMK backbone)	Kan^R^	ColE1
P_Bad__tdtomato	P_Bad_ promoter fused with orf *tdtomato* fluorescent protein (pGEMT backbone); Source of tdtomato	Carb/Amp^R^	ColE1
P_TetO__tdtomato_HrpV	P_TetO_ promoter fused with orf *HrpV* and orf *tdtomato* (pGEMT backbone)	Carb/Amp^R^	pSC101

We then created a series of mutations and truncations of the P_VRecA_ promoter of wild‐type *V. natriegens*, including a full −35, SOS box and −10 deletion; partial SOS box deletion; full SOS box deletion with intact −35; and −35 and SOS box deletion. It showed that deletion, extension of SOS box, or removal of the −35 sequence completely silenced and disrupted the P_VRecA_ promoter in response to UV‐induced DNA damage (Fig. S4), whilst leaving only −35 site intact increased the background expression level. These results validated our annotation of the SOS box and −35 and −10 sites in the P_VRecA_ promoter, although previous reports proposed different annotations (Bernstein *et al*., 2005; Jiang et al*.,* 2016; Simons *et al*., 2010).

### Rational redesign SOS box of P_VRecA_ promoter

The LexA binding motifs of CTGT and ACAG are reverse complementary (Table 1). The 8‐bp  spacer sequence between the binding sites can twist, allowing LexA dimer to recognize and bind two identical positions of CTGT on *recA* promoter (Zhang *et al*., 2010). Notably, the baseline expression level in the wild‐type promoter of *V. natriegens* was higher than the native *recA* promoter in *E. coli* Nissle 1917 (Fig. 1). Based on the predicted structure of LexA‐DNA complex (Zhang *et al*., 2010), the AT‐rich spacer sequence between CTGT and ACAG in the SOS box would be prone to twist, due to the weaker hydrogen bond in AT pairs, which should enhance LexA‐CTGT binding and repression. Hence, such an AT‐rich spacer may be able to reduce the baseline expression of P_VRecA_ promoter Tables 2 and 3.

**Table 2:** SOS box design in the promoter of *E. coli* and *V. natriegens*.

	SOS sequence 5’ → 3’
P_ERecA_	TTGATACTGTATGAGCATACAGTATAAT
P_VRecA_	TAGACACTGTATGAATCAACAGTATAAT
P_vRecA‐AT‐Repeats_	TAGACACTGTATATATATACAGTATAAT
P_vRecA‐A‐Tandem_	TAGACACTGTAAAAAAAAACAGTATAAT

**Table 3:** The performance of gene circuits in *E. coli* Nissle 1917.

Circuits	Dynamic range (rfu)	LOC (UV/MMC)	Inducer range$ / f.c (fold change)
P_ERecA_‐sfGFP	2292 ± 40–6738 ± 47	1 s	1–10 s
P_VRecA__sfGFP	10 941 ± 767–42 957 ± 1075	1 s	1–10 s
P_VRecA‐AT__sfGFP	3588 ± 127–18 844 ± 501/2193 ± 75–13 284 ± 307	1 s/0.1 µM	1–10 s/0.1 µM–10 µM
P_VRecA___AA_ _sfGFP	53 527 ± 2214–10 068 ± 1965	1 s	1–10 s
P_VRecA___Jungle_Down__sfGFP	2089 ± 74–7088 ± 121	1 s	1–10 s
P_VRecA___Jungle_Up_ _sfGFP	1464 ± 132–6130 ± 162	1 s	1–10 s
P_VRecA___Jungle_Both_ _sfGFP	1271 ± 39–3428 ± 281	1 s	1–10 s
P_VRecA___AT‐_GFP	2765 ± 31–5969 ± 882/3682 ± 55–22771 ± 919	1 s/0.5 µM	1–10 s/0.5–10 µM f.c = 20
P_VRecA__sfGFP + P_E. LexA_‐P_E. RecA_	1268 ± 47 – 7579 ± 124	16 nM	16–750 nM/f.c = 46.87
P_VRecA_‐Amplifier*	1361 ± 186–12 032 ± 257	6.125 nM	6.125 nM–200 nM/f.c = 32.65
P_VRecA_‐Amplifier + Feedback^+^	12 864 ± 261–26 100 ± 1105	6.125 nM	6.215–100 nM/f.c = 16.32

P_VRecA_‐Amplifier*: P_VRecA_AT_ connected with HrpRS transcriptional amplifiers (P_VRecA___AT__HrpRS_P_hrpL__GFP). P_VRecA_‐Amplifier + Feedback^+^: P_VRecA_AT_ connected with HrpRS transcriptional amplifiers linked with P_tetO_‐_tdtomato‐HrpV_ repressor. (P_VRecA___AT__HrpRS_P_hrpL__sfGFP_TetR + P_TetO__tdtomato_HrpV). $: An inducer was UV light (s) or MMC.

Two spacer variants of SOS box with different dynamic responses are shown in Fig. 1 and Table 2. It was found that a spacer region of four AT repeats (P_VRecA‐AT_) (ATATATAT) created a lower baseline expression level, and a more significant fold change than wild‐type promoter and AAAAAAAA (P_VRecA‐AA_) mutant (Fig. 1). Despite a slight decrease in the absolute intensity, compared to the wild‐type P_VRecA_ SOS (TGAATCAA), the baseline expression of P_VRecA‐AT_ was ˜ 2.4 fold lower than that of P_VRecA_. This indicates that P_VRecA‐AT_ allows tight binding of LexA dimer, enhancing repression (Fig. 1). Interestingly, alternation of the spacer to A‐tandem resulted in higher leakiness and less fold change (Fig. 1) despite having similar AT ratio. The spacer with AT repeat might have a high affinity to LexA and lower dissociation constant K_d_. These results suggest that P_VRecA‐AA_ and P_VRecA‐AT_ should have different twist angles and minor grooves in the spacer, affecting LexA binding (Zhang *et al*., 2010). Alternatively, other factors other than LexA binding efficiency may be at play, which is worth further investigation. The performance of variants with different spacers is shown in Table 3.

**Fig. 3:**
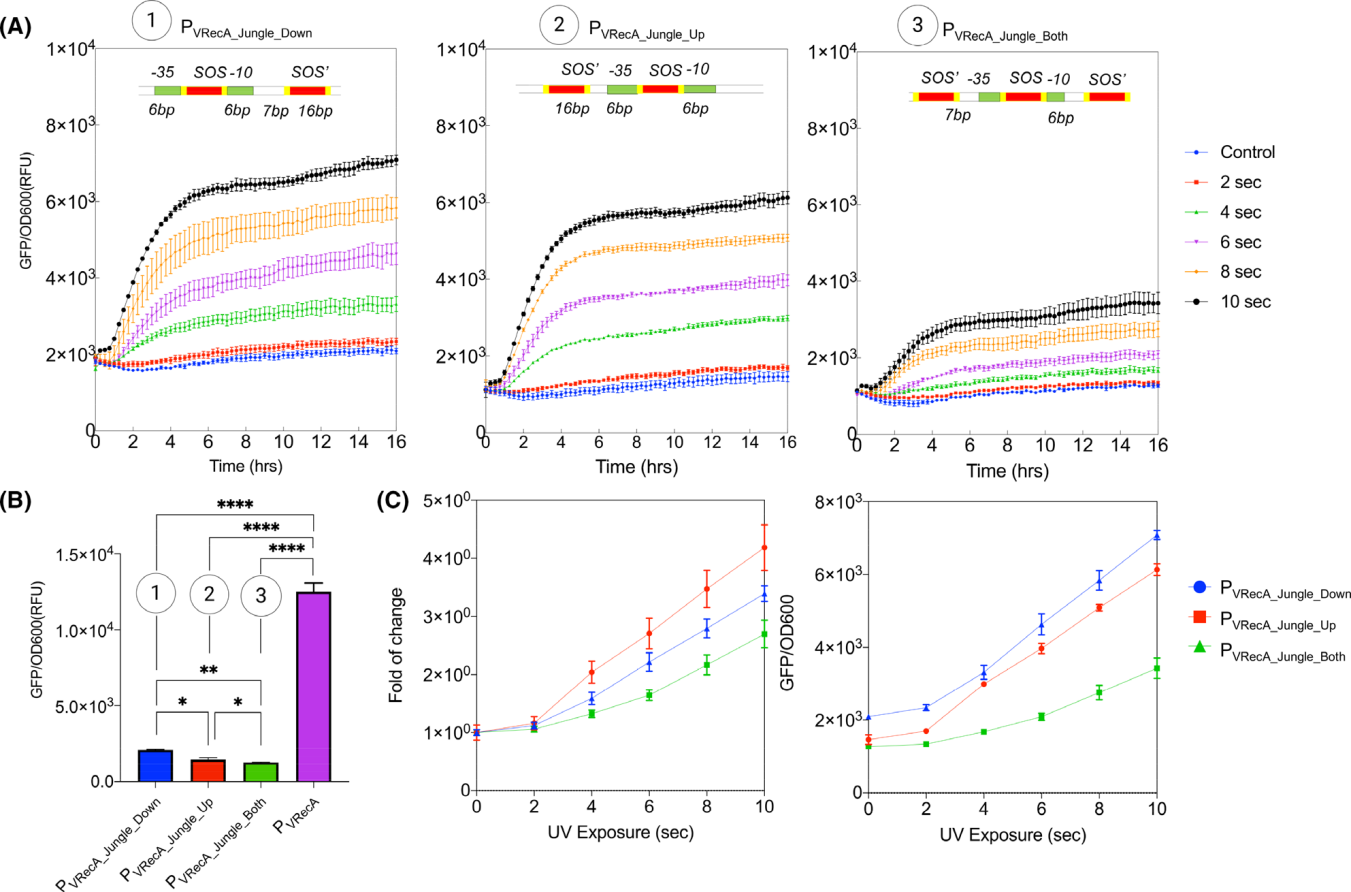
Characterization of chimeric promoters with SOS boxes. Design of each jungle promoter is shown within the graph. Green box indicates the sigma factor binding site −35 and −10. Yellow box indicates the LexA binding site, and red box indicates the SOS box. A. Induction profile of different chimeric promoters under UV exposure. B. A comparison of baseline levels of different chimeric promoters. Pair *t* tests were carried out. *P* < 0.002 **P < 0.02 *P < 0.001 **** (C) Dosage response and induction fold of change profile for each construct under the exposure of UV light. Each experiment has three biological and three measurement replicates.

After obtaining an optimized SOS box (spacer AT repeats), we created three chimeric promoters with multiple LexA binding sites to further suppress the leakiness of P_VRecA‐AT_ promoter. Inspired by the EilR repressor promoter structure in a Jungle Express system (Ruegg *et al*., 2018), an additional SOS box with 5’ complement sequence was inserted downstream of the original SOS sequence to enhance repression. As shown in Fig. 3 and Table 3, this additional SOS box reduced the system background level (10 941 GFP rfu) down ˜ 5.2 fold to 2089   GFP rfu P_VRecA‐AT Jungle_Down_, which could be potentially due to the formation of a hairpin loop between two SOS boxes or recruitment of additional LexA protein at the promoter region (Fig. S5). Upstream insertion of an additional SOS box (P_VRecA‐AT Jungle_Up_) displayed a further reduction of baseline leakiness (to 1464  GFP rfu) with lower noise (Fig. 3A and Table 3). The insertions of both upstream and downstream SOS boxes (P_VRecA‐AT Jungle_Both_) displayed the lowest leakiness (1271  GFP rfu) despite lowering the amplitude of system output (Fig 3A and C and Table 3). All strains harbouring system variants demonstrate similar cell growth (Fig. S6). Overall, these results demonstrated that the P_VRecA‐AT_ promoter could be redesigned to generate different dynamic responses via simple addition of SOS boxes. This modularity could be useful in creating other chimeric promoters with dual‐chemical/spatial control input.

### Signal amplification through transcriptional amplifiers

**Fig. 4:**
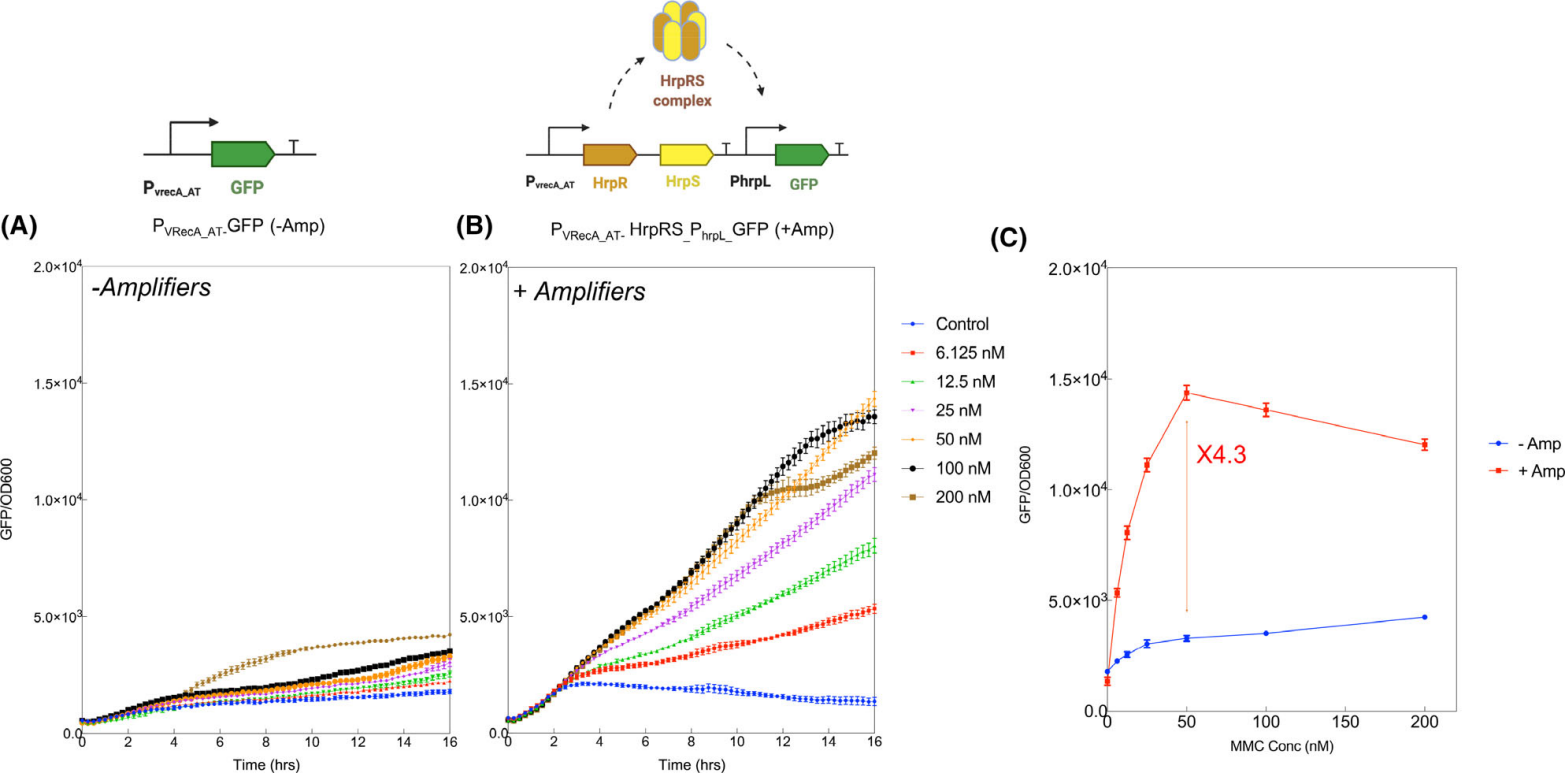
The amplification effect of P_VRecA_ promoter coupled with HrpRS transcriptional amplifier. A. Original P_VRecA_AT_ promoter fused with GFP as an output. B. P_VRecA_AT_ promoter with amplifier. The expression of HrpR and HrpS subunits subsequently activate the PhrpL promoter with a GFP output. Induction kinetics were tested with a concentration gradient of mitomycin C (0;6.125;12.5; 25; 50;100; 200 nM) over 16 h. C. A comparison of P_VRecA_ promoter with and without amplifier in response to different concentrations of mitomycin c (MMC). The maximum amplification can be 4.3‐fold. Each experiment has three biological and three measurement replicates.

To enhance the sensitivity of the DNA damage sensing system, we added an HrpRS‐based transcriptional amplifier (Wang *et al*., 2014) to amplify the output signal of the DNA damage sensing system (P_VRecA‐AT_). As shown in Fig. 4 and S8, the sensitivity can be improved, allowing detection down to 6.125 nM of mitomycin C (MMC), which is by far the most sensitive genotoxic sensor reported (Lemoine *et al*., 2018). The maximum amplification factor for P_VRecA‐AT_ promoter can reach 4.3‐fold when coupled with HrpRS‐based amplifier (Fig. 4) without showing metabolic burden (Fig. S7). However, the introduction of this amplification system led to a significant delay of activation, which the response time extended to ˜ 2.5 h. Compared to the original arsenic amplifiers construct which also displayed a similar time of delay (Wang *et al*., 2014), the delay is likely to be independent of the biosensor target. This delay could be due to the global sigma‐54 regulation of HrpRS system and additional time required for the translation of HrpRS complex in *E*. *coli* Nissle 1917. We have tested a number of nutrient depleting conditions which have previously been shown to reduce response delay in sigma‐54 dependent promoter (Huang *et al*., 2008; Reitzer and Schneider, 2001). We noticed there is a significant difference in response time when different carbon sources were used. Glycerol as carbon source had a shorter delay (2.5 h) compared to glucose as carbon source (4 h) (Fig. S8). Since little difference was observed in the OD, the delay might be related to the global catabolite repression in bacteria (Görke and Stülke, 2008).

### A negative feedback gene circuit to resist fluctuations of environmental conditions

**Fig. 5:**
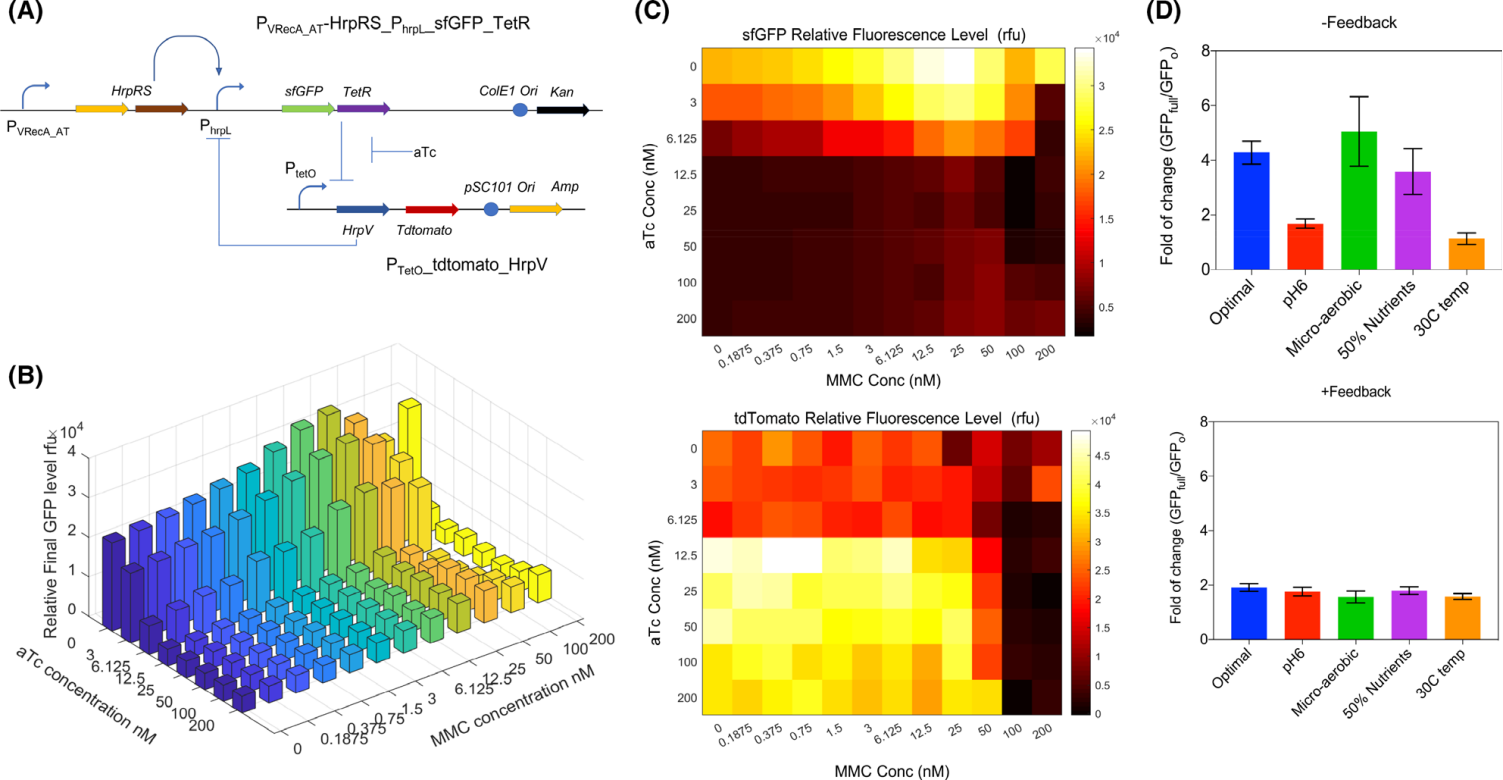
Performance of P_VRecA‐AT_ promoter with amplifier and negative feedback loop. A. Schematic diagram of the genetic operational amplifier with a negative feedback loop. A plasmid consists of the optimized P_VRecA‐AT_ promoter fused with *hrp*RS, sfGFP gene and *tet*R; another plasmids consist of a negative regulator P_hrpL_ and Tdtomatoes reporter. B. Expression profile under the induction of both aTc gradient (0 to 200 nM) and MMC gradient (0 to 200 nM). C. Heatmap of sfGFP and Tdtomatoes expression corresponding to different concentration of inducers. D. Comparison of fold change of P_VRecA‐AT_ amplifier gene circuits with and without the negative feedback loop. Steady state of aTc = 3 nM and MMC = 50 nM at the end of 16 h time course were used to calculate, optimal condition indicates as pH7, oxygen available, 37°C, M9 supplemented with 2%w/v glucose. pH6 was calibrated using HCl titration, micro‐oxygen environment was created using oil seal over each well. 50% nutrient indicated 1% w/v glucose supplemented instead of 2% w/v used in optimal condition. Each experiment has three biological and three measurement replicates.

Although the amplifier constructs demonstrated high sensitivity with minimal leakiness (Fig. 4), it lacks stability and robustness against environmental fluctuation, such as changes in the nutrient richness, pH and temperature. To improve the stability and robustness of our SOS sensing system, we coupled tuneable TetR repression with the transcription amplifier to make negative feedback loop (Fig. 5). This was achieved by adding TetR regulator protein under the control of P_hrpL_ and the counter repressor unit HrpV for HrpRS complex under the control of TetR regulating promoter (P_tetO_). Upon the activation of HrpRS amplifier, the flux of TetR repressor can be tuned by anhydrotetracycline (aTc), thus forming a closed‐loop negative feedback system. To visualize the performance of the negative feedback system, we incorporated a red fluorescence tdTomato downstream of HrpV. When P_tetO_ was activated, we observed an increased expression of tdTomatoes with a decreased expression of sfGFP (Fig. 5C). Upon over induction of P_VRecA‐AT_, the promoter P_hrpL_ was induced to a high level, which also leads to a high expression of TetR. In the absence of aTc, expression of HrpV was repressed, and the overall expression of sfGFP was high. In the presence of aTc, the expression of HrpV caused negative regulation through its interaction with HrpS (Jovanovic *et al*., 2011), and the overall expression of sfGFP decreased. Once aTc reached between 6.25 and 12.5 nM, HrpV was likely to reach saturation, leading to significant repression of overall sfGPP level (Fig. 5). Hence, this negative regulation system can be controlled by the concentration of aTc (<12.5 nM), stabilizing the overall gene expression.

To verify the performance of this negative feedback gene circuit, we subjected it to several conditional changes including temperature, nutrient level, oxygen availability and pH (Fig. S9 and S10). We showed that in the presence of the negative feedback module (under the induction of 3 nM of aTc – thus allowing the expression of HrpV) the P_VRecA‐AT_ sensing circuit exhibited system stability and robustness against environment fluctuations by maintaining the same level of induction fold at steady state (16 h post‐induction) (Fig. 5D, S9, and S10). Despite the decrease in the relative fold of change and narrow operational range caused by HrpV, which could be solved through mining lower affinity mutant. The increased robustness indicates the potential of our P_VRecA‐AT_ sensing system with additional feedback module for *in situ* applications, where exposure to the real‐world environment or gut environments can make it challenging for synthetic circuits to perform as predicted.

Overall, we described an ultrasensitive DNA damage sensor equipped with a functional genetic operational amplifier that combines a chimeric promoter as the noise canceller, a HrpRS‐based construct as the signal amplifier and a P_tetO_‐negative feedback system.

### Spatial control and adaptation of SOS gene circuit in response to UV light

We next demonstrated the robust and sensitive response of the P_VRecA_ promoter, demonstrating UV inducible gene circuits through a spatial control. As a proof‐of‐concept, *E*. *coli* Nissle *1917* with P_VRecA‐AT_ system formed colonies on LB plates, which were activated by a UV light to express sfGFP (Fig. S11). The fluorescence of sfGFP‐expressing colonies was imaged after UV exposure for 10 and 20 s, respectively, following 4 h incubation at 37°C. The sfGFP expression under chimeric P_VRecA‐AT Jungle_Down_ promoter displayed a lower baseline but less sensitivity, compared to that under P_VRecA‐AT_ (Fig. S11A), the results from solid biofilm are consistent with the experiments done in bacterial liquid culture condition shown in Figs 1 and 2. Interestingly, a ring‐like structure was observed (Fig. S11), and this could suggest SOS system being induced to express sfGFP in newly grown cells more readily than the growth stalling old cell in the centre.

**Fig. 6:**
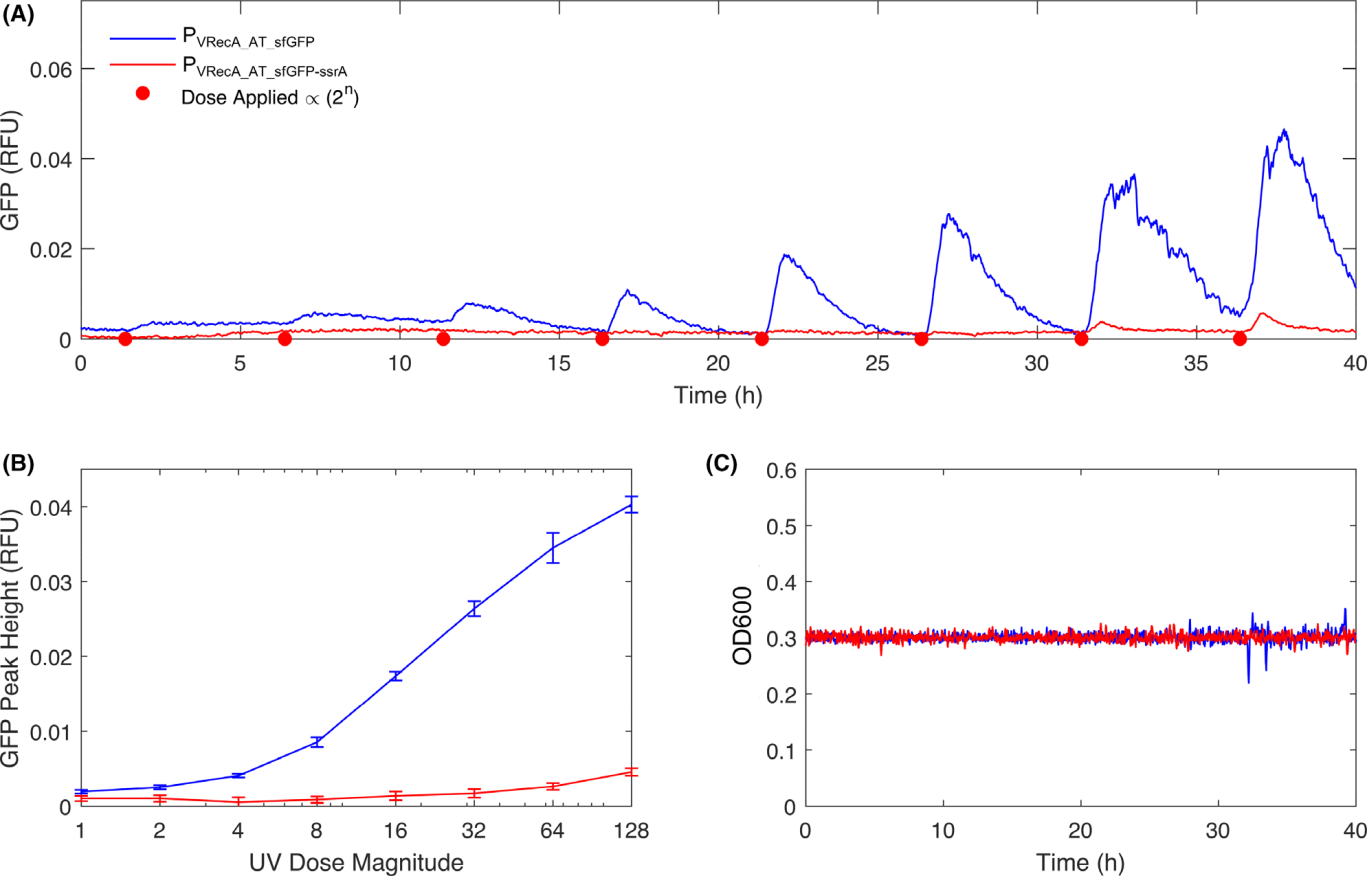
Adaptation of *E. coli* Nissle 1917 with P_VRecA‐AT_ –sfGFP and P_VRecA‐AT_ – sfGFP‐ssrA in turbidostat over 40 h with interval UV exposure. A. Dynamics of sfGFP expression under periodic UV exposure. Every 5 h is a cycle. The first UV dose was 10 mW 280 nm for 2.4 s and the UV doses doubled in the next cycle. Small fluorescence expression was observed for P_VRecA‐AT_ – sfGFP‐ssrA as a rapid degradation of fluorescent protein with ssrA tag. B. Relationship between applied UV dose and fluorescence peak height. Error bars are the standard deviation of the ten reads in fluorescence taken in the 10 min window entered on each peak. Each experiment has four replicates. C. OD was maintained constant in the turbidostat during the experiment.

To investigate temporal dynamics and adaptation of the DNA damage sensor, we tested it using a Chi. Bio turbidostat platform(Steel *et al*., 2020), which can maintain a constant culture OD while measuring fluorescence and inducing DNA damage with 280 nm UV radiation. Two variants were tested (P_VRecA‐AT_ with either sfGFP or sfGFP attached with ssrA fast degradation tag), with each subjected to a UV dosing regime in which radiation dose intensity was doubled every 5 h (Fig. 6). For the sfGFP variant, we observed a log‐linear relationship between UV dose and sfGFP expression, with the system demonstrating a reliable response over a 128‐fold increase in induction level (Fig. 6). In each cycle, a return‐to‐baseline took approximately 5 h which we hypothesized was due to the time necessary for GFP dilution during growth. The sfGFP‐ssrA (LVA‐ BBa_K1399001) construct (in which the fluorescent marker is degraded on a faster timescale than cellular growth) supported this hypothesis, demonstrating that our optimized P_VRecA‐AT_ reporter system can be used to generate short (˜1 h) pulses of gene expression in response to UV induction.

We next analysed the sfGFP kinetics using a microplate reader to assess circuit behaviour in a different physiological context. In Fig. S12, using 2 s of UV exposure, sfGFP‐ssrA is able to generate a short GFP pulse behaviour with fast degradation, 2.5 h are required for the emerging peak to return to ground states. In comparison, the original sfGFP showed similar pulse behaviour but did not return to zero within 16 h. Collectively, the P_VRecA‐AT_ promoter induction in *E. coli* Nissle 1917 can be triggered in < 2h and the duration of the downstream expression is between 2.5 and 5 h and the strength of SOS expression can be adapted to reach significantly higher levels after intermittent stimulation and continuous cultivation.

### Application of SOS biosensors to gut toxins and antibiotics

**Fig. 7:**
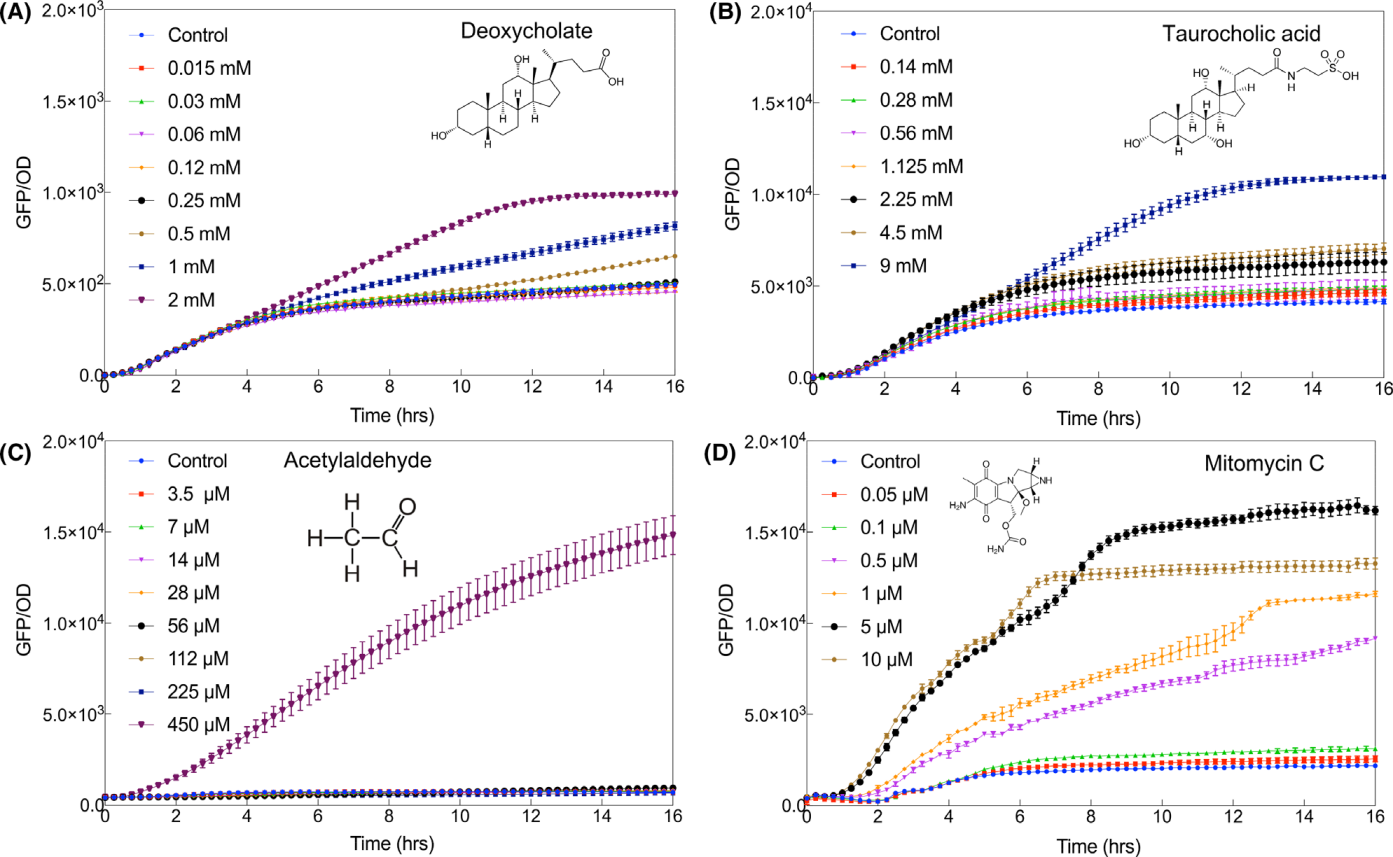
Application of P_VRecA‐AT_ –sfGFP for the detection of bile salt derived carcinogens and other DNA damaging compounds: (A) deoxycholate, (B) taurocholic acid, (C) acetaldehyde and (D) mitomycin C. Each experiment has three biological and three measurement replicates.

We applied the optimized SOS sensing system P_VRecA‐AT_ to different DNA damaging compounds. One application is the detection of genotoxic biomarkers that are associated with colon cancer, which includes bile salt derivatives: deoxycholate, taurocholic acid (Nguyen *et al*., 2018; Sánchez, 2018) and other DNA damaging compounds related to diet such as acetaldehyde (Seitz and Becker, 2007). As shown in Fig. 7, the DNA damaging sensing circuit of P_VRecA‐AT_ can detect genotoxic effects of 0.5 mM deoxycholate, which is a common secondary bile salt derivate metabolized by gut microbiome (Bernstein *et al*., 2011). The normal gut concentration of deoxycholate ranges from 450 ˜ 700 µM (Hamilton *et al*., 2007), high concentrations of deoxycholate have been correlated with colon cancer and could induce DNA breakage (Bernstein *et al*., 2011). Furthermore, the sensing system demonstrated a detection threshold of 9 mM taurocholic acid, which is within the range of estimated local concentration in the gut (Bernstein *et al*., 2005; Ridlon *et al*., 2016). This is the first report to directly quantify the genotoxicity of taurine conjugated bile salt. We proved that acetaldehyde (Na and Lee, 2017) (> 450 µM) is also detectable using P_VRecA‐AT_ sensing system (Fig. 7C). It has been shown that bile salts are able to damage membrane and require bacterial membrane transport system to get into cells (Merritt and Donaldson, 2009; Begley *et al*., 2005). It might explain the delay of response in deoxycholate, taurocholic acid (Fig. 7A and B). Overall, by using the redesigned gene circuit of SOS sensing system carried by a probiotic‐based *E. coli* Nissle 1917, we were able to detect carcinogenic or genotoxic compounds. Our approach has clear merits compared to existing methods, which often rely on animal testing (Gad, 2014) and costly mammalian cell‐based assays such as AME test (Guy, 2014) or UMU assay (Hamer *et al*., 2000).

To compare the strength of genotoxic effect of deoxycholate, taurocholic acid and acetaldehyde, we also examined the induction of mitomycin C, which is a widely used chemotherapeutic drug that causes cancer‐specific DNA cross‐link during cell replication (DNA Repair and Human Disease, 2006; Paz *et al*., 2012; Händel *et al*., 2016). A dosage guideline for mitomycin C was provided in Table S3, where the concentration can be varied between 12 µM and 24 µM depending on the target tumours. Within the testing concentrations of these carcinogenic compounds, there is no impact on the cell growth of *E. coli* Nissle 1917 with sensing plasmid P_VRecA‐AT‐sfGFP_ (Fig. S13) except the case when deoxycholate is used as an inducer, where deoxycholate could serve as a carbon source for *E. coli* Nissle 1917 (Nzakizwanayo *et al*., 2015).

To further extend the application spectrum in food security, we have also tested several fluoroquinolones derived antibiotics (levofloxacin, enrofloxacin and ciprofloxacin) which represent some of the most commonly prescribed antibiotics for bacterial infections (Marchant, 2018). Despite their efficacy and high penetrability, these antibiotics show severe carcinogenic effects on mammalian cells, including irreversible nerve damages and tendon rupture (Herbold *et al*., 2001; Anchordoquy *et al*., 2019). Although the prescription of fluoroquinolones to human patients has been warned against by the FDA (WHO, 1998), fluoroquinolones are still commonly used in animal feedstock which poses an enormous risk in food security.

**Fig. 8:**
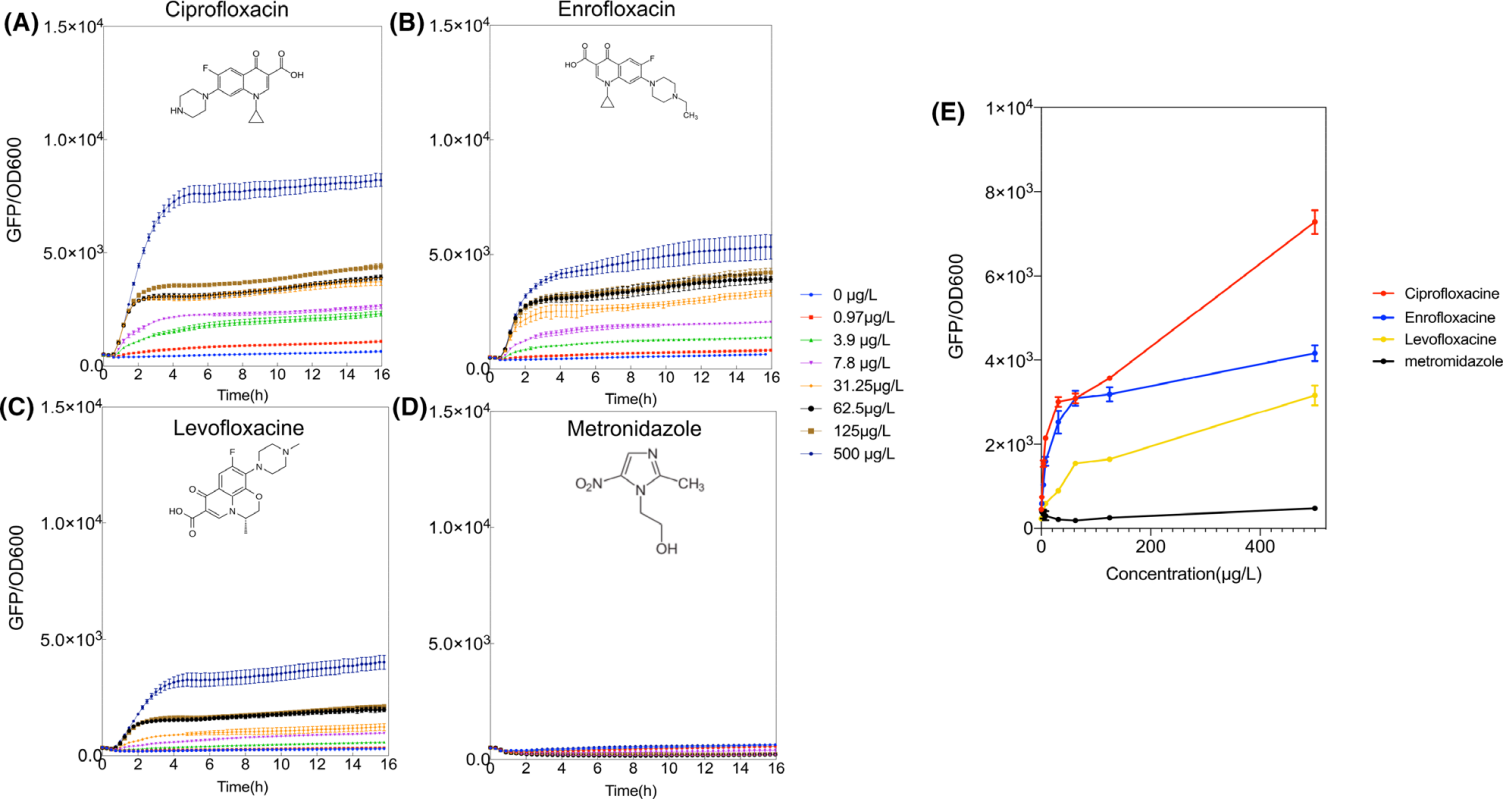
Application of P_VRecA‐AT_ –sfGFP for the detection of the genotoxicity effect of some antibiotics: (A) ciprofloxacin, (B) enrofloxacin, (C) levofloxacin and (D) metronidazole. (E) Dosage responses of different antibiotics. Each experiment has three biological and three measurement replicates.

Existing methods to detect the presence of fluoroquinolone commonly involved micellar electrokinetic chromatography (MEKC) (Prutthiwanasan and Suntornsuk, 2018), high‐performance liquid chromatography (HPLC) (He and Blaney, 2015) and mass spectrometry (MS) (Zhang *et al*., 2018). These instruments lack portability and are costly in use. In contrast, our optimized P_VRecA‐AT_ sensing system could detect fluoroquinolone down to 0.97 µg l^‐1^, which is far below the usage limit of fluoroquinolones for animal feedstock (1.5–15 mg l^‐1^) (Subbiah *et al*., 2016) (Fig. 8 and Fig. S14). Similarly, metronidazole is another type of antibiotic that is also heavily used in animal feedstock and veterinary. However, the P_VRecA‐AT_ sensing system showed no response, which might be due to the fact that the cytotoxicity of metronidazole only arises following reduction by the liver (Dingsdag and Hunter, 2018). The OD graph (Fig. S14) confirms our suggestion that metronidazole does not affect cells growth, but all three other antibiotics were found to stall cell growth at a concentration higher than 125 µg l^‐1^.

## Discussion

In this paper, we analysed the *recA* promoter in *V. natriegens*, identifying the sigma binding sites (−35 and −10) and SOS box in order to develop an ultrasensitive, rapid and robust DNA damage sensor. Although there is no direct evidence to support a correlation between SOS response time and bacteria growth rate, several studies reported links between SOS and DNA replication (Burby and Simmons, 2020), modulating growth pattern (Kreuzer, 2013) and growth fitness (Erill *et al*., 2007). We found that recA promoter P_VRecA_ from the fast‐growing *V. natriegens* has higher sensitivity and broader dynamic range than that of *E. coli* P_ERecA_. We then engineered the P_VRecA_ promoter through a rational and systematic approach to reduce its background noise and enhance its sensitivity. Compared to conventional *ad hoc* method, our approach provided an example of bottom‐up engineering. Based on the structure of the LexA protein and its quantified interaction with the promoter (Kozuch et al*.,* 2020; Culyba *et al*., 2018), we identified the precise location of the SOS box and tuned and optimized the SOS box to create a P_VRecA‐AT_ promoter with low leakiness and broader dynamic response. This engineering process also showed that the SOS box is a modifiable biological part that could be fitted into the synthetic gene circuit design process (Ceroni *et al*., 2010).

An ideal synthetic gene circuit should have the capacity to withstand stochastic and environmental perturbations while maintaining a high signal‐to‐noise ratio. To improve our circuit’s robustness, we employed a previously reported Jungle Express system (Ruegg *et al*., 2018) which could incorporate our optimal SOS sequence. Meanwhile, by using a HrpRS‐based genetic amplifier system, we managed to increase the detection sensitivity and signal amplitude significantly with a minor increase of the system response time. To improve the stability of our optimized P_VRecA_ sensing circuit, we incorporated a negative feedback system to achieve a more predictable operation upon changes in the environment. Together, we proved that our DNA damage sensing circuit confers similar robustness in response to changes in temperature, nutrient level, oxygen availability and pH. In essence, we engineered a functional SOS responsive operational amplifier by combining a noise canceller, a signal amplifier and a negative feedback system within a synthetic gene circuit. The merit of this DNA damage sensing system is not merely the potential to perform under real‐world stochastic environments such as the human gut, but also the engineering room for creating user defined dynamics and potential chimeric promoter, these features allow such circuits to be used in a plug‐and‐play fashion.

We demonstrated that our DNA damage sensing system could be used as an optogenetic actuator through the induction of UV light. In the experiment where we exposed the macro‐colonies transformed with our DNA damage sensing system with UV light, we showed that a 10‐s temporal difference could alter the phenotype of the macro‐colonies. As a proof‐of‐concept, our sensing system can be extended as a valuable tool to study signalling dynamics, generation of persisters and acquisition of antibiotic resistance within a biofilm (Goldman and Travisano, 2011; Yin *et al*., 2019). For instance, using our system to monitor the UV damage and its corresponding biofilm formation. Despite the system activation being different to canonical optogenetic systems such as the Cph8/OmpR system and BphP1(Liu *et al*., 2018) which commonly involve a light‐sensing domain, we proved that our ultrasensitive DNA damage sensing system is adaptable and functional upon UV induction without altering the growth of the bacterial host. Although the UV dose of P_VRecA___AT‐sfGFP_ gene circuit was increased 128‐fold in 40 h, no mutation on the plasmid P_VRecA‐AT‐sfGFP_ and P_VRecA‐AT‐sfGFP‐ssrA_ was found during the entire course of the experiment (Fig. S15), according to DNA sequencing. It has been reported that bacterial gene regulation systems can be adapted in response to the environment without DNA mutation (Ibarra *et al*., 2002). This further verifies the potential of our sensing system as a programmable medicinal tool, strains with preloaded P_VRecA‐AT‐sfGFP_ can be trained to extend its operational dynamic as ‘field‐ready’ strains with different characteristics. Another merit of using irradiation over molecular triggers as it is not restricted by diffusion limitations. Nevertheless, since our sensing system uses transcriptional machinery to detect UV light, the operational timescale could be longer than translational sensors such as a riboswitch (Chang *et al*., 2018; Wang *et al*., 2020) or two components system with membrane receptors (Chang *et al*., 2018).

Finally, we demonstrated the potential of this optimized P_VRecA‐AT_ promoter for its real‐world application. The key application of this sensing system is to detect carcinogens, such as genotoxic compounds remaining in agricultural or dairy products, and secondary metabolites generated by the human microbiome. Our DNA damage sensing system can potentially be further developed into a Point of Care detection device or used as a programmable vehicle for living medicine. Compared to currently existing tools for carcinogen detection such as the Comet Assay (Fairbairn *et al*., 1995) and the Ames (Bacterial reverse mutation) tests (Hamel et al*.,* 2016), our sensing system offers a cost‐effective, fast and easy‐to‐operate solution for such measurements. (Fairbairn *et al*., 1995).

## Experimental procedures

Each experiment for the performance of gene circuits has at least three biological replicates and three measurement replicates.

### Bacterial strains, media and chemicals

*Escherichia coli* DH5α was routinely used for molecular cloning and plasmid maintenance. *E. coli* BL21(DE3) was used for the initial screening and characterization of the genotoxic biosensor. Final optimized constructs were tested in probiotics strain EcN (*E*. *coli* Nissle 1917), obtained from ardeypharm GmbH (Herdecke, Germany). Enrofloxacin, metronidazole and levofloxacin were acquired from Adamas‐beta, Shanghai, China. Ciprofloxacin was acquired from TCI, Shanghai, China. Unless otherwise stated, all chemicals used were from Sigma‐Aldrich (Dorset, UK).

For routine cell growth, bacteria were inoculated from a colony and grow in Luria–Bertani (LB) broth with corresponding antibiotics kanamycin (60 μg ml^‐1^); ampicillin (100 μg ml^‐1^) and incubated at 37°C, 250 rpm for 16 h. For double‐plasmid transformation, equal molar of the two plasmids was electroporated into competent cells at the same time, followed by using an LB cocktail with kanamycin (50 μg ml^‐1^) and ampicillin (100 μg ml^‐1^) for inoculation and bacteria culturing over 16hrs.

For the expression experiments, M9 medium was made from M9 5X minimal salts (Sigma‐Aldrich) supplemented with 0.4% w/v glucose and corresponding antibiotics. SOC medium was made according to CSH protocol (cshprotocols.cshlp.org).

### Gene circuits design and plasmid construction

All molecular procedures were carried out with enzymes obtained from New England Biolabs (NEB), and all primers were synthesized by Sigma‐Aldrich. For initial screening and annotation of the promoter region, the randomized promoter library was constructed using randomized oligos (NNNNNNNNACAGTATAATAACTTTCATTGCTGAGCG&NNNNNNNNACAGTGTCTATACCTGTATAGAAAAACTTTAGC) using the template (P_V. RecA__sfGFP), followed by Q5 SDM protocol. A mixture of sfGFP plasmids with the randomized promoter (SOS box) was then transformed in *E. coli* BL21 competent cells. Colonies with different degree of background leakiness were screened using VersaDoc® imaging system by Bio‐Rad. Kinetics reads were followed by an overnight bacterial culture of the colony of interests. For each transformed mutant, plasmids were purified from QIAprep Spin Miniprep Kit and the sequence of promoter and SOS box region was determined via ‘TubeSeq Service’ provided by Eurofins Genomics. The quality of DNA was routinely checked with agarose gel electrophoresis (1% w/v) and NanoDrop (260/280 ≈1.8 and 260/230 ratio ≈2). Plasmids used in this study are shown in Table S1, and detailed plasmids map are provided in Table S4. For standard cloning procedure, gene fragments were synthesized via GeneArt with the backbone of pMK( Kan^R^ ColE1 ori), RBS (B0032) and sfGFP used in this study are standard parts obtained from Registry of Standard Biological Parts. (www.partsregistry.org). The *v.recA* binding site and promoter sequence were determined by analysing the sequences of *v.recA* operon (the GenBank accession No. CP016351.1). Similarly, control *E.coli K12 recA* operon was retrieved from GenBank: No. CP025268.1and for *E*. *coli* Nissle *1917* GenBank No. CP007799.1.

### Bacterial transformation

To make chemically competent *E. coli* Nissle 1917 cells, bacteria were first grown to exponential phase by inoculating overnight culture in fresh LB broth at 1:100 dilution and incubated at 37°C, 200 rpm for 2 h or until OD600 of 0.3 or 0.4 was reached. Cell culture at exponential phase was chilled on ice for 30 min followed by centrifugation at 3500 rpm, 4°C for 10 min. The supernatant was removed, and the cells were washed twice with ice‐cold CaCl_2_ (0.1 M) and resuspended in CaCl_2_ (0.1 M) and glycerol (20% w/v).

For transformation, competent cells (100 μl) were added with a plasmid (100 ng) and mixed by flicking. The cell suspension was chilled on ice for 30 min and transferred to a 42°C water bath for 45 s, followed by incubation on ice for 2 min. SOC media (900 μl) was added and mixed, and the cell suspension was incubated at 37°C, 200 rpm for an hour before plating on kanamycin (60 μg ml^‐1^) agar plate.

### DNA damage induction by mitomycin C, UV, cancer biomarkers, genotoxic antibiotics

Evaluation of the genotoxicity biosensor was done in Corning Black 96‐well plate (flat, clear bottom): Overnight bacteria culture of 5 μl was added into 190 μl medium containing M9 minimal media (1x), glucose (0.4 %w/v) and kanamycin (60 μg ml^‐1^), and 5 μl mitomycin C at concentrations ranging from 0.05 to 250 μM. For UV induction, 5 μl overnight bacteria culture was added into 195 μl medium containing M9 minimal media (1x), glucose (0.4 %w/v) and kanamycin (60 μg ml^‐1^), followed by 6W, 302 nm UV exposure from 0 to 10 s using Benchtop UVP Transilluminators (Thermo Scientific UK). The plate was incubated in a BioTek plate reader at 37°C for 16 h with OD600 and luminescence or GFP reading being measured every 15 min. All experiments were carried out in three biological repeats with three technical replicates each time. Error bars represent the mean and standard deviation, respectively. The processed data were plotted with OriginPro Version 9.1 and analysed using in the built statistical function of prism 8. Unpaired *t*‐test was carried out for comparing baseline within each variant. Significantly different *P* < 0.05 was used.

### UV induction in turbidostats

For turbidostat experiments, we employed the Chi. Bio experimental platform^57^ with culture volumes of 20ml. M9 minimal media (1x), 5 mM MgSO_4_•7H_2_O, 0.01%w/v thiamine, glucose (0.4 %w/v) and kanamycin (60 μg ml^‐1^) were used, and experiments were run until substantial biofilms were observed to form in the culture chamber. Fluorescence measurements of sfGFP were performed each minute using excitation/measurements of 457/510 nm, respectively. Cultures were grown to an OD of 0.3 and maintained via Turbidostat functionality for ˜ 5 h prior to the first UV dose in each experiment. This first UV dose (i.e. 1X) was equal to activating a 10 mW (optical power) 280 nm LED for 2.4 s. In each subsequent dose, the duration of LED activation was doubled. Peak height in Fig. 6 is defined as the difference between the maximum GFP reading in the 5 h following a UV dose and the minimum UV reading in the preceding 5 h (e.g. since the previous UV dose). For the sfGFP‐ssrA experiment, fluorescence peaks were indistinguishable from measurement noise below UV doses of 16X.

### Macro‐colony growth and UV induction

For biofilm growth, 100 μl of transformed overnight culture was aliquoted onto LB agar with 50 μg ml^‐1^ kanamycin supplemented. This was allowed to grow into a circular macro‐colony (˜2cm diameter) upon 16 h incubation at 37°C. Subsequently, the fully developed biofilm was exposed to 302nm UV for 10 and 20 s. Fluorescent reads were taken 4 h after the exposure using Molecular Imager VersaDoc imaging system by Bio‐Rad (Kidlington, UK). Light micrographs were subsequently analysed using ImageJ version 1.50b, with fluorescent maxima automatically counted after consistent thresholding.

## Funding Information

WEH acknowledges support from EPSRC (EP/M002403/1 and EP/N009746/1).

## Conflict of interest

The authors claim no conflict of interest.
