# Supplementary material for: Redesign of ultrasensitive and robust RecA gene circuit to sense DNA damage
Source: Microb Biotechnol. 2021 Mar 4;14(6):2481–96. doi: 10.1111/1751-7915.13767 (PMC8601168; doi:10.1111/1751-7915.13767)
Supplement: Supplementary file 1 [file MBT2-14-2481-s002.pdf]

Supplementary figures

Redesign of ultra-sensitive and robust RecA gene circuit to sense DNA damage

Jack Xiaoyu Chen[a], Boon Lim[a], Harrison Steel[a], Yizhi Song[a] ,Mengmeng Ji[b] and Wei E. Huang[a*]

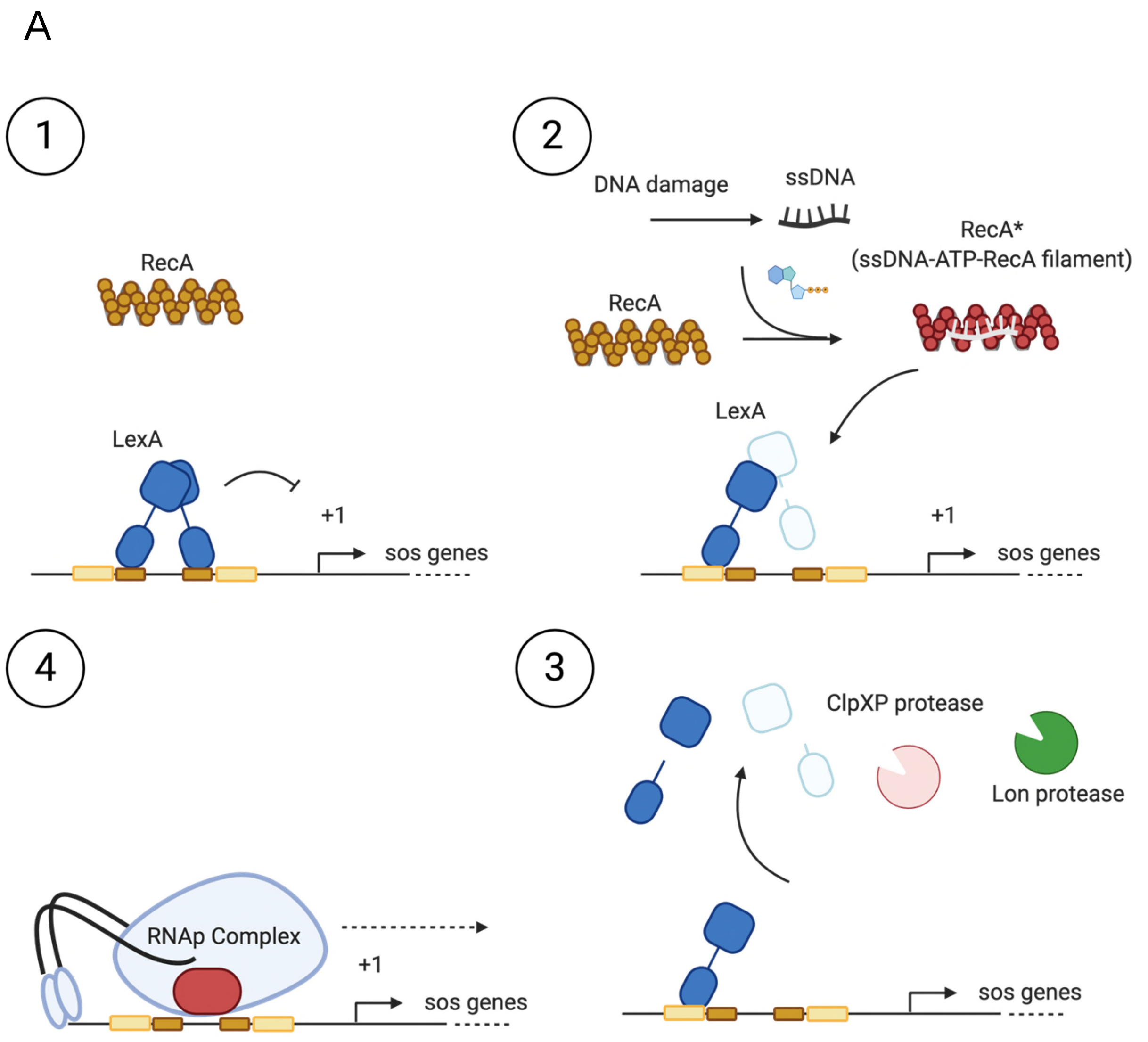

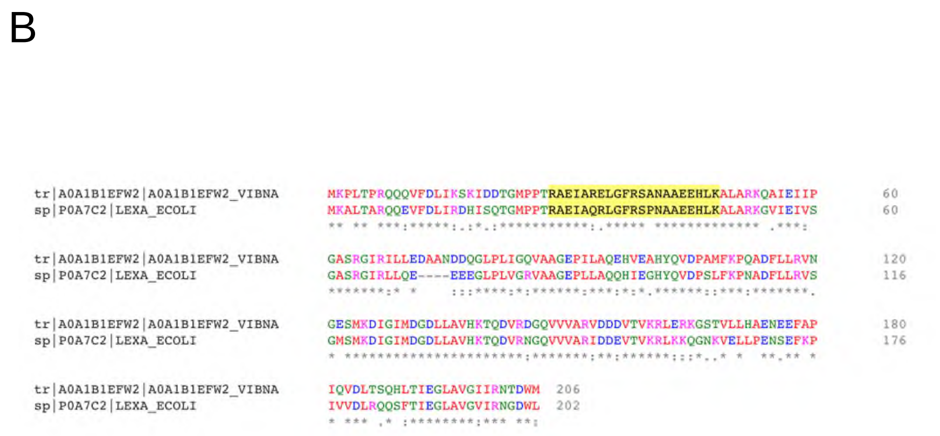

Figure S1. A) Schematic diagram of SOS DNA repair mechanism. In its native state, SOS machinery is dormant and repressed by LexA while low level of RecA is stored inside cell in its inactive form; upon DNA damage, single-stranded DNA will activate RecA ((ssDNA-ATP-RecA filament) which subsequently trigger the self-cleavage of the LexA dimer and furhter degradation of Lon protease and ClpXP, fully release it from the SOS binding sites, resulting the activation of 40 different DNA repair genes. B) LexA protein sequence alignment between *V. Natrigene* and *E.coli* k12, a similarity score of 72% and amino acid residue responsible for DNA binding region is highlighted in yellow.

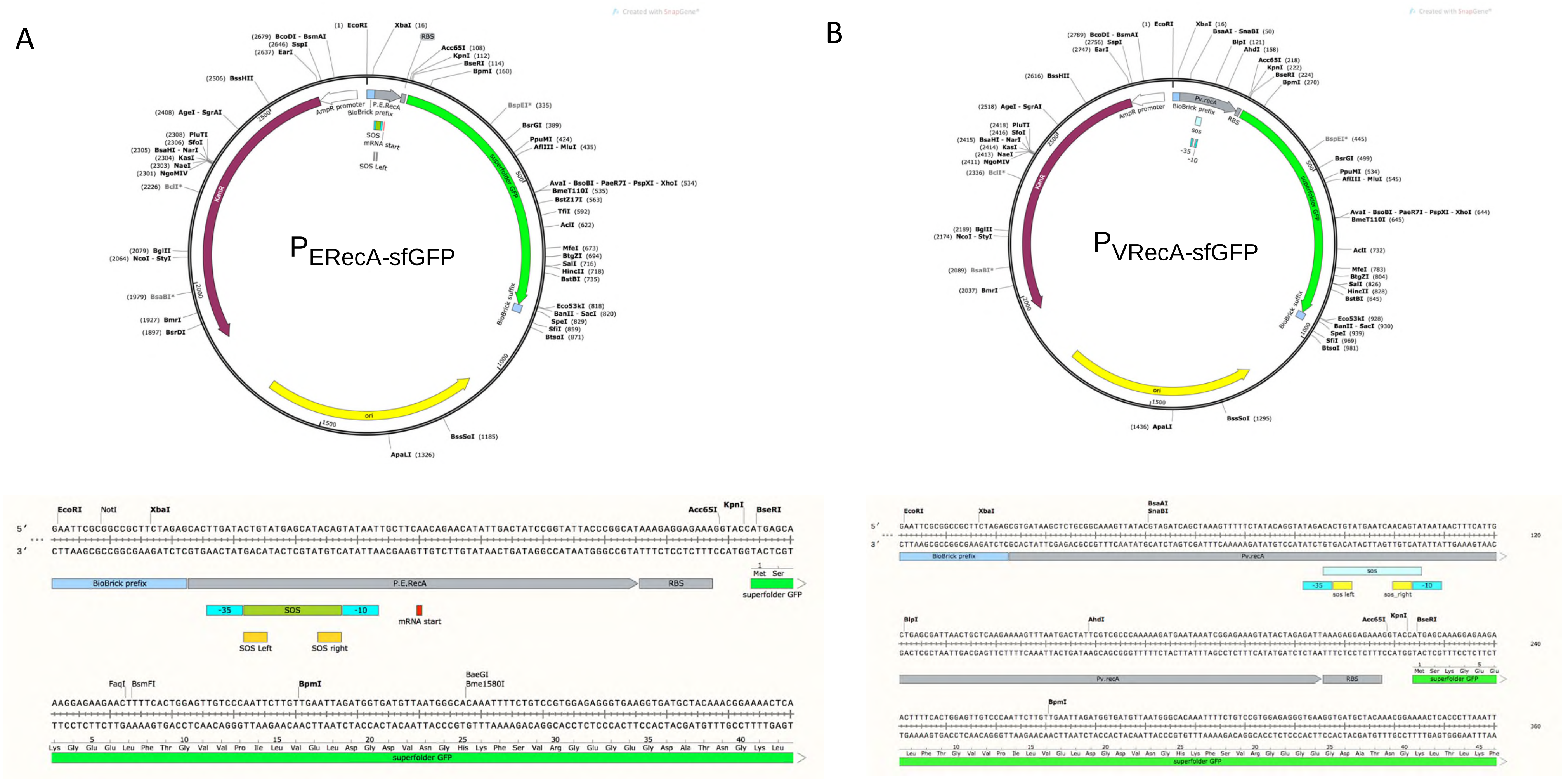

Figure S2. Plasmids map for E.RecA and V.RecA_wt_sfGFP. SOS binding region of A) E.RecA and B) V.RecA were annotated with LexA binding site (Yellow), Sigma factor binding site (Blue) and potential mRNA start site (Red).

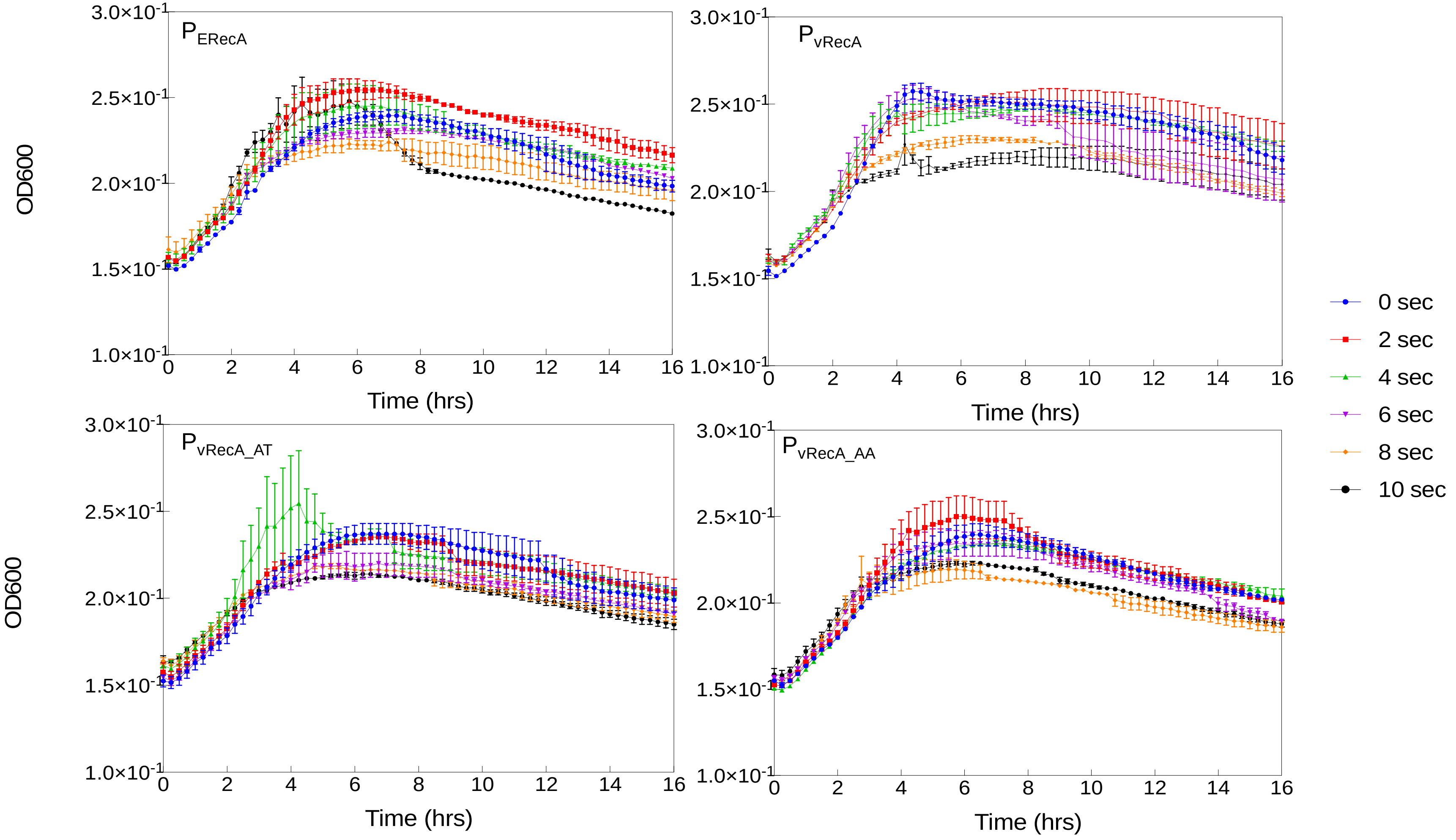

Figure S3. OD growth curve for all three V.RecA promoters with different SOS binding box and E.RecA promoter tested with (0 ~ 10 sec) 302nm UV exposure. The promoters were transformed into *E. coli Nissle 1917* supplemented in M9 medium with 0.4% glucose and Kanamycin 60 µg/ml. N=3.

A

	DNA SOS Truncated sequence 5' → 3'
P_{VRecA}	TAGACACTGTATGAGCATACAGTATAAT
α-P_{VRecA}	TAGA------20bp Deletion---------TAAT
β-P_{VRecA}	TAGACACTGTAAA-5bp-ACAGTATAAT
ε- P_{VRecA}	TAGACA---16bp Deletion-----TATAAT
γ-P_{VRecA}	----- 18bp deletion------ACAGTATAAT

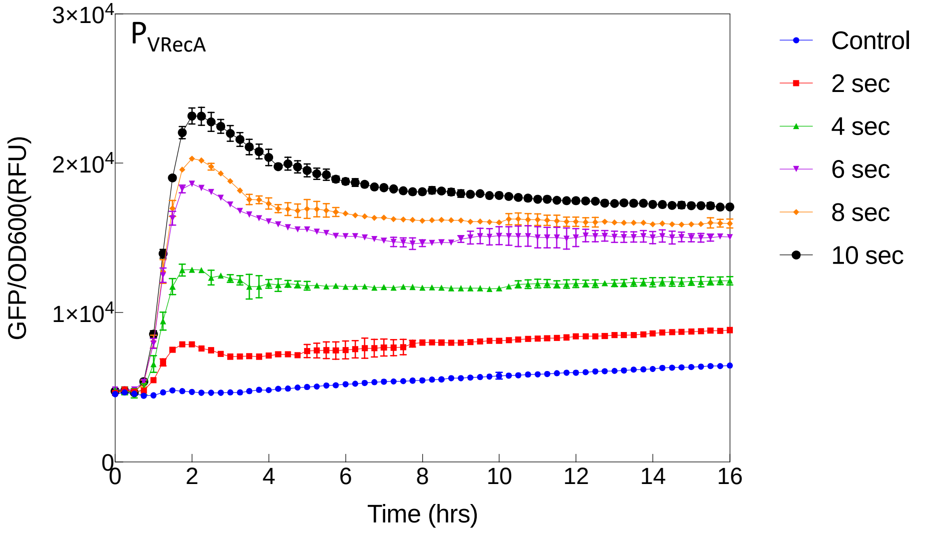

B

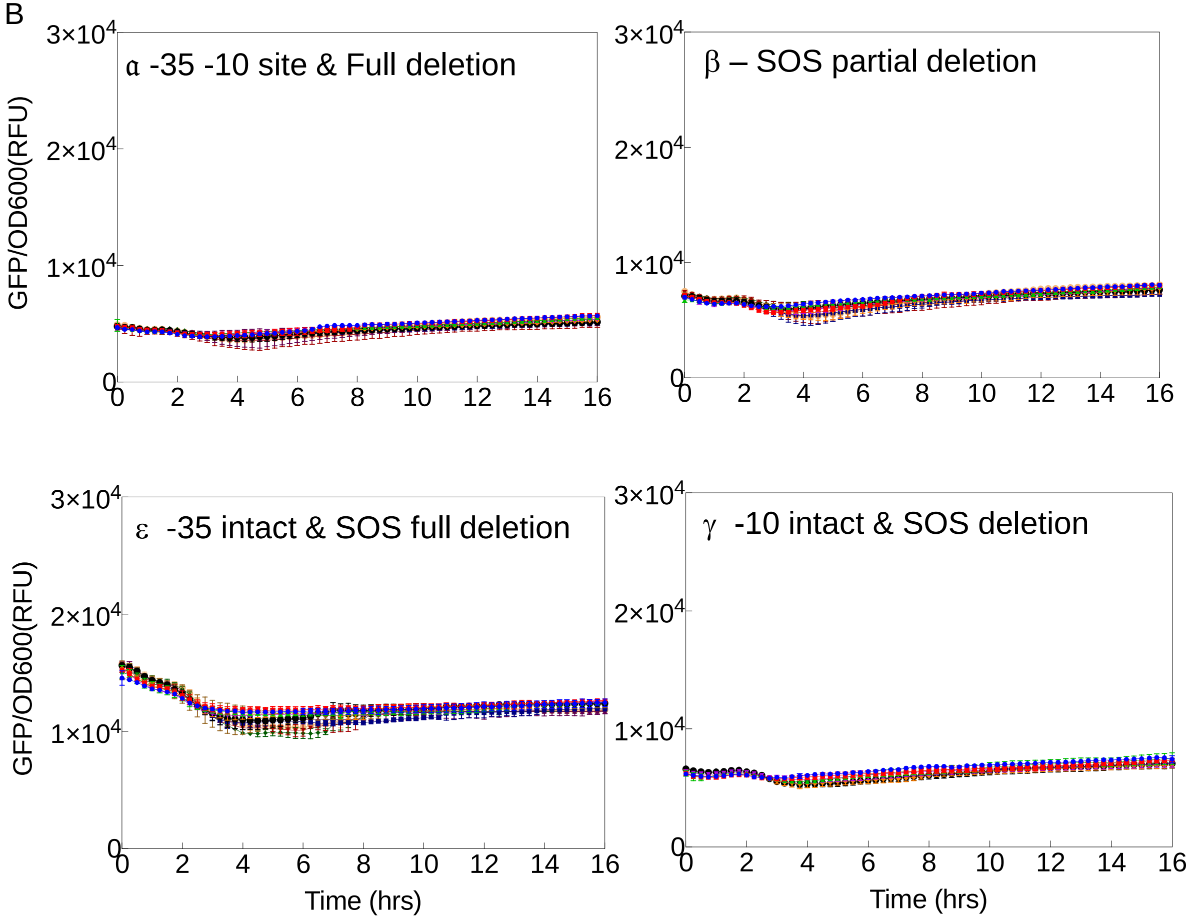

Figure S4 Performance of V.RecA promoter variants induced by 302 nm UV. A) Table indicates different variants of V.RecA promoter. Yellow box indicates the sigma binding site and Red box indicates the SOS box. B) Induction profile over 16hrs. Control (Wild type V.RecA promoter) is shown on the left. N=3.

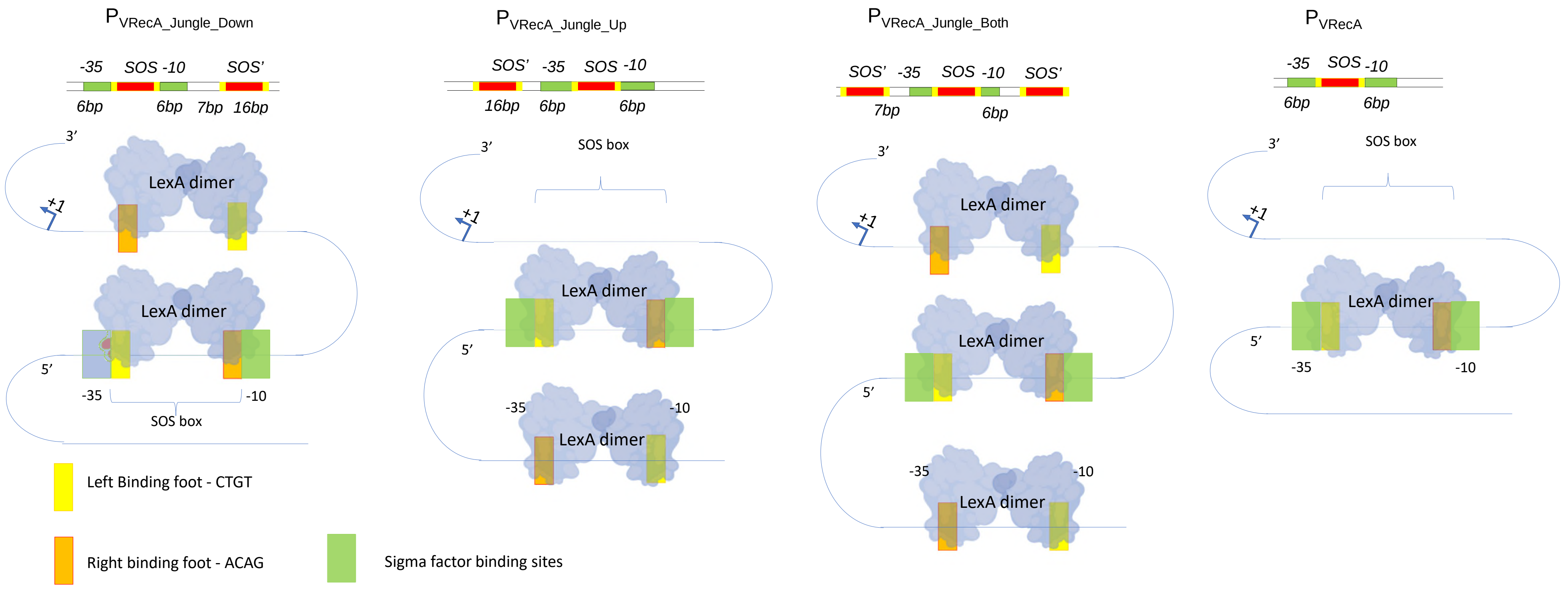

Promoter Name	Sequence 5'→3'
P_{VRecA}	CGTGATAAGCTCTGCGGCAAAGTTATACGTAGATCAGCTAAAGTTTTCTATACAGGTATAGACA**CTGTATATATATACAG**TATAATAACTTTCATTGCTGAGCGATTAACTGCTCAAGAAAAGTTTAATGACTATTCGTCGCCCAAAAAGATGAATAAATCGGAGAAAGTATACTAGAGATT
Jungle Up	CGTGATAAGCTCTGCGGCAAAGTTATACGTAGATCA**CTGTATATATATACAG**ACAGGTATAGACA**CTGTATATATATACAG**ATAATAACTTTCATTGCTGAGCGATTAACTGCTCAAGAAAAGTTTAATGACTATTCGTCGCCCAAAAAGATGAATAAATCGGAGAAAGTATACTAGAGATT
Jungle Down	CGTGATAAGCTCTGCGGCAAAGTTATACGTAGATCAGCTAAAGTTTTTCTATACAGGTATAGACA**CTGTATATATATACAG**TATAATAACTTTC**CTGTATATATATACAG**CTGCTCAAGAAAAGTTTAATGACTATTCGTCGCCCAAAAAGATGAATAAATCGGAGAAAGTATACTAGAGATT
Jungle Both	CGTGATAAGCTCTGCGGCAAAGTTATACGTAGATCAG**CTGTATATATATACAG**CAGGTATAGACA**CTGTATATATATACAG**TATAATAACTTTC**CTGTATATATATACAG**CTGCTCAAGAAAAGTTTAATGACTATTCGTCGCCCAAAAAGATGAATAAATCGGAGAAAGTATACTAGAGATT

Figure S5. Promoter design and conceptual illustration of chimeric promoters binding with LexA dimer. Additional SOS box (AT repeat) was placed in different part of the V.RecA promoter with the same 7bp gap. -35 and -10 remained unaltered.

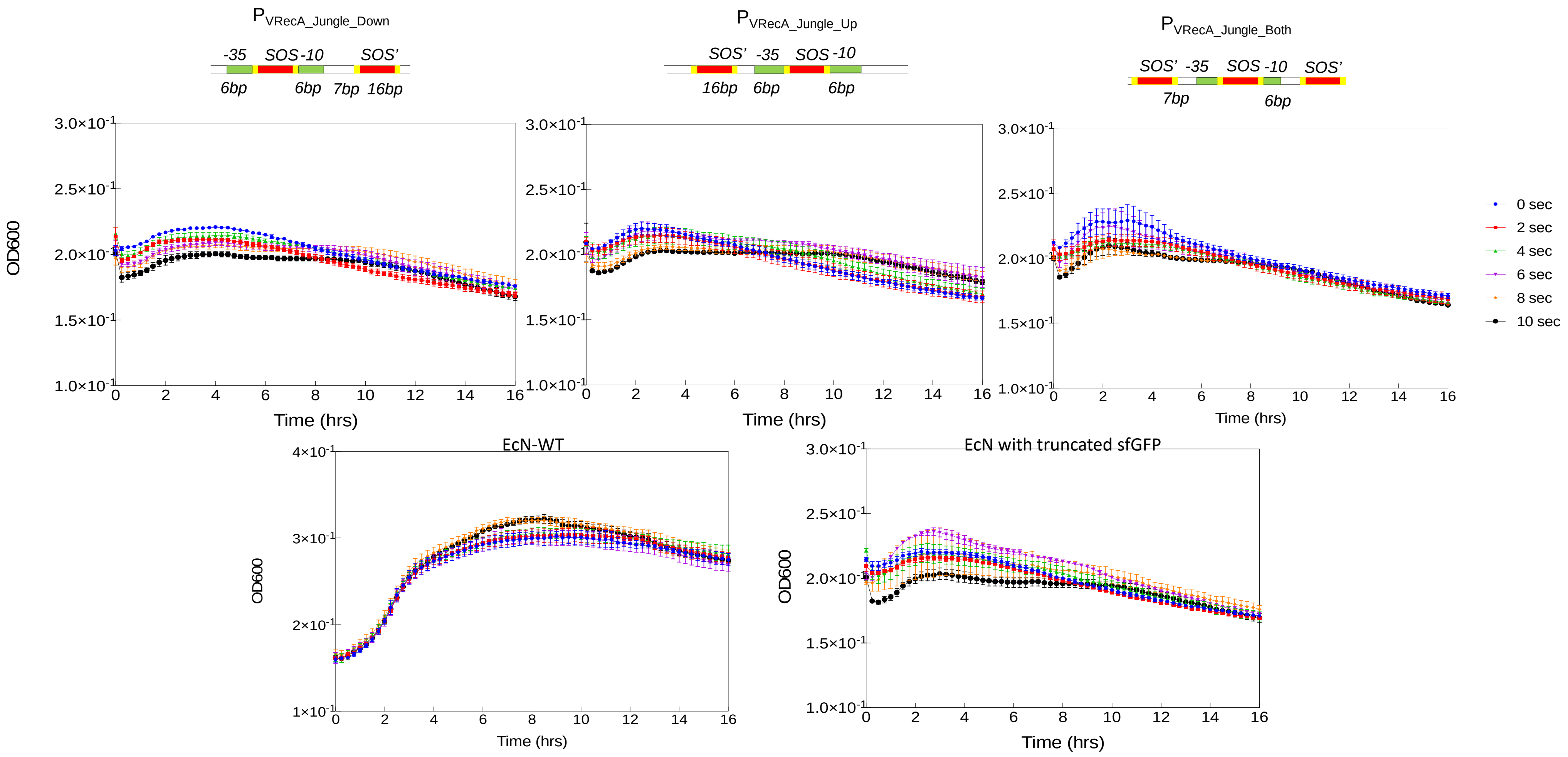

Figure S6. OD growth curve for all three V.RecA promoters with different No of SOS binding box tested with (0 ~ 10 sec) 302nm UV exposure, transformed *E. coli Nissle 1917* was supplemented in M9 medium with 0.4% glucose and Kanamycin 60 µg/ml. OD growht curve for EcN wild type and EcN transformed with truncated sfGFP backbone were shown at the bottom. N=3.

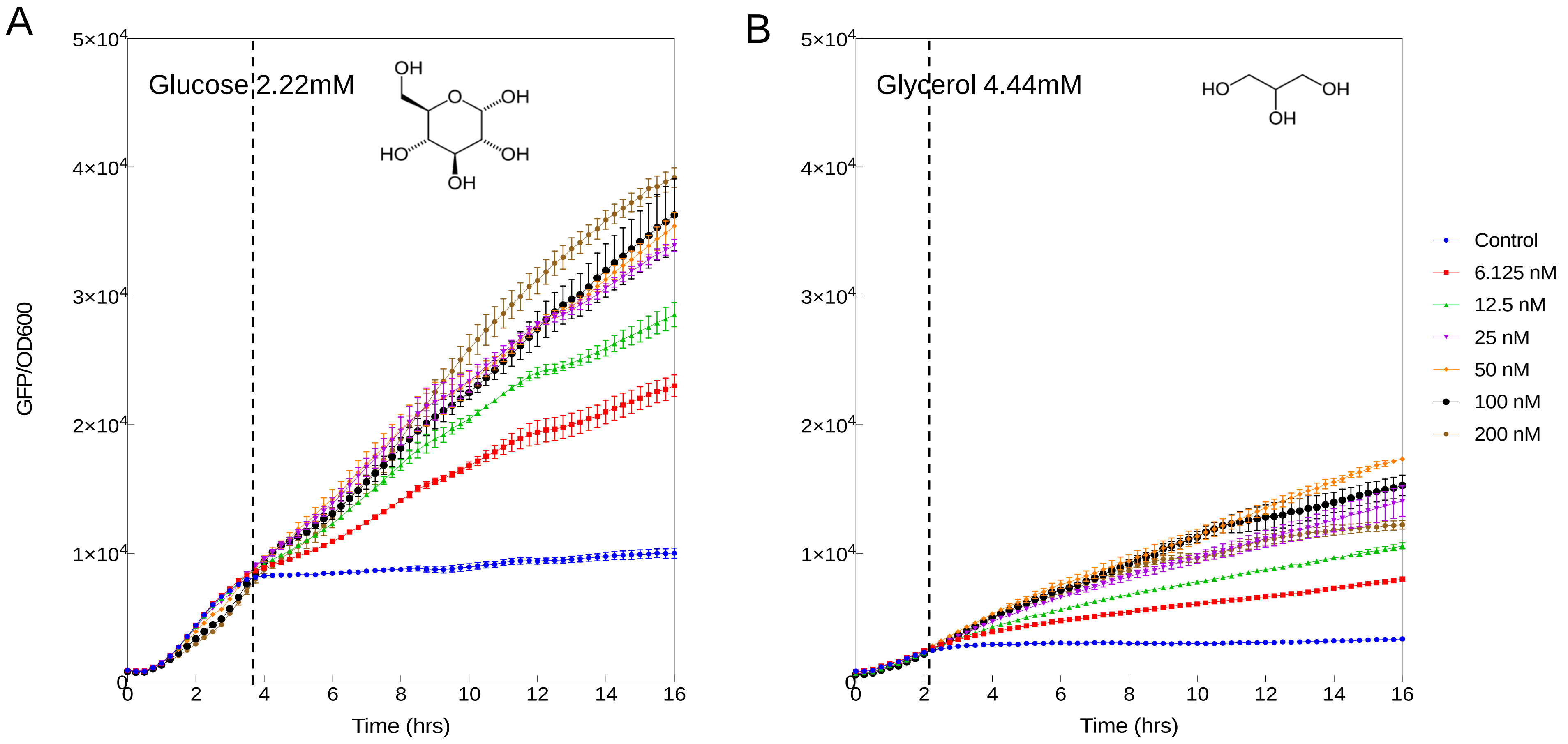

Figure S7. Amplifier circuits performance using different carbon source. The system is tested in M9 media supplemented with A) 2.22mM of Glucose and B) 4.44mM of Glycerol. Both experiments were done with a concentration gradient of Mitomycin C (0nM;6.125nM;12.5nM; 25nM;50nM;100nM; 200nM) over 16hrs. The bottom row shows the OD600 profile of both experiments. N=3

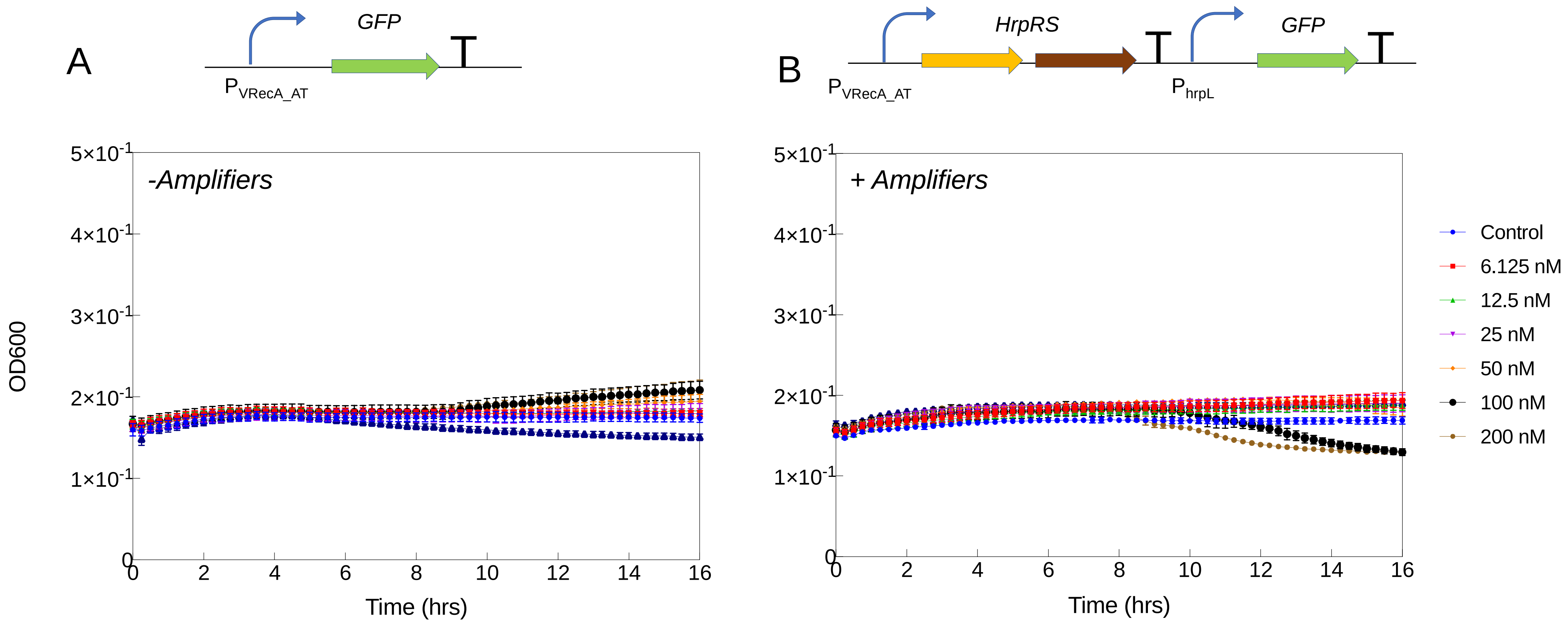

Figure S8. Bacteria growth with and without amplifiers addition
OD600 of EcN transformed with the V.RecA_gfp plasmid with and without the addition of HrpRS transcriptional amplifiers modules. A) V.RecA wt promoter fused with GFP. B) Optimized V.RecA promoter fused with HrpRS subunits followed by a GFP output controlled by PhrpL. Both systems were tested with a concentration gradient of Mitomycin C (0nM;6.125nM;12.5nM; 25nM;50nM;100nM; 200nM) over 16hrs. N=3

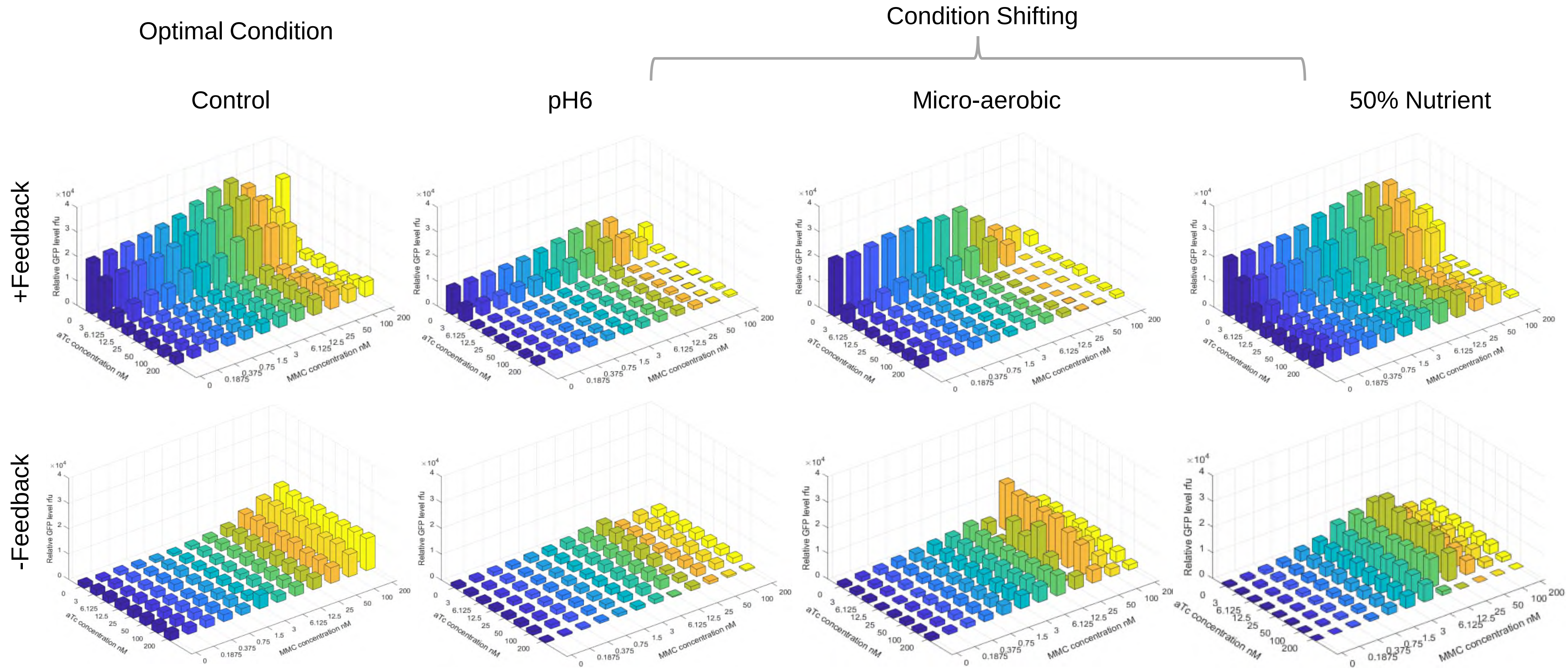

Figure S9. Increased robustness with negatively tuned amplifiers. 3D bar chart representing the performance of V.RecA operational amplifier system subjected to different type of environmental disturbances (Acidic pH; Oxygen limitation and Nutrient limitation). Top row: V.RecA amplifier system containing the negative feedback modules tested with MMC and aTc from 0 to 200 nM under different conditions. Bottom row: V.RecA amplifier system without the negative feedback modules tested with MMC and aTc from 0 to 200 nM under different conditions. Microaerobic condition was achieved using 100 uL mineral oil sealing the 96 well plates. pH was adjusted by addition of 1M HCl. Optimal condition was defined as pH 7.5 at 37 ºC in a M9 media supplemented with 4%(w/v) Glycerol without oil sealing.

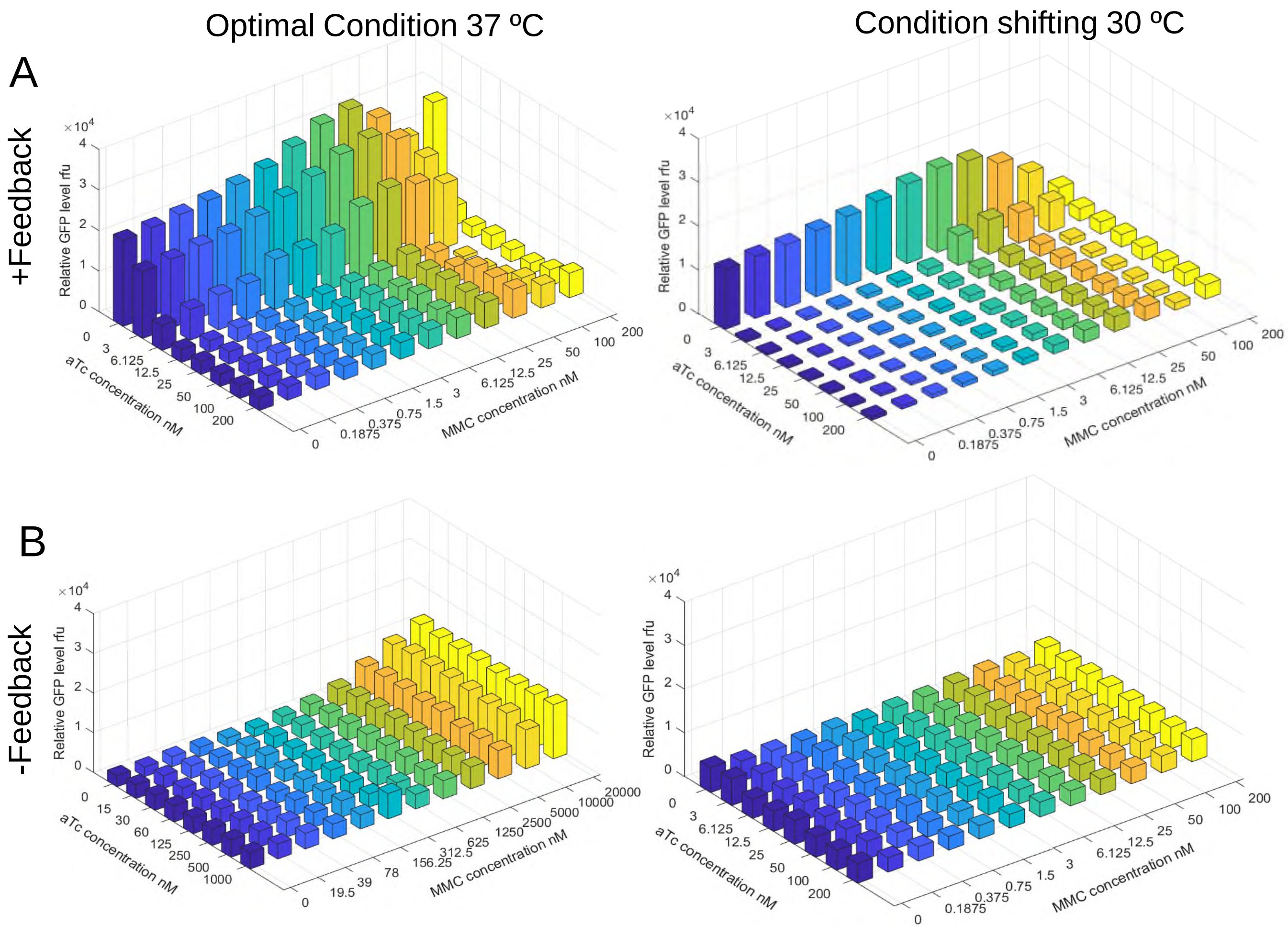

Figure S10. System robustness against temperature shift. 3D bar chart representing the performance of V.RecA operational amplifier system subjected to temperature shift. A) V.RecA amplifier system containing the negative feedback modules tested with MMC and aTc from 0 to 200 nM. B) V.RecA amplifier system without the negative feedback modules tested with MMC and aTc from 0 to 200 nM.

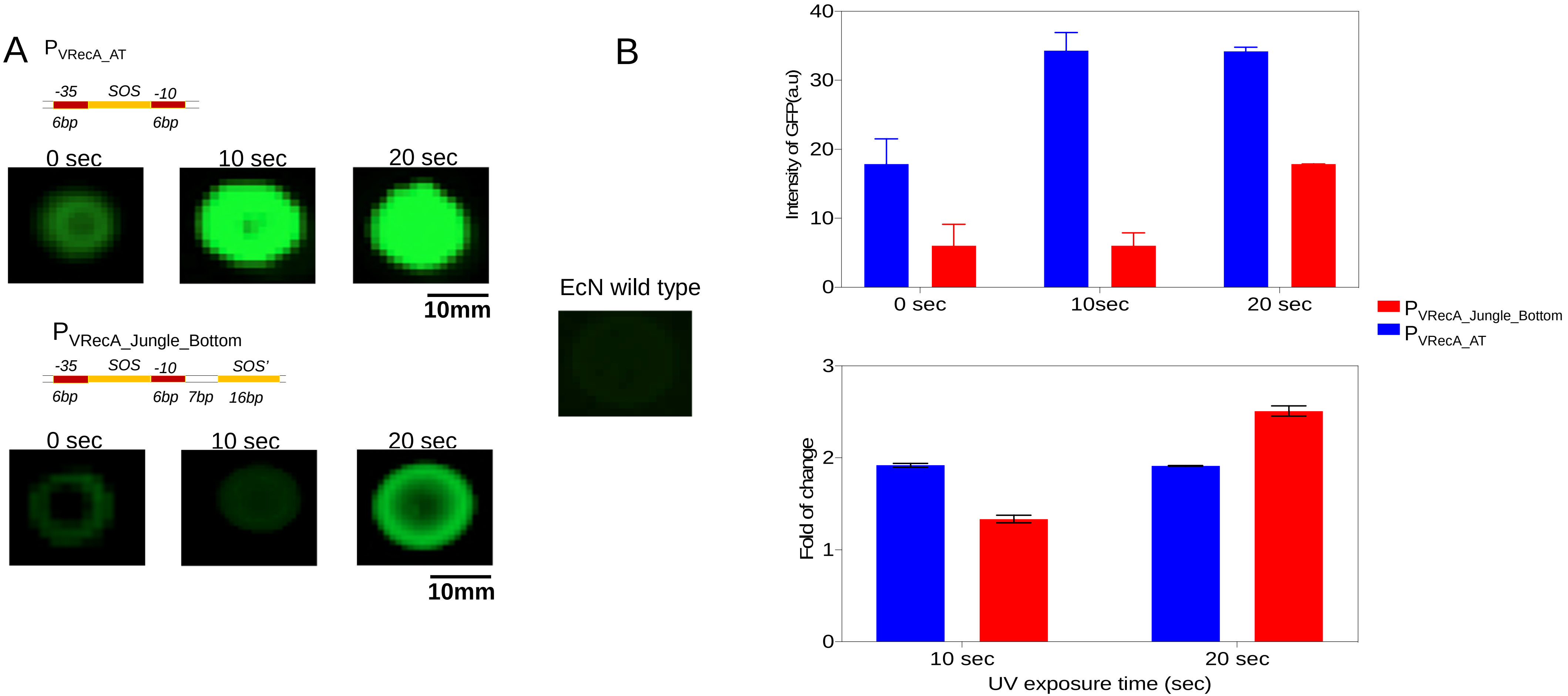

Figure S11. Potential application in optogenetics & biofilm engineering.
Optogenetic induction using macro-colonies. A) Top: Image of EcN colony transformed with optimized V.RecA-sfGFP circuits followed by exposure to 302nm UV for 10 sec, 20 sec respectively. Bottom: Image of EcN colony transformed with optimized V.RecA(Jungle_Bottom) following the same procedures. Image was taken under channel FITC. Intensity inverted for better visualization. B) Calculated mean intensity of green fluorescence were normalized against wild type control. Both images were processed using the same setting using ImageJ. N=3.

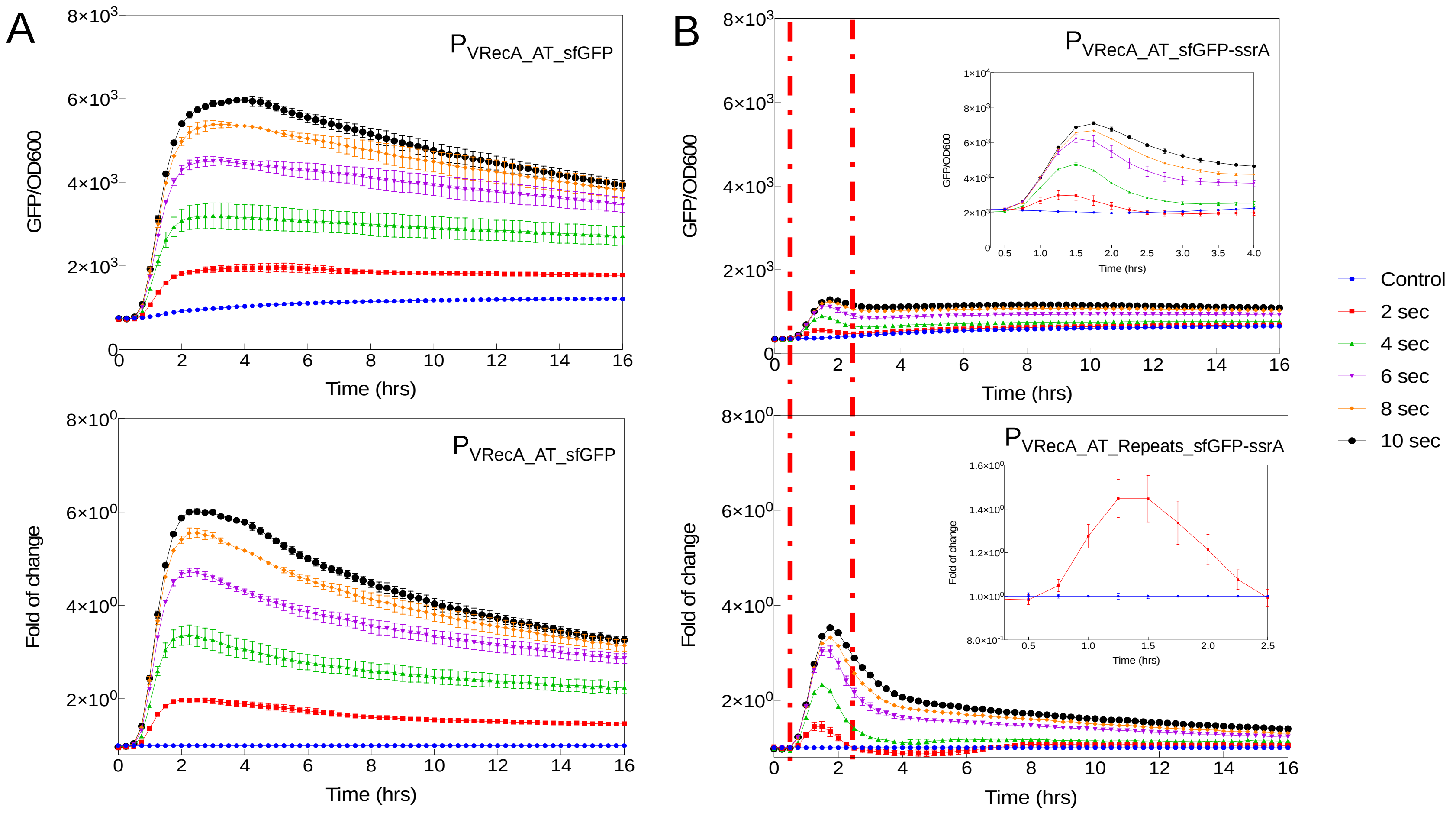

Figure S12. System dynamics with ssrA tagged sfGFP.

Circuits performance of V.RecA(ATrepeat) with ssrA tagged sfGFP. V.RecA with A) normal sfGFP and B) ssrA tagged sfGFP. Fold of change upon induction was normalized to highlight the expression window upon 2sec induction. Region highlighted with dash line is shown in the top right window. System kinetics were measured for 16 hrs after the exposure of 320 nm UV at a duration gradient (0 sec; 2 sec; 4 sec; 6 sec; 8 sec and 10 sec). N=3.

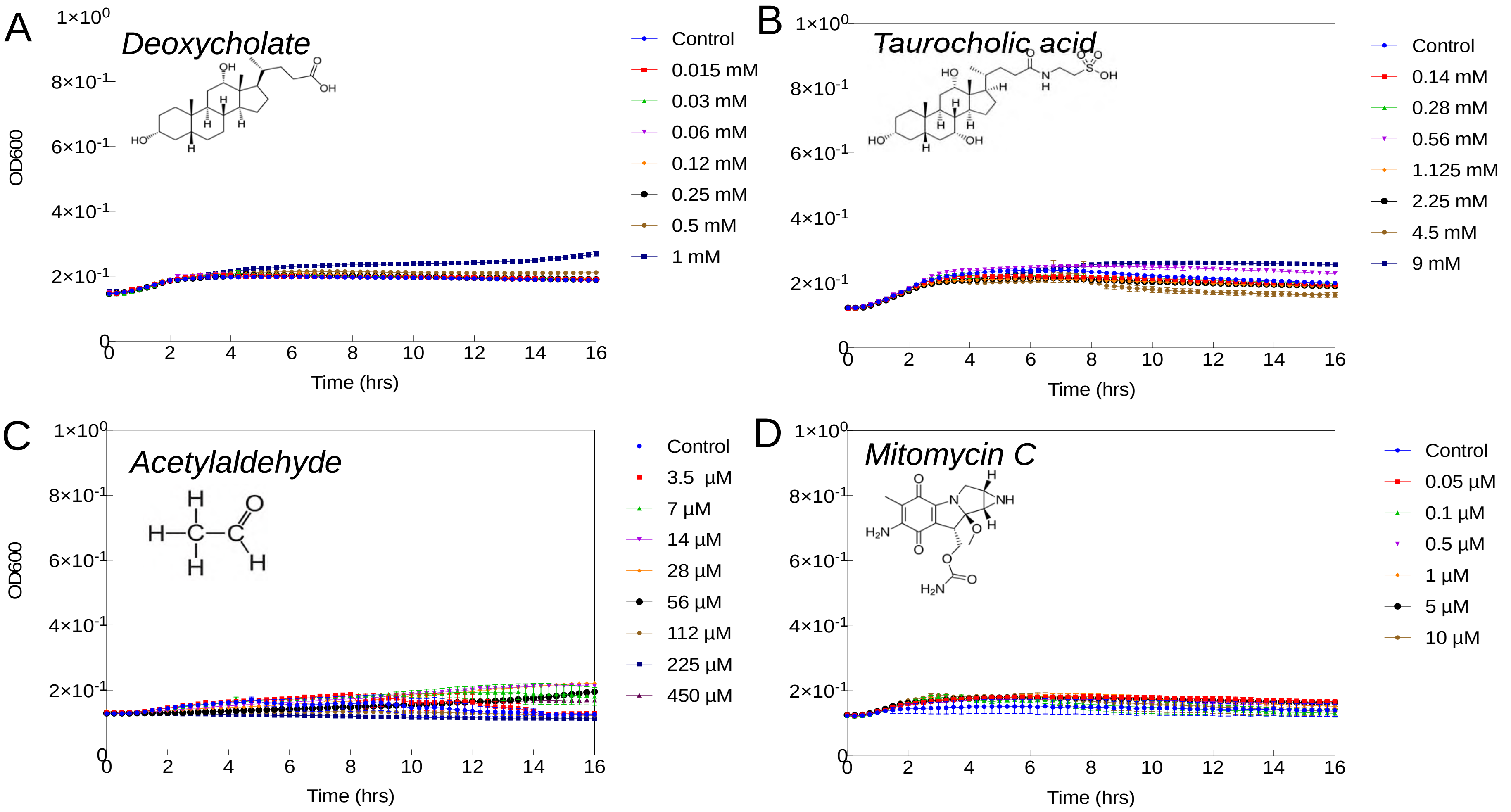

Figure S13. OD Growth of transformed bacteria with different secondary bile salts.
OD600 of EcN transformed with optimized V.RecA promoter in detecting colon cancer related carcinogens. A) Deoxycholate B) Taurocholic acid and C) Acetylaldehyde. D) Mitomycin C. N=3.

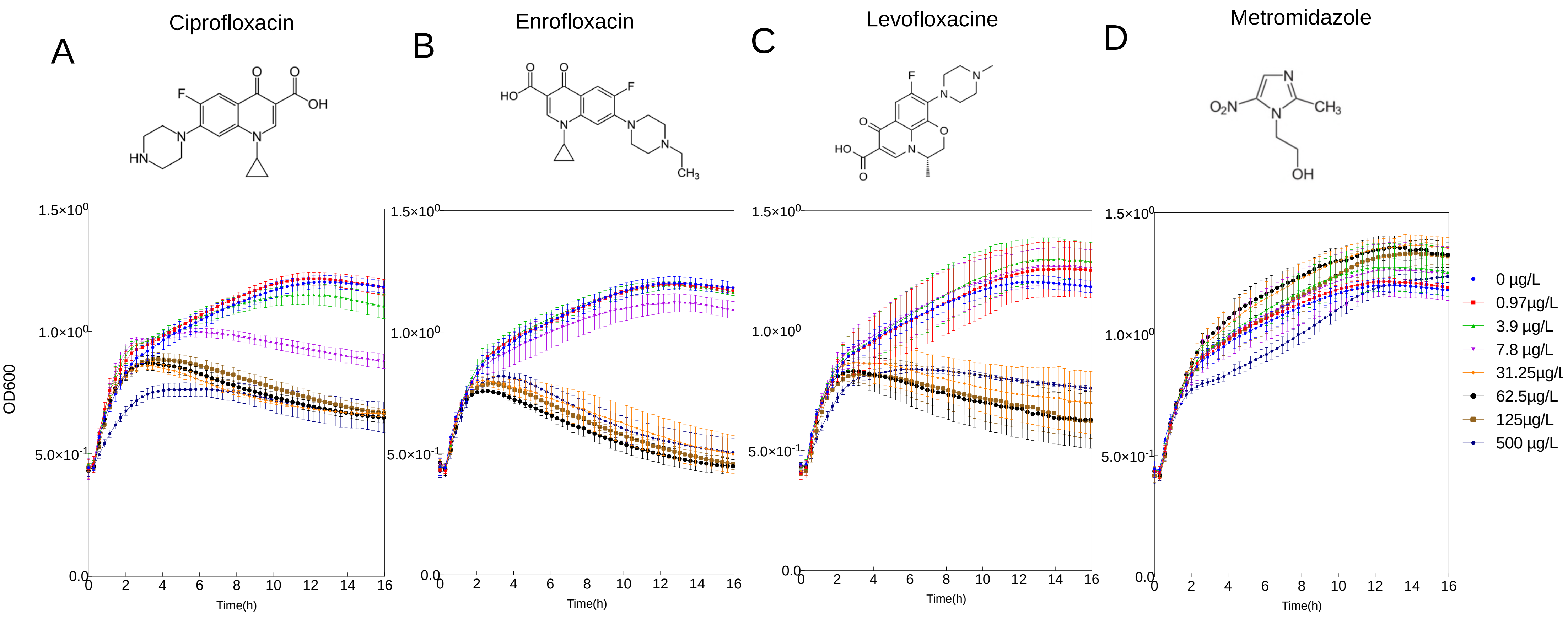

Figure S14. OD Growth of transformed bacteria with different antibiotics used in agriculture & animal feedstocks.
OD600 of EcN transformed with optimized V.RecA promoter in detecting commonly used carcinogenic antibiotics. A) Ciprofloxacin B) Enrofloxacin C) metromidazole and D) Levofloxacine. N=3.

$P_{VRecA\_AT-sfGFP}$

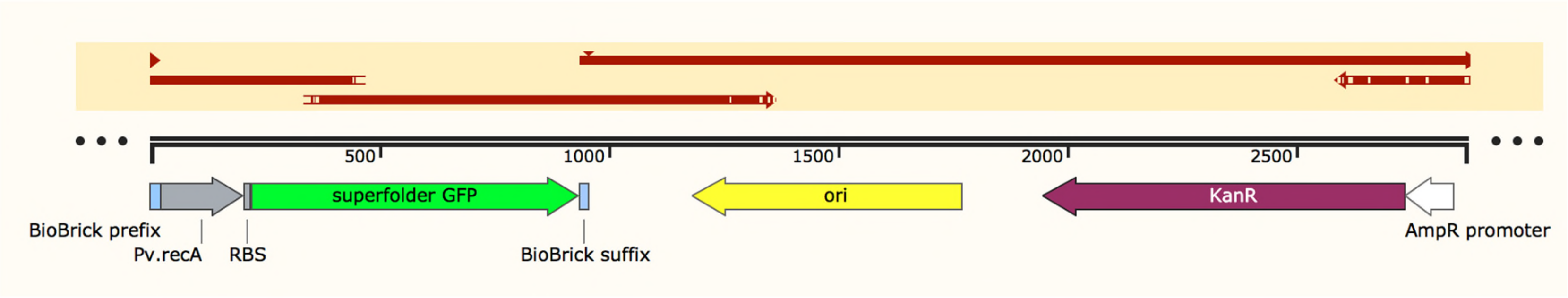

$P_{VRecA\_AT-sfGFP-ssrA}$

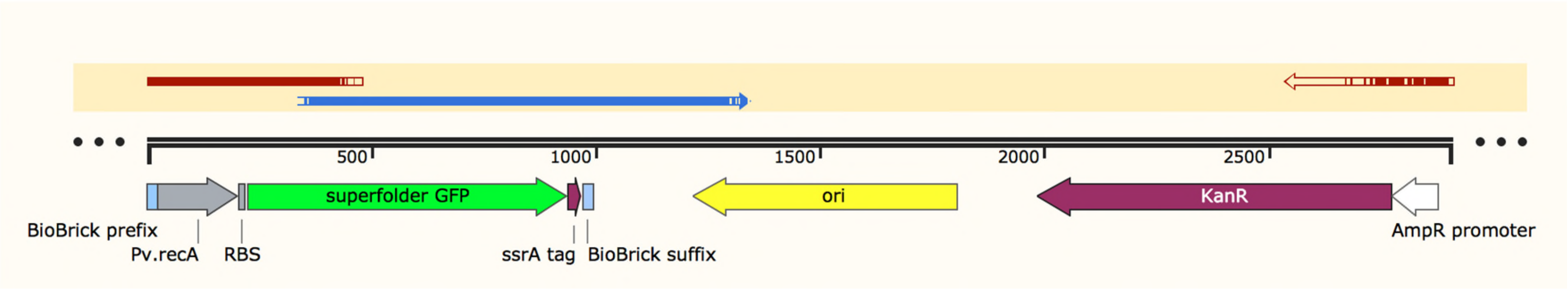

Figure S15. Quality check for plasmids integrity after 40hrs. Sequence alignment shown on top indicated sequence reads and plasmids map shown below. Mismatches are indicated as white space.
