# Supplementary material for: Redesign of ultrasensitive and robust RecA gene circuit to sense DNA damage
Source: Microb Biotechnol. 2021 Mar 4;14(6):2481–96. doi: 10.1111/1751-7915.13767 (PMC8601168; doi:10.1111/1751-7915.13767)
Supplement: Supplementary file 2 [file MBT2-14-2481-s001.pdf]

Supplementary information

Supplementary tables and plasmid maps

Redesign of ultra-sensitive and robust RecA gene circuit to sense DNA damage

Jack X. Chen[a], Boon Lim[a], Harrison Steel[a], Yizhi Song[a], Mengmeng Ji[b] and Wei E. Huang[a*]

[a]Department of Engineering Science, University of Oxford, Parks Road, Oxford, OX1 3PJ, United Kingdom
[b] Oxford Suzhou Centre for Advanced Research, Suzhou, 215123, China

*Corresponding author:
Wei E. Huang
Department of Engineering Science, University of Oxford, Parks Road, OX1 3PJ, Oxford, United Kingdom.
Tel.: +44 1865 283786
Email: wei.huang@eng.ox.ac.uk

Running title: Ultrasensitive DNA damage biosensor in EcN

Keywords: synthetic biology, DNA damage, biosensor *E. coli* Nissle 1917, *Vibrio natriegens*, *recA*, LexA, SOS system, UV.

Keywords: synthetic biology, DNA damage sensor, biosensor *E. coli* Nissle 1917, *Vibrio natriegens*, RecA SOS system, UV

Native *E. coli* Nissle 1917 RecA Promoter
CACTTGATACTGTATGAGCATACAGTATAATTGCTTCAACAGAACATATTGACTATCCGG TATTACCCGGCATGACAGGAGTAAAAATG

Native *V. natriegens* RecA promoter
CGTGATAAGCTCTGCGGCAAAGTTATACGTAGATCAGCTAAAGTTTTTCTATACAGGTA TAGACACTGTATGAATCAACAGTATAATAACTTTCATTGCTGAGCGATTAACTGCTCAAG AAAAGTTTAATGACTATTCGTCGCCCAAAAAGATG

Native *E. coli* K12 RecA promoter
CACTTGATACTGTATGAGCATACAGTATAATTGCTTCAACAGAACATATTGACTATCCGG TATTACCCGGCATGACAGGAGTAAAAATG

TTGATA is -35 sequence

TATAAT is -10 sequence

ATG is the start codon of the protein

Table S1. Alignment of SOS promoters and LexA binding motifs in different bacteria.

Species Name	LexA binding site	Ref
Citrobacter youngae	ATGACG**CCATTACTGT**ATAAAAAA**ACAGGTACAA**ATA	(Jacoby *et al.*, 2011)
Pseudomonas aeruginosa	TAATCC**CAGTCACTGG**ATAAAAAC**ACAGAGCGAC**GAC	(Cirz *et al.*, 2006)
Shewanella piezotolerans	CTGATT**AAATTACTGT**ATATACTA**ACAGGTACTG**TAT	(Jian *et al.*, 2015)
Escherichia coli K-12.MG1655	AAACAC**TTGATACTGT**ATGAGCAT**ACAGTATAAT**TGC	This study
Escherichia coli Nissle 1917	AAACAC**TTGATACTGT**ATGAGCAT**ACAGTATAAT**TGC	This study
Vibrio natrigene	CAGGTA**TAGACACTGT**ATGAATCA**ACAGTATAAT**AAC	This study
Salmonella entrica	AATCAC**CTTTAACTGT**ATATACTC**ACAGCATGAT**TGT	(Ganai *et al.*, 2009)

Table S2. Comparison of SOS promoters and LexA binding motifs in different *Vibrio* spp. with different doubling time.

Species Name	LexA binding site	Doubling time (min)	Ref
Vibrio cholerae	CAGGGG**TGGACACTGT**ACAAATCA**ACAGTATAAT**GAA	~40	(Gibson *et al.*, 2018)
Vibrio natriegens	CAGGTA**TAGACACTGT**ATGAATCA**ACAGTATAAT**AAC	~10	(Weinstock *et al.*, 2016)
Vibrio fischeri	AGCATA**GCACAGCTGT**ATATACTC**ACAGTGAACT**GTA	~280	(Lee and Ruby, 1994)
Vibrio parahaemolyticus	CAGGTA**TAGACACTGT**ATGAATCA**ACAGTATAAT**GAC	~10	(Phan *et al.*, 2015)

Vibrio vulnificus	CAGGGG**TAGACACTGT**ACAAATCA**ACAGTATAAT**GAG	~20	(Dryselius *et al.*, 2008)

Sigma factor -35 & -10 shown in underlined and bolded font
LexA binding ~~(~~CTGTN$_8$ACAG ~~)~~motif shown in cyan font

Table S3. Medical usage of mitomycin C in treating the different type of tumour.

Targeting diseases	Method	Dosage (µM)	Ref
Esophageal carcinoma	IV	12.1	(Brierley *et al.*, 2001)
Anal cancer	IV	20	(Ghosn *et al.*, 2015)
Breast cancer	IV	22.1	(Verweij and Pinedo, 1990)
Bladder tumours	IV	23.92	(Jeong *et al.*, 2005)

Table S4. Sequence for other modules used in this study.

Parts name	Sequence 5'→ 3'	Ref
pV.RecA_JungleUP	CGTGATAAGCTCTGCGGCAAAGTTATACGTAGATCACTGTATATATAT<br>ACAGACAGGTATAGACACTGTATATATATATACAGTATAATAACTTTCATT<br>GCTGAGCGATTAACTGCTCAAGAAAAGTTTAATGACTATTCGTCGCCC<br>AAAAAGATGAATAAATCGGAGAAAGTATACTAGAGATT	This work
pV.RecA_JungleDown	CGTGATAAGCTCTGCGGCAAAGTTATACGTAGATCAGCTAAAGTTTTT<br>CTATACAGGTATAGACACTGTATATATATATACAGTATAATAACTTTCCTG<br>TATATATATACAGCTGCTCAAGAAAAGTTTAATGACTATTCGTCGCCCA<br>AAAAGATGAATAAATCGGAGAAAGTATACTAGAGATT	
pV.RecA_JungleBoth	CGTGATAAGCTCTGCGGCAAAGTTATACGTAGATCAGCTGTATATATA<br>TACAGCAGGTATAGACACTGTATATATATATACAGTATAATAACTTTCCTG<br>TATATATATACAGCTGCTCAAGAAAAGTTTAATGACTATTCGTCGCCCA<br>AAAAGATGAATAAATCGGAGAAAGTATACTAGAGATT	
pV.RecA_ATrepeat	CGTGATAAGCTCTGCGGCAAAGTTATACGTAGATCAGCTAAAGTTTTT<br>CTATACAGGTATAGACACTGTATATATATATACAGTATAATAACTTTCATT<br>GCTGAGCGATTAACTGCTCAAGAAAAGTTTAATGACTATTCGTCGCCC<br>AAAAAGATGAATAAATCGGAGAAAGTATACTAGAGATT	
pV.RecA_ATandem	CGTGATAAGCTCTGCGGCAAAGTTATACGTAGATCAGCTAAAGTTTTT<br>CTATACAGGTATAGACACTGTAAAAAAAAACAGTATAATAACTTTCATT<br>GCTGAGCGATTAACTGCTCAAGAAAAGTTTAATGACTATTCGTCGCCC<br>AAAAAGATGAATAAATCGGAGAAAGTATACTAGAGATT	
pV.RecA_WT	CGTGATAAGCTCTGCGGCAAAGTTATACGTAGATCAGCTAAAGTTTTT<br>CTATACAGGTATAGACACTGTATGAATCAACAGTATAATAACTTTCATT<br>GCTGAGCGATTAACTGCTCAAGAAAAGTTTAATGACTATTCGTCGCCC<br>AAAAAGATGAATAAATCGGAGAAAGTATACTAGAGATT	
pE.RecA	CACTTGATACTGTATGAGCATACAGTATAATTGCTTCAACAGAACATAT<br>TGACTATCCGGTATTACCCGGCAT	
sfGFP	ATGAGCAAAGGAGAAGAACTTTTCACTGGAGTTGTCCCAATTCTTGTT<br>GAATTAGATGGTGATGTTAATGGGCACAAATTTTCTGTCCGTGGAGAG<br>GGTGAAGGTGATGCTACAAACGGAAAACTCACCCTTAAATTTATTTGC<br>ACTACTGGAAAACTACCTGTTCCGTGGCCAACACTTGTCACTACTCTG<br>ACCTATGGTGTTCAATGCTTTTCCCGTTATCCGGATCACATGAAACGG<br>CATGACTTTTTCAAGAGTGCCATGCCCGAAGGTTATGTACAGGAACG<br>CACTATATCTTTCAAAGATGACGGGACCTACAAGACGCGTGCTGAAG<br>TCAAGTTTGAAGGTGATACCCTTGTTAATCGTATCGAGTTAAAGGGTA<br>TTGATTTTAAAGAAGATGGAAACATTCTTGGACACAAACTCGAGTACA<br>ACTTTAACTCACACAATGTATACATCACGGCAGACAAACAAAAGAATG<br>GAATCAAAGCTAACTTCAAAATTCGCCACAACGTTGAAGATGGTTCCG<br>TTCAACTAGCAGACCATTATCAACAAAATACTCCAATTGGCGATGGCC<br>CTGTCCTTTTACCAGACAACCATTACCTGTCGACACAATCTGTCCTTT<br>CGAAAGATCCCAACGAAAAGCGTGACCACATGGTCCTTCTTGAGTTT<br>GTAACTGCTGCTGGGATTACACATGGCATGGATGAGCTCTACAAA	(Shaner *et al.*, 2005)
GFP	ATGCGTAAAGGAGAAGAACTTTTCACTGGAGTTGTCCCAATTCTTGTT<br>GAATTAGATGGTGATGTTAATGGGCACAAATTTTCTGTCAGTGGAGAG<br>GGTGAAGGTGATGCAACATACGGAAAACTTACCCTTAAATTTATTTGC<br>ACTACTGGAAAACTACCTGTTCCATGGCCAACACTTGTCACTACTTTC<br>GGTTATGGTGTTCAATGCTTTGCGAGATACCCAGATCATATGAAACAG<br>CATGACTTTTTCAAGAGTGCCATGCCCGAAGGTTATGTACAGGAAAGA<br>ACTATATTTTTCAAAGATGACGGGAACTACAAGACACGTGCTGAAGTC<br>AAGTTTGAAGGTGATACCCTTGTTAATAGAATCGAGTTAAAAGGTATT<br>GATTTTAAAGAAGATGGAAACATTCTTGGACACAAATTGGAATACAAC<br>TATAACTCACACAATGTATACATCATGGCAGACAAACAAAAGAATGGA<br>ATCAAAGTTAACTTCAAAATTAGACACAACATTGAAGATGGAAGCGTT<br>CAACTAGCAGACCATTATCAACAAAATACTCCAATTGGCGATGGCCCT<br>GTCCTTTTACCAGTACTAGTAGCGGCCGCTGCAGTCCGGCAAAAAAA<br>CGGGCAAGGTGTCACCACCCTGCCCTTTTTCTTTAAAACCGAAAAGAT<br>TACTTCGCGTTATGCAGGCTTCCTCGCTCACTGA	(Shaner *et al.*, 2005)
ssrA_LVA	GCAGCGAACGACGAAAATTACGCCCTTGCAGCG	(Purcell *et al.*, 2012)
RBS (BBa_B0030)	AAAGAGGAGAAA	(Part:BBa B0030 - parts.igem.org)

HrpR	ATGAGTACAGGCATCGATAAGGACGTCCGAGAGTGTTGGGGCGTAACTGCATTATCAGCGGGTCATCAAATTGCAATGAATAGCGCGTTTCTGGATATGGACTTGCTGTTGTGCGGGGAAACCGGCACCGGCAAGGACACACTGGCCAACCGCATTCACGAGTTGTCCAGCAGGTCGGGACCCTTTGTGGGCATGAACTGCGCCGCCATTCCCGAGTCGCTGGCAGAGAGCCAGTTATTCGGTGTGGTCAACGGTGCATTCACCGGCGTATGCCGGGCTCGCGAGGGCTACATAGAGGCCTCCAGTGGTGGCACCTTGTACCTGGATGAAATCGACAGCATGCCGTTGAGCCTGCAAGCCAAACTGCTGCGTGTGTTGGAGAGTCGAGGTATCGAGCGTCTGGGCTCGACCGAATTTATCCCGGTGGATCTGCGGATCATTGCCTCGGCCCAGCGGCCACTGGATGAACTGGTGGAACAAGGACTTTTCCGTCGCGACCTGTTTTTTCGGCTCAACGTGCTGACGCTTCACTTGCCAGCCTTGCGCAAACGTCGTGAACAGATCCTGCCATTGTTCGACCAGTTCACCCAGGGTATCGCTGCCGAGTTCGGACGTCCCGCTCCTGCGCTGGACAGCGGGCGTGTGCAGCTGCTGCTCAGCCACGACTGGCCGGGCAACATCCGCGAATTGAAGTCTGCGGCCAAGCGCTTCGTACTCGGCTTCCCCTTGCTGGGCGCCGACCCTGTGGAAGCGCTTGACCCTGCCACGGGGCTGCGCACGCAAATGCGCATCATCGAGAAAATGCTCATCCAGGATGCCTTGAAGCGGCACAGGCACAATTTCGACGCGGTGCTTCAGGAGTTGGAGTTGCCAAGACGCACCCTGTATCACCGCATGAAGGAACTGGGAGTTGCAGCGCCGATCGCTGCGACGGCCGGGGTCTAATAA	(Wang *et al.*, 2014)
HrpS	ATGAGTCTTGATGAAAGGTTTGAGGATGATCTGGACGAGGAGCGGGTTCCGAATCTGGGGATAGTTGCCGAAAGTATTTCGCAACTGGGTATCGACGTGCTGCTATCGGGTGAGACCGGCACGGGCAAAGACACGATTGCCCGACGGATTCATGAGATGTCAGGCCGCAAAGGGCGCCTGGTGGCGATGAATTGCGCGGCCATTCCGGAGTCCCTCGCCGAGAGCGAGTTATTCGGCGTGGTCAGCGGTGCCTACACCGGCGCTGATCGCTCCAGAGTCGGTTATGTCGAAGCGGCGCAGGGCGGCACGCTGTACCTGGATGAGATCGATAGCATGCCGCTGAGCCTGCAAGCCAAATTGCTGAGGGTGCTGGAAACCCGAGCGCTTGAACGGCTGGGTTCGACGTCGACGATCAAGCTGGATATCTGCGTGATCGCCTCCGCCCAATGCTCGCTGGACGACGCCGTCGAGCGGGGGCAGTTTCGTCGCGATCTGTATTTTCGCCTGAACGTCCTGACACTCAAGCTTCCTCCGCTACGTAACCAGTCTGATCGCATAGTTCCCCTGTTCACACGTTTTACGGCCGCCGCCGCGAGGGAGCTCGGTGTTCCCGTTCCCGATGTTTGCCCACTGCTGCACAAAGTGCTGCTGGGCCACGACTGGCCCGGCAATATCCGTGAGCTCAAGGCGGCAGCCAAACGCCATGTGCTGGGTTTCCCCTTGCTGGGCGCCGAGCCGCAGGGCGAAGAGCACTTGGCCTGTGGGCTCAAATCGCAATTGCGAGTGATCGAAAAAGCCCTGATTCAGGAGTCGCTCAAGCGCCACGACAATTGTGTGGATTCGGTAAGCCTGGAACTGGACGTGCCACGCCGTACGCTCTATCGACGCATCAAAGAATTGCAGATCTAATAA	(Wang *et al.*, 2014)
Terminator T-B15	CCAGGCATCAAATAAAACGAAAGGCTCAGTCGAAAGACTGGGCCTTTCGTTTTATCTGTTGTTTGTCGGTGAACGCTCTCTACTAGAGTCACACTGGCTCACCTTCGGGTGGGCCTTTCTGCGTTTATA	(Wang *et al.*, 2014)
Tdtomato	ATGGTGAGCAAGGGCGAGGAGGTCATCAAAGAGTTCATGCGCTTCAAGGTGCGCATGGAGGGCTCCATGAACGGCCACGAGTTCGAGATCGAGGGCGAGGGCGAGGGCCGCCCCTACGAGGGCACCCAGACCGCCAAGCTGAAGGTGACCAAGGGCGGCCCCCTGCCCTTCGCCTGGGACATCCTGTCCCCCCAGTTCATGTACGGCTCCAAGGCGTACGTGAAGCACCCCGCCGACATCCCCGATTACAAGAAGCTGTCCTTCCCCGAGGGCTTCAAGTGGGAGCGCGTGATGAACTTCGAGGACGGCGGTCTGGTGACCGTGACCCAGGACTCCTCCCTGCAGGACGGCACGCTGATCTACAAGGTGAAGATGCGCGGCACCAACTTCCCCCCCGACGGCCCCGTAATGCAGAAGAAGACCATGGGCTGGGAGGCCTCCACCGAGCGCCTGTACCCCCGCGACGGCGTGCTGAAGGGCGAGATCCACCAGGCCCTGAAGCTGAAGGACGGCGGCCACTACCTGGTGGAGTTCAAGACCATCTACATGGCCAAGAAGCCCGTGCAACTGCCCGGCTACTACTACGTGGACACCAAGCTGGACATCACCTCCCACAACGAGGACTACACCATCGTGGAACAGTACGAGCGCTCCGAGGGCCGCCACCACCTGTTCCTGGGGCATGGCACCGGCAGCACCGGCAGCGGCAGCTCCGGCACCGCCTCCTCCGAGGACAACAACATGGCCGTCATCAAAGAGTTCATGCGCTTCAAGGTGCGCATGGAGGGCTCCATGAACGGCCACGAGTTCGAGATCGAGGGCGAGGGCGAGGGCCGCCCCTACGAGGGCACCCAGACCGCCAAGCTGAAGGTGACCAAGGGCGGCCCCCTGCCCTTCGCCTGGGACATCCTGTCCCCCCAGTTCATGTACGGCTCCAAGGCGTACGTGAAGCACCCCGCCGACATCCCCGATTACAAGAAGCTGTCCTTCCCCGAGGGCTTCAAGTGGGAGCGCGTGATGAACTTCGAGGACGGCGGTCTGGTGACCGTGACCCAGGACTCCTCCCTGCAGGACGGCACGCTGATCTACAAGGTGAAGATGCGCGGCACCAACTTCCCCCCCGACGGCCCCGTAATGCAGAAGAAGACCATGGGCTGGGAGGCCTCCACCGAGCGCCTGTACCCCCGCGACGGCGTGCTGAAGGGCGAGATCCACCAGGCCCTGAAGCTGAAGGACGGCGGCCACTACCTGGTGGAGTTCAAGACCATCTACATGGCCAAGAAGCCCGTGCAACTGCCCGGCTACTACTACGTGGACACCAAGCTGGACATCACCTCCCACAA	(Shaner *et al.*, 2004)

	CGAGGACTACACCATCGTGGAACAGTACGAGCGCTCCGAGGGCCGC CACCACCTGTTCCTGTACGGCATGGACGAGCTGTACAAGTAA	
HrpV	ATGATTGAGGTAACGGAAAAGTCGGCATTTTACGCTCAGGTTGCCGC CCAGAGCCCAGCAGTATGGCCAGTGGCCAATGGCGTCGCGTTTGTC AGTCGGCGCGAACACCATGATTGGGGGATCGCCTTGCACATAGAAG GCCGCGCGTTGCGTCCCGATCAACTGCGTGATGCACTGCAACGACG TTTTATGGAGTCAGAGCGTTTCAATCACTACTTCCTGTTTCTGGACGT ACGACGCGACTTTGTGGTGTGGCACGCGGTCAACGAAAAACCGGGT TCCTACGCCAGCCTGGACGACATCCGCCGGCATGAGTTGATGCTGG CAGGGCTGGACCATTTGAGCGAGGAAATGCACTAA	(Wang *et al.*, 2014)
pTetO	TCCCTATCAGTGATAGAGATTGACATCCCTATCAGTGATAGAGATACT GAGCACATCAGCAGGACGCATGACCGAATTCATTAAATTTAACTTTAA GAAGGAGATATAAT	(Rogers *et al.*, 2015)
pHrpL	GCCGGATTATGTCCGCTGAGTGGGTCACGGTCCCGGATCAGTTCCCT TGCGAAGCTGACCGATGTTTTTGTGCCAAAAGCTGTTGTGGCAAAAAA CGGTTTGCGCAAAGTTTTGTATTACAAAGAATTTCACATTTTAAAATAT CTTTATAAATCAATCAGTTATTTCTATTTTTAAGCTGGCATGGTTATCG CTATAGGGCTTGTAC	(Wang *et al.*, 2014)

Fig S16. Circular plasmids map for all circuits listed in Table 2.

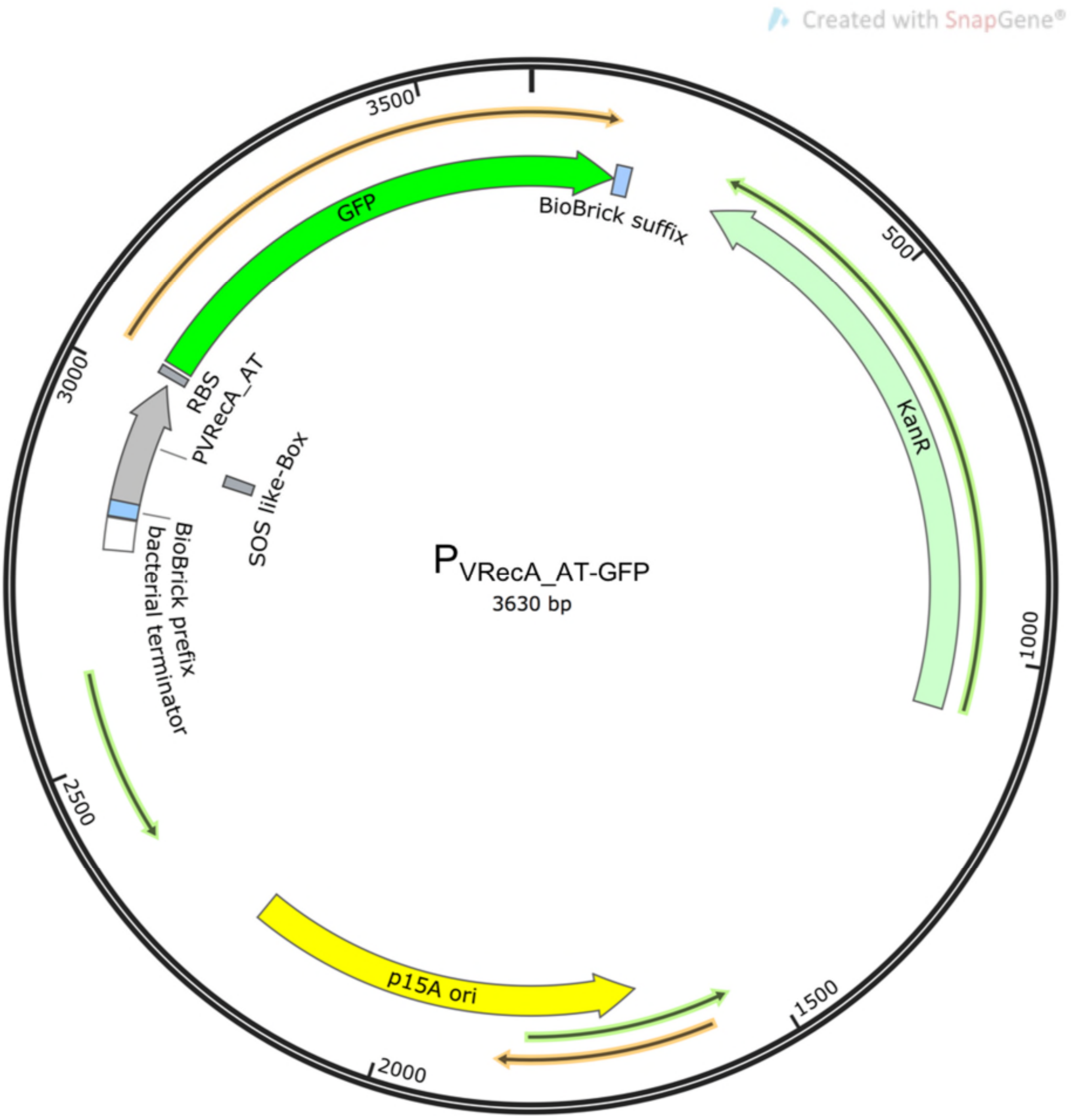

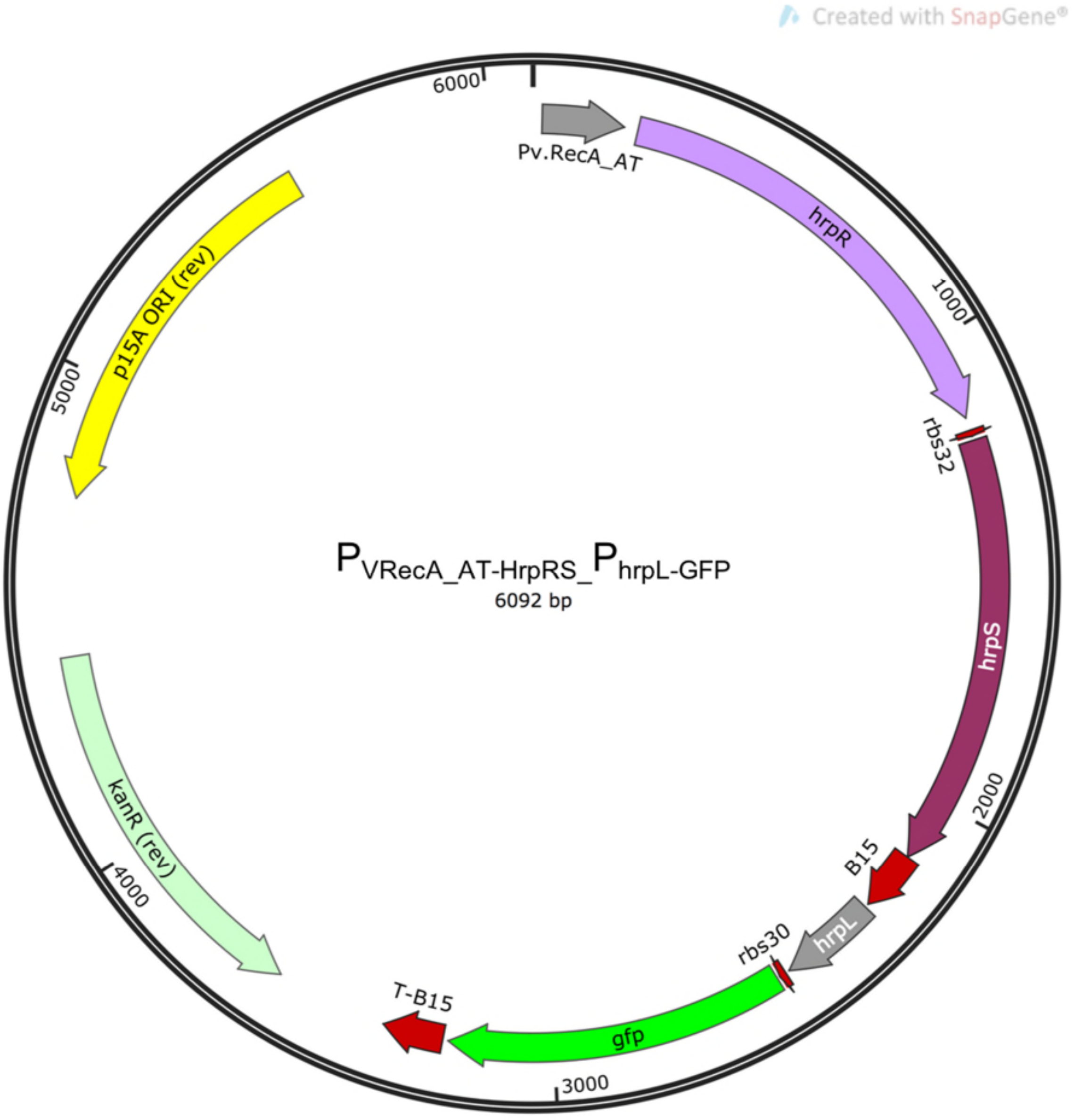
Created with SnapGene®
$P_{VRecA\_AT-HrpRS\_}P_{hrpL-GFP}$
6092 bp
Pv.RecA_AT
hrpR
rbs32
hrpS
B15
hrpL
rbs30
gfp
T-B15
kanR (rev)
p15A ORI (rev)
6000
1000
2000
3000
4000
5000

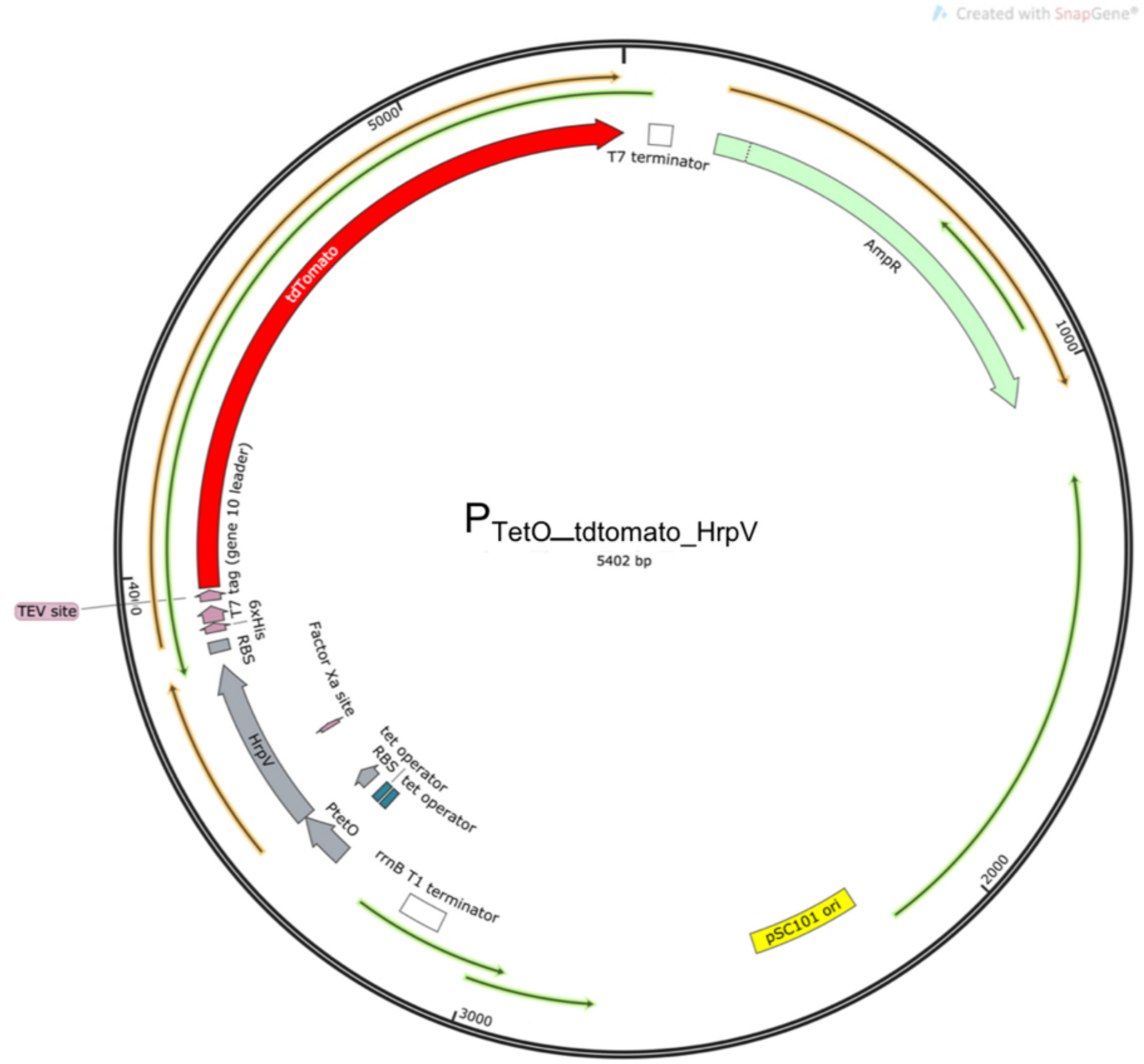

Created with SnapGene®
PTetO_tdtomato_HrpV
5402 bp
T7 terminator
AmpR
1000
2000
3000
4000
5000
pSC101 ori
rrnB T1 terminator
PtetO
HrpV
tet operator
RBS
tet operator
Factor Xa site
RBS
6xHis
T7 tag (gene 10 leader)
TEV site
tdTomato

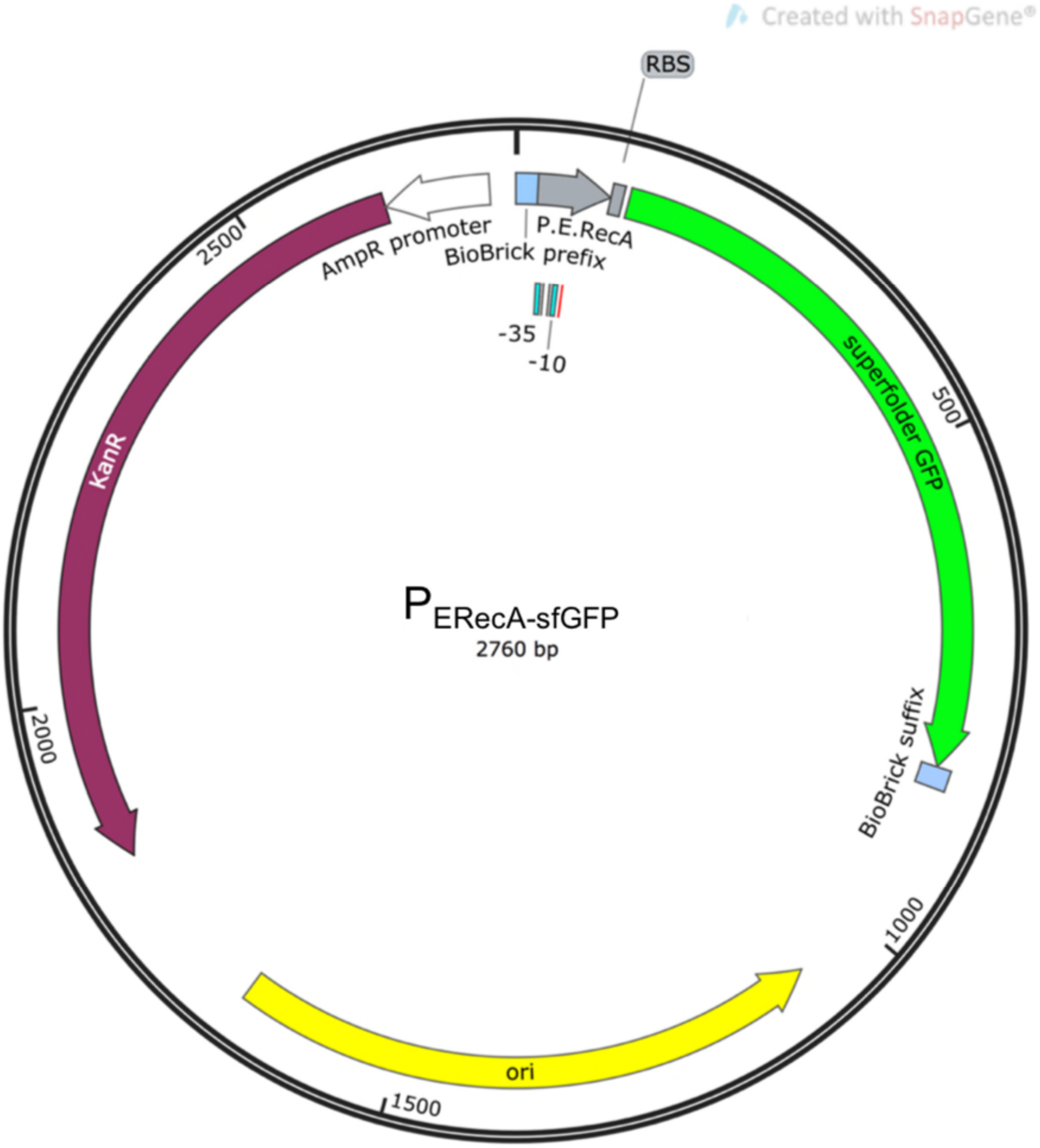
Created with SnapGene®
RBS
P.E.RecA
BioBrick prefix
AmpR promoter
-35
-10
superfolder GFP
BioBrick suffix
KanR
ori
500
1000
1500
2000
2500
P ERecA-sfGFP
2760 bp

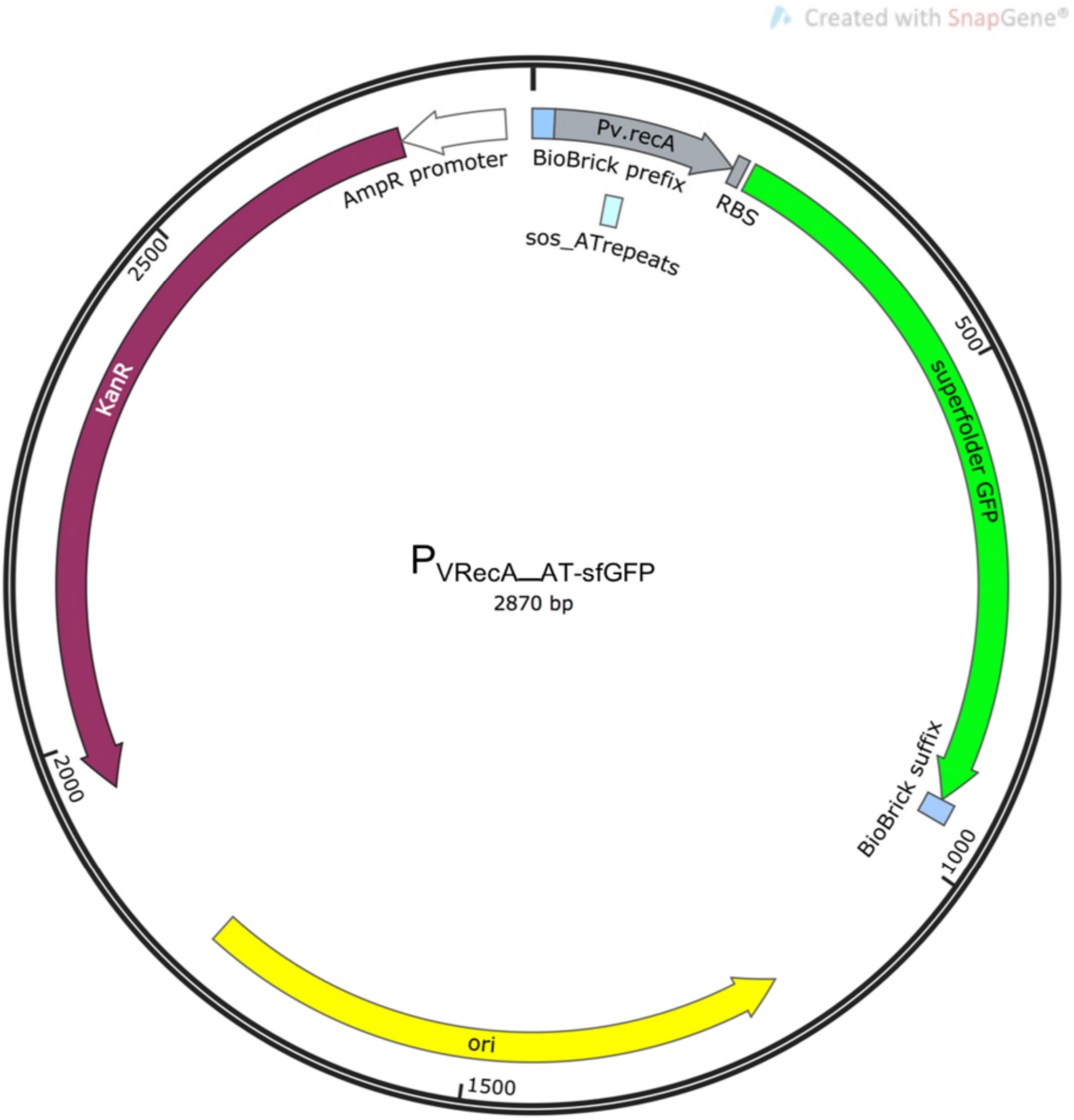
Created with SnapGene®
Pv.recA
BioBrick prefix
RBS
sos_ATrepeats
AmpR promoter
2500
KanR
500
superfolder GFP
$P_{VRecA\_AT\text{-}sfGFP}$
2870 bp
2000
BioBrick suffix
1000
ori
1500

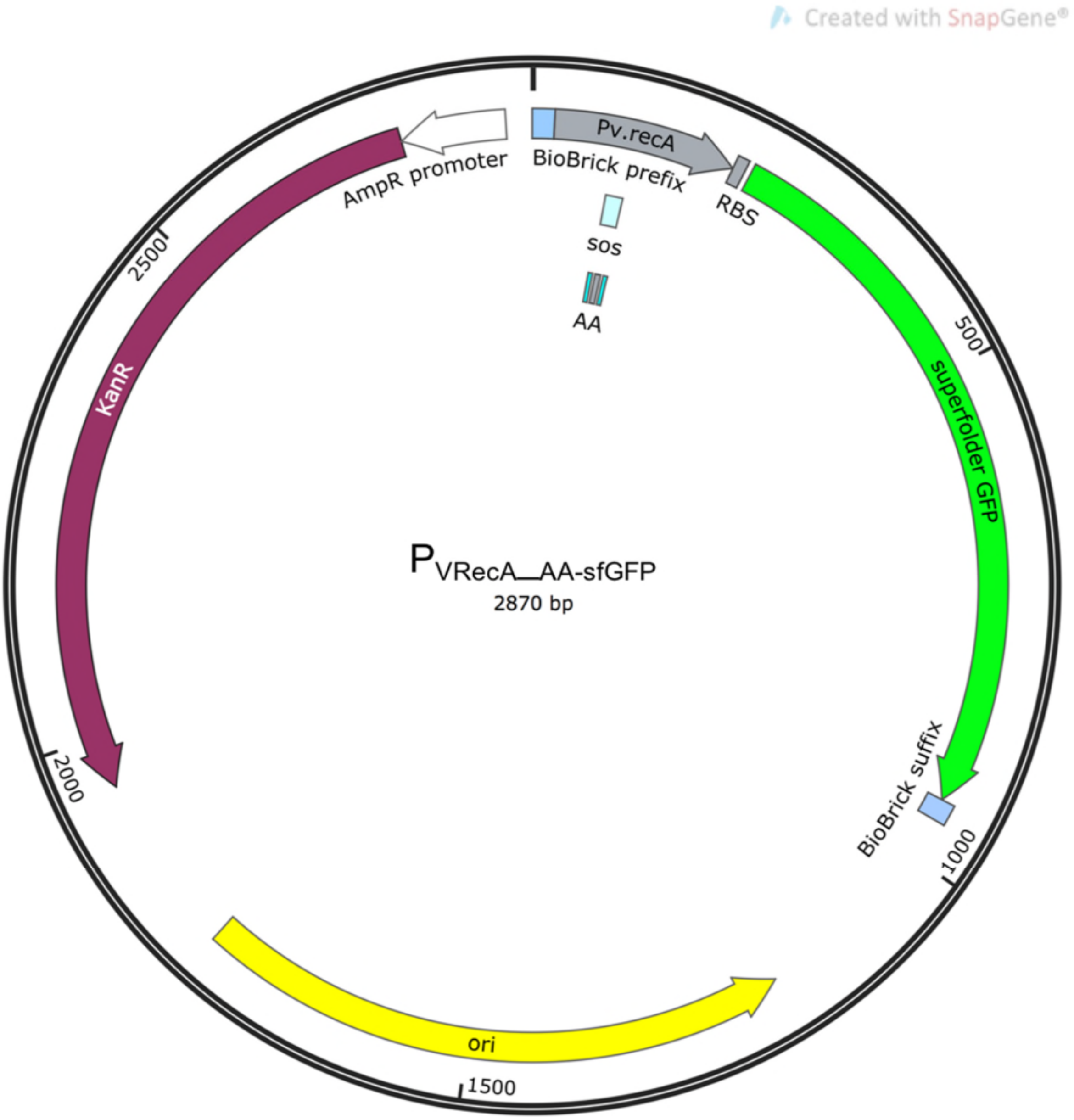
Created with SnapGene®
Pv.recA
BioBrick prefix
AmpR promoter
RBS
sos
AA
superfolder GFP
KanR
BioBrick suffix
ori
2500
500
2000
1000
1500
PVRecA_AA-sfGFP
2870 bp

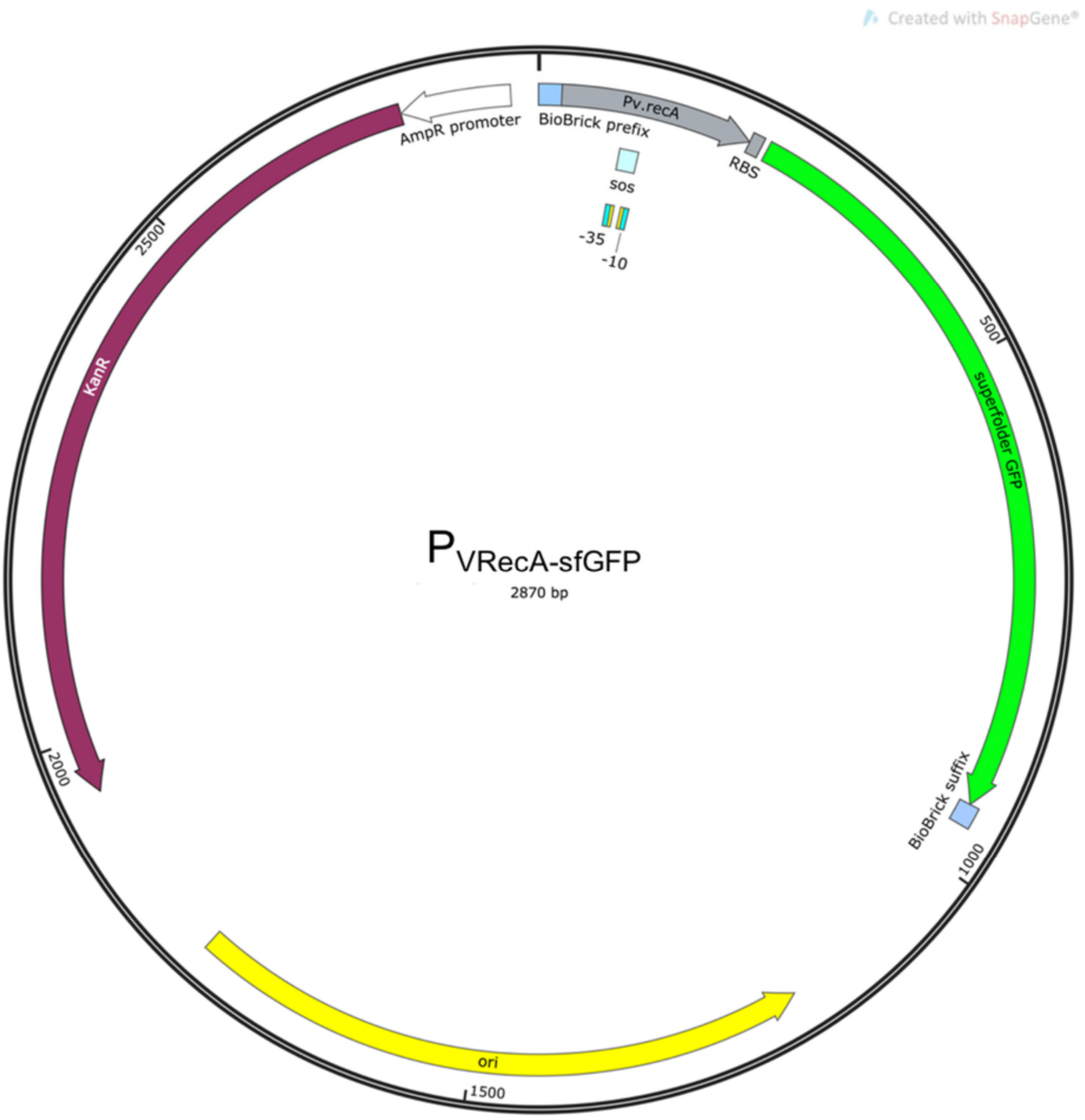
Created with SnapGene®
Pv.recA
BioBrick prefix
AmpR promoter
RBS
sos
-35
-10
KanR
superfolder GFP
2500
500
2000
1000
1500
BioBrick suffix
ori
PVRecA-sfGFP
2870 bp

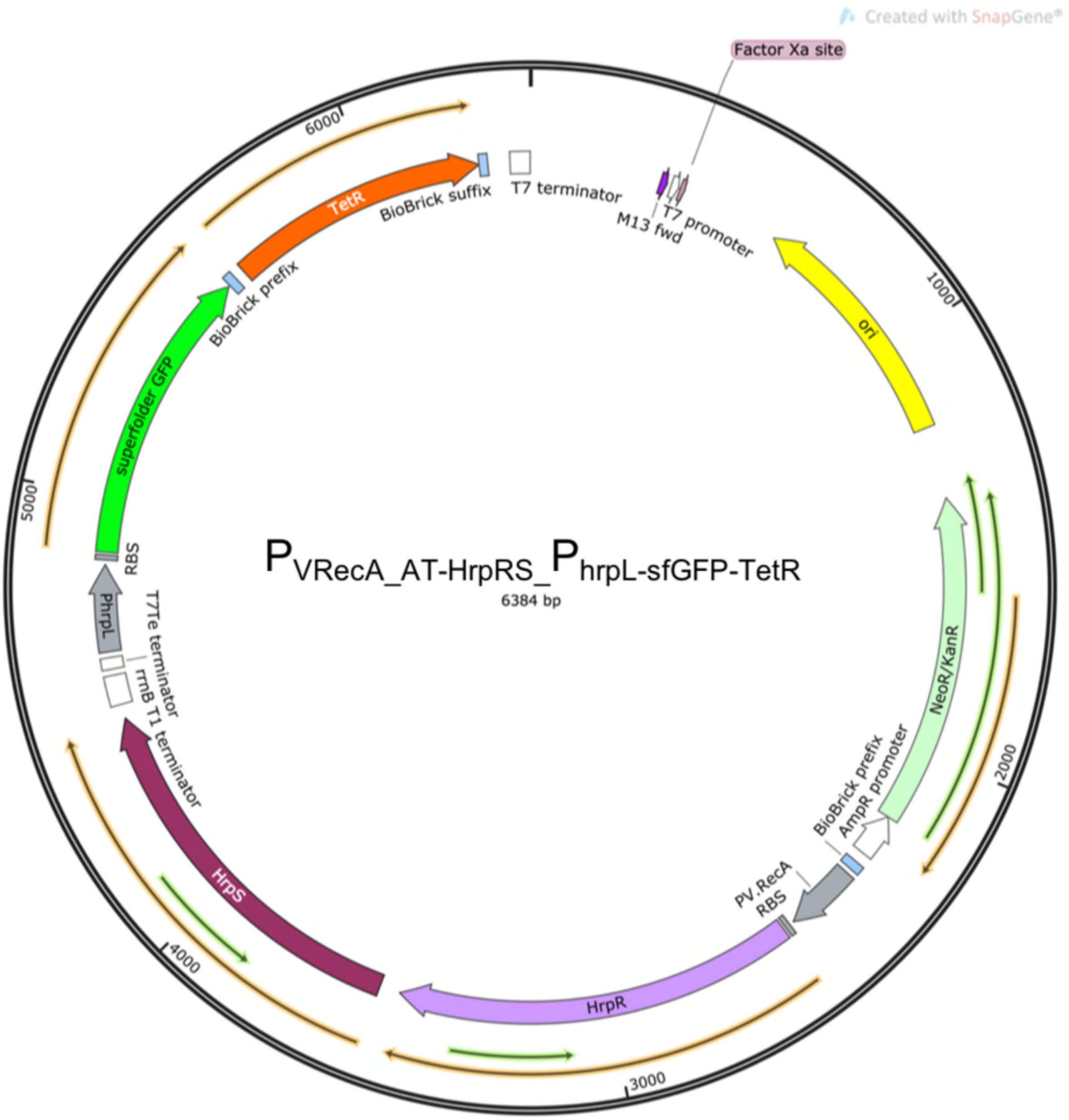
Created with SnapGene®
Factor Xa site
6000
TetR
BioBrick suffix
T7 terminator
M13 fwd
T7 promoter
1000
ori
BioBrick prefix
superfolder GFP
5000
RBS
PhrpL
T7Te terminator
rrnB T1 terminator
PVRecA_AT-HrpRS_PhrpL-sfGFP-TetR
6384 bp
NeoR/KanR
BioBrick prefix
AmpR promoter
2000
PV.RecA
RBS
HrpS
4000
HrpR
3000

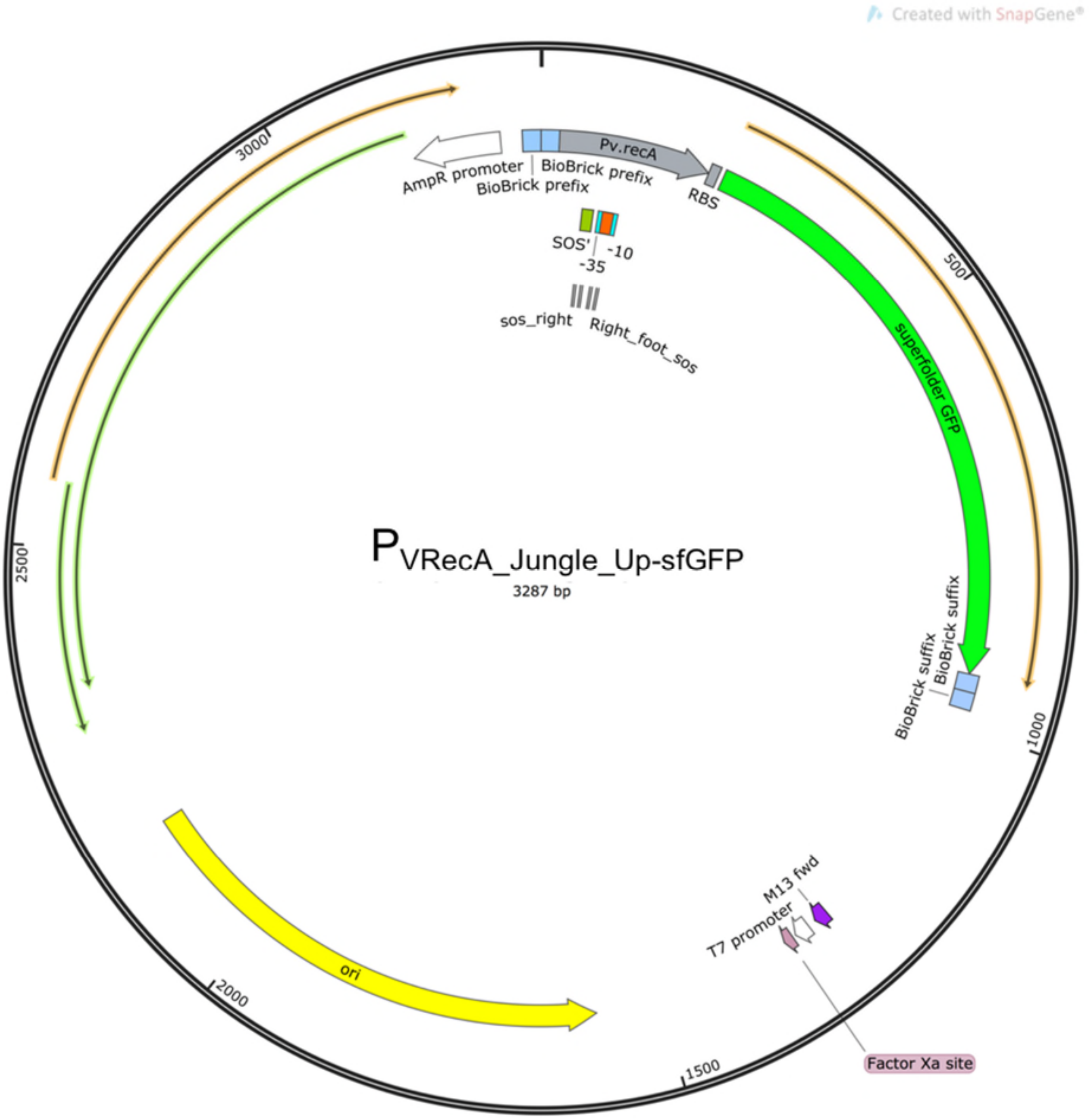

Created with SnapGene®
Pv.recA
AmpR promoter
BioBrick prefix
BioBrick prefix
RBS
SOS'
-10
-35
sos_right
Right_foot_sos
superfolder GFP
BioBrick suffix
BioBrick suffix
M13 fwd
T7 promoter
Factor Xa site
ori
PVRecA_Jungle_Up-sfGFP
3287 bp
500
1000
1500
2000
2500
3000

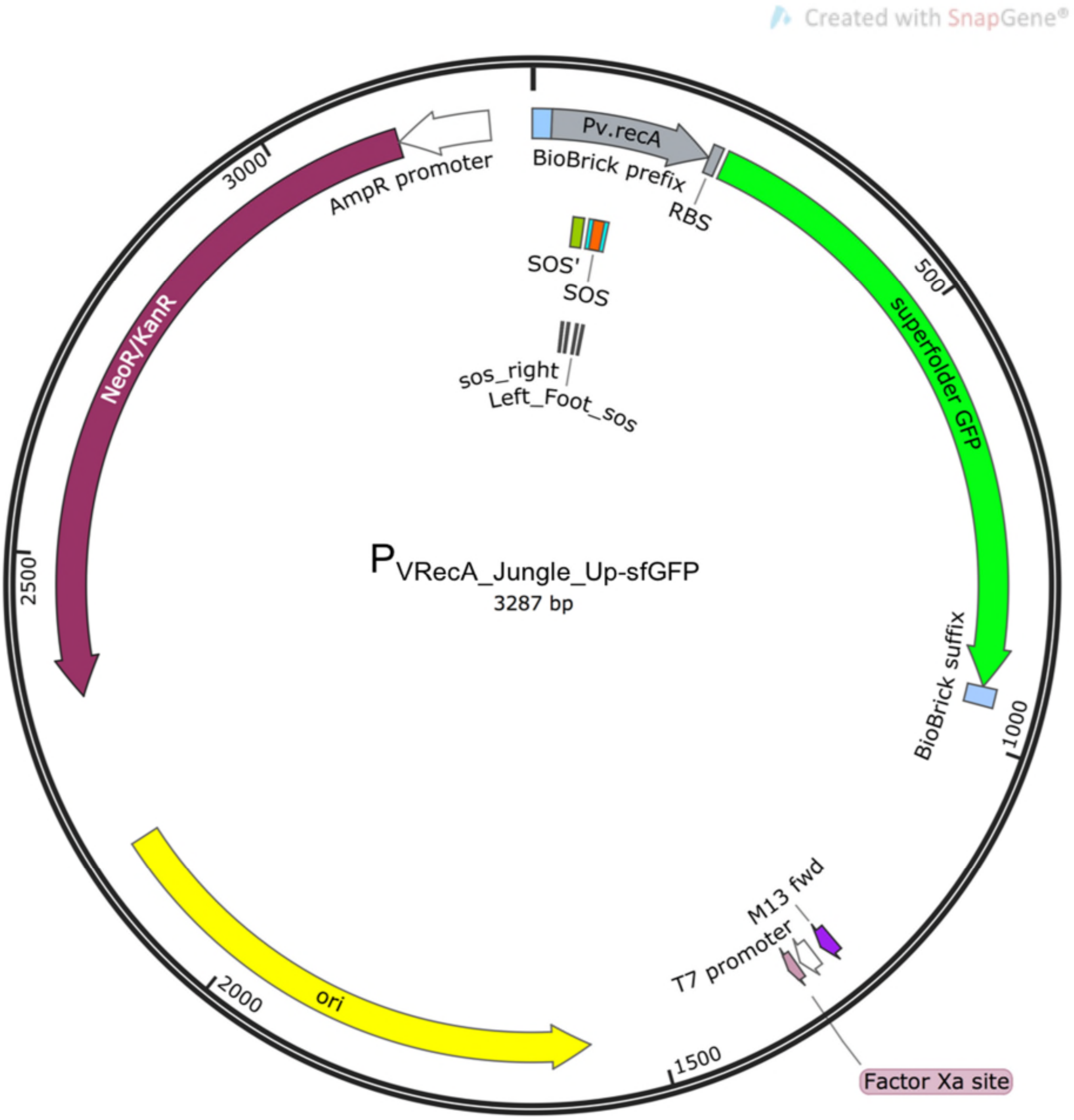
Created with SnapGene®
Pv.recA
BioBrick prefix
RBS
AmpR promoter
3000
SOS'
SOS
sos_right
Left_Foot_sos
500
superfolder GFP
NeoR/KanR
P VRecA_Jungle_Up-sfGFP
3287 bp
2500
BioBrick suffix
1000
M13 fwd
T7 promoter
2000
ori
1500
Factor Xa site

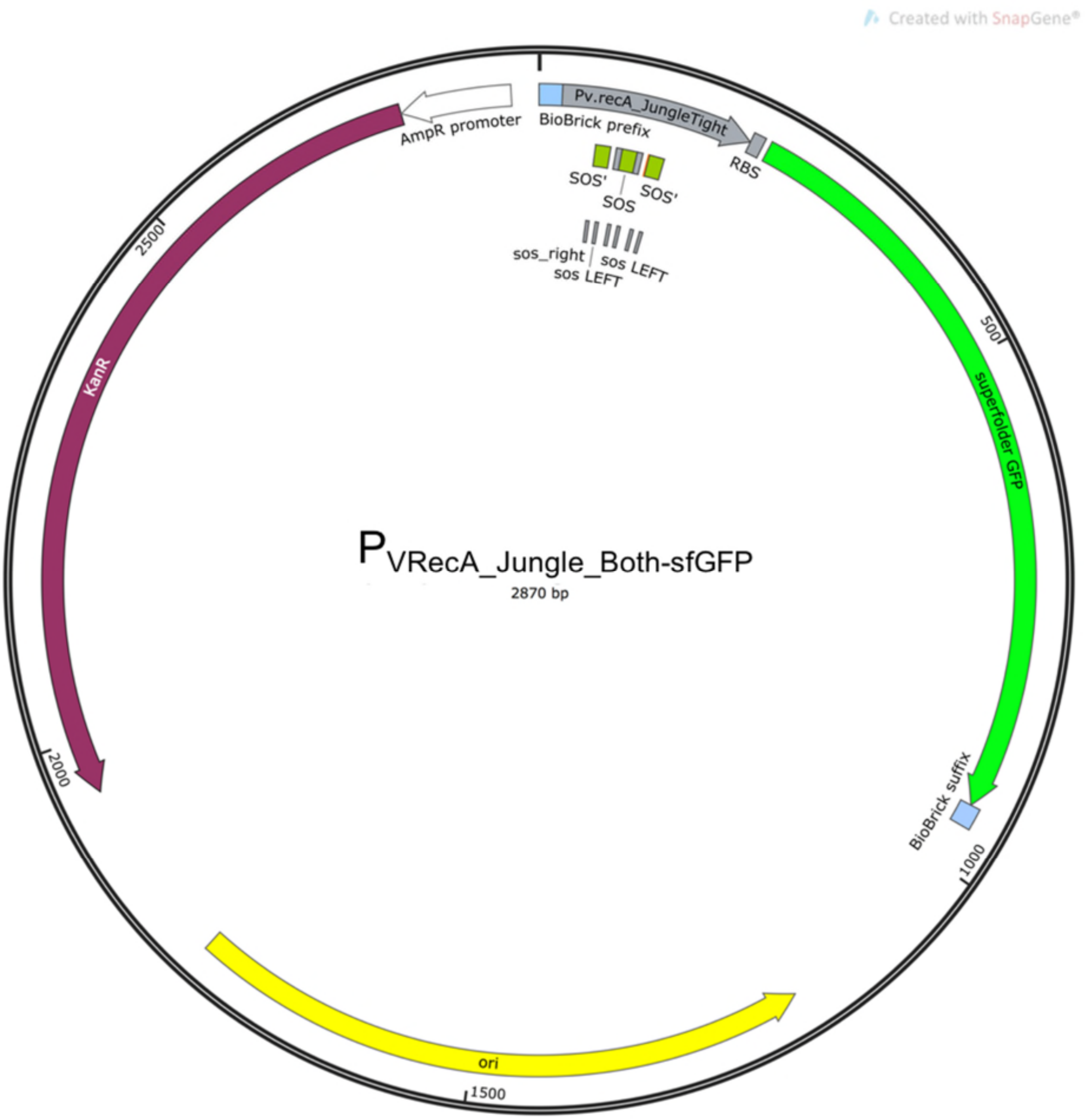
Created with SnapGene®
Pv.recA_JungleTight
BioBrick prefix
AmpR promoter
RBS
SOS'
SOS
SOS'
sos_right
sos LEFT
sos LEFT
KanR
superfolder GFP
2500
500
2000
1000
1500
BioBrick suffix
ori
P VRecA_Jungle_Both-sfGFP
2870 bp

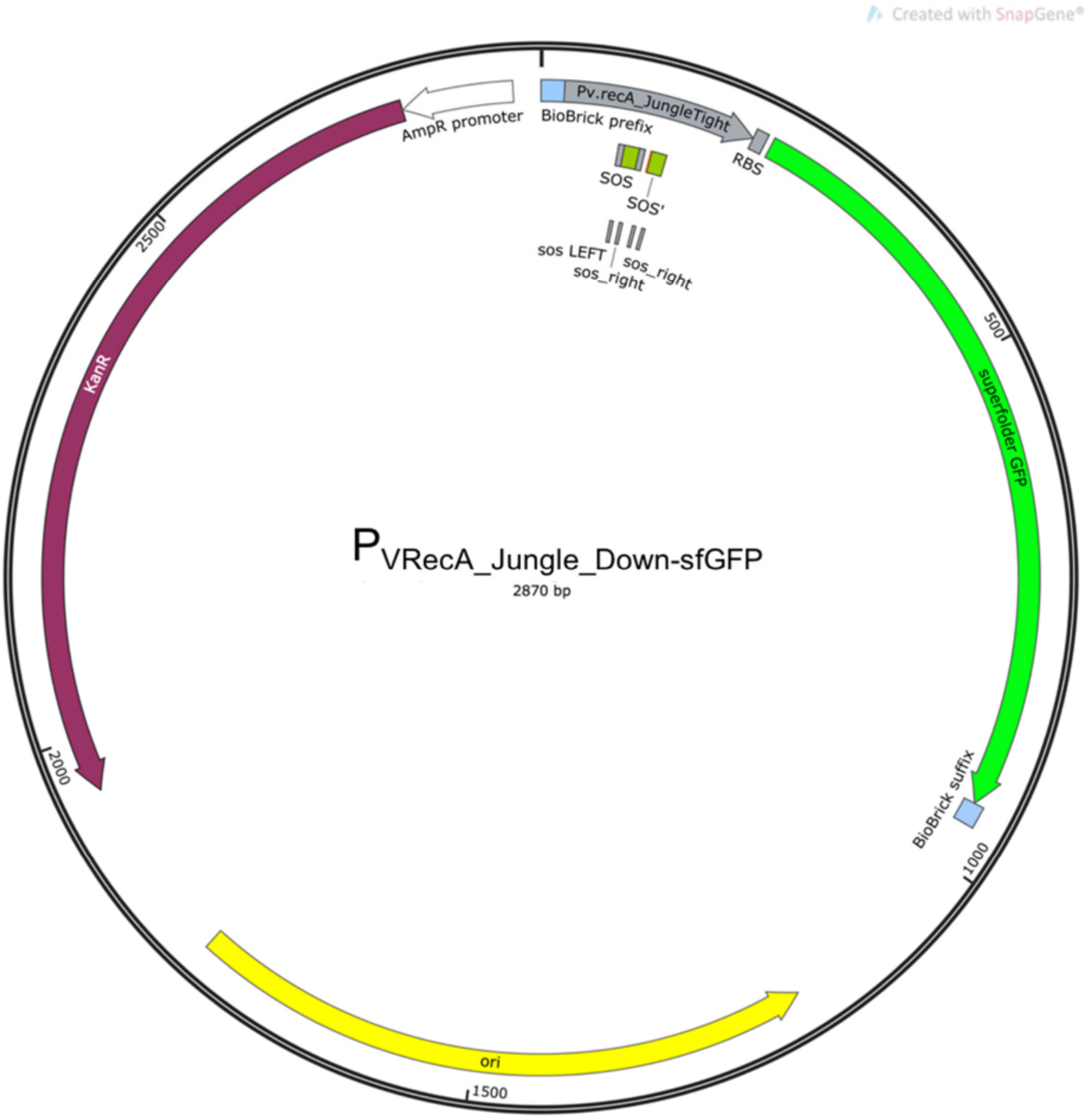
Created with SnapGene®
Pv.recA_JungleTight
BioBrick prefix
AmpR promoter
RBS
SOS
SOS'
sos LEFT
sos_right
sos_right
500
superfolder GFP
KanR
2500
PVRecA_Jungle_Down-sfGFP
2870 bp
2000
BioBrick suffix
1000
ori
1500

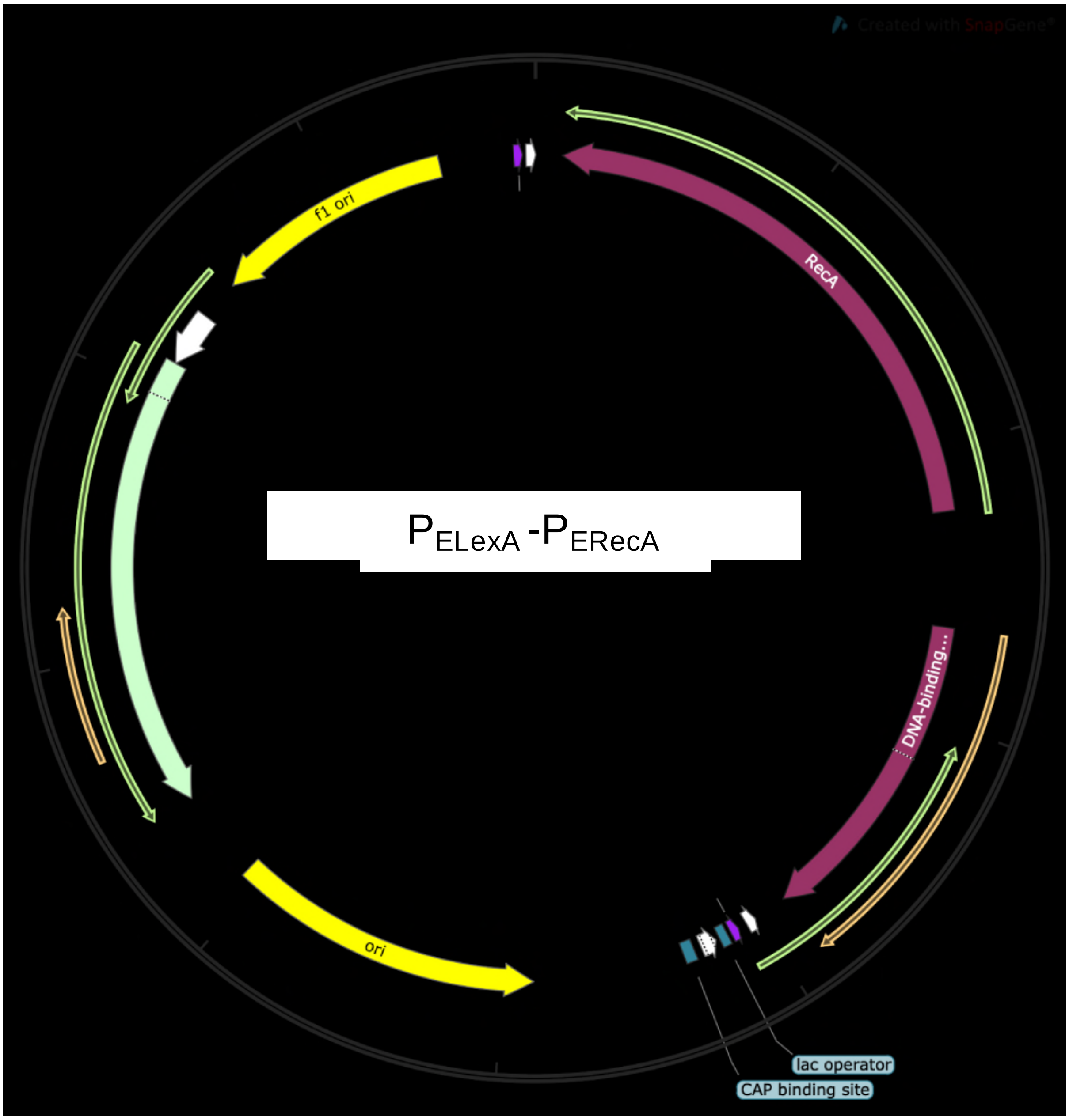
Created with SnapGene®
f1 ori
RecA
P_{ELexA} -P_{ERecA}
DNA-binding...
ori
lac operator
CAP binding site

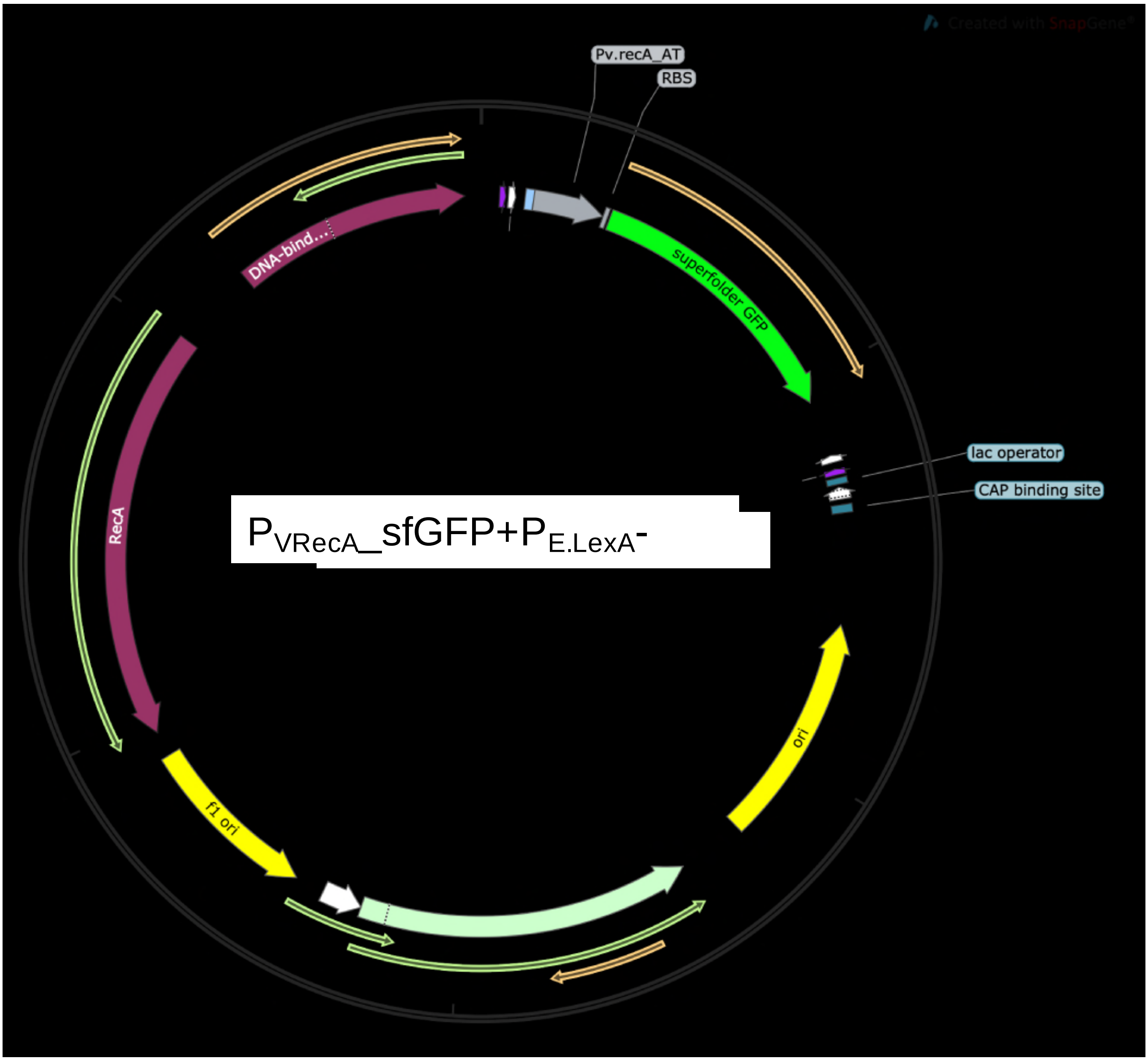
P_{VRecA}_sfGFP+$P_{E.LexA}$-
Pv.recA_AT
RBS
superfolder GFP
lac operator
CAP binding site
ori
f1 ori
RecA
DNA-bind...

Reference:

Brierley, J., Wong, C.S., Cummings, B., Catton, P., Ringash, J., Catton, C., et al. (2001) Squamous cell carcinoma of the oesophagus treated with radiation and 5-fluorouracil, with and without mitomycin C. *Clin Oncol*.

Cirz, R.T., O'Neill, B.M., Hammond, J.A., Head, S.R., and Romesberg, F.E. (2006) Defining the Pseudomonas aeruginosa SOS response and its role in the global response to the antibiotic ciprofloxacin. *J Bacteriol*.

Dryselius, R., Izutsu, K., Honda, T., and Iida, T. (2008) Differential replication dynamics for large and small Vibrio chromosomes affect gene dosage, expression and location. *BMC Genomics*.

Ganai, S., Arenas, R.B., and Forbes, N.S. (2009) Tumour-targeted delivery of TRAIL using Salmonella typhimurium enhances breast cancer survival in mice. *Br J Cancer* **101**: 1683–1691.

Ghosn, M., Kourie, H.R., Abdayem, P., Antoun, J., and Nasr, D. (2015) Anal cancer treatment: Current status and future perspectives. *World J Gastroenterol*.

Gibson, B., Wilson, D.J., Feil, E., and Eyre-Walker, A. (2018) The distribution of bacterial doubling times in the wild. *Proc R Soc B Biol Sci*.

Jacoby, G.A., Griffin, C.M., and Hooper, D.C. (2011) Citrobacter spp. as a source of qnrB alleles. *Antimicrob Agents Chemother*.

Jeong, C.W., Jeon, H.G., Kwak, C., Jeong, H., and Lee, S.E. (2005) Comparison of 30 mg and 40 mg of Mitomycin C Intravesical Instillation in Korean Superficial Bladder Cancer Patients: Prospective, Randomized Study. *Cancer Res Treat*.

Jian, H., Xiong, L., He, Y., and Xiao, X. (2015) The regulatory function of LexA is temperature-dependent in the deep-sea bacterium Shewanella piezotolerans WP3. *Front Microbiol*.

Lee, K.H. and Ruby, E.G. (1994) Effect of the squid host on the abundance and distribution of symbiotic Vibrio fischeri in nature. *Appl Environ Microbiol*.

Part:BBa B0030 - parts.igem.org.

Phan, N.Q., Uebanso, T., Shimohata, T., Nakahashi, M., Mawatari, K., and Takahashi, A. (2015) DNA-binding protein HU coordinates pathogenicity in Vibrio parahaemolyticus. *J Bacteriol*.

Purcell, O., Grierson, C.S., Bernardo, M.D., and Savery, N.J. (2012) Temperature dependence of ssrA-tag mediated protein degradation. *J Biol Eng*.

Rogers, J.K., Guzman, C.D., Taylor, N.D., Raman, S., Anderson, K., and Church, G.M. (2015) Synthetic biosensors for precise gene control and real-time monitoring of metabolites. *Nucleic Acids Res* **43**: 7648–7660.

Shaner, N.C., Campbell, R.E., Steinbach, P.A., Giepmans, B.N.G., Palmer, A.E., and Tsien, R.Y. (2004) Improved monomeric red, orange and yellow fluorescent proteins derived from Discosoma sp. red fluorescent protein. *Nat Biotechnol*.

Shaner, N.C., Steinbach, P.A., and Tsien, R.Y. (2005) A guide to choosing fluorescent proteins. *Nat Methods*.

Verweij, J. and Pinedo, H.M. (1990) Mitomycin C: mechanism of action, usefulness and limitations. *Anticancer Drugs*.

Wang, B., Barahona, M., and Buck, M. (2014) Engineering modular and tunable genetic amplifiers for scaling transcriptional signals in cascaded gene networks. *Nucleic Acids Res*.

Weinstock, M.T., Hesek, E.D., Wilson, C.M., and Gibson, D.G. (2016) Vibrio natriegens as a fast-growing host for molecular biology. *Nat Methods*.
